# An umbrella review of the literature on the effectiveness of psychological interventions for pain reduction

**DOI:** 10.1186/s40359-017-0200-5

**Published:** 2017-08-31

**Authors:** Georgios Markozannes, Eleni Aretouli, Evangelia Rintou, Elena Dragioti, Dimitrios Damigos, Evangelia Ntzani, Evangelos Evangelou, Konstantinos K. Tsilidis

**Affiliations:** 1 Department of Hygiene and Epidemiology, University of Ioannina School of Medicine, University Campus, 45110 Ioannina, Greece; 2 Lab of Cognitive Neuroscience, School of Psychology, Aristotle University of Thessaloniki, 54124 Thessaloniki, Greece; 3 Department of Psychiatry, University of Ioannina School of Medicine, University Campus, 45110 Ioannina, Greece; 4 Center for Evidence Synthesis in Health, Department of Health Services, Policy and Practice, School of Public Health, Brown University, Providence, Rhode Island 02903 USA; 5 Department of Epidemiology and Biostatistics, School of Public Health, Imperial College London, London, SW7 2AZ UK

**Keywords:** Pain, Pain management, Psychology, Psychological interventions, Umbrella review

## Abstract

**Background:**

Psychological interventions are widely implemented for pain management and treatment, but their reported effectiveness shows considerable variation and there is elevated likelihood for bias.

**Methods:**

We summarized the strength of evidence and extent of potential biases in the published literature of psychological interventions for pain treatment using a range of criteria, including the statistical significance of the random effects summary estimate and of the largest study of each meta-analysis, number of participants, 95% prediction intervals, between-study heterogeneity, small-study effects and excess significance bias.

**Results:**

Thirty-eight publications were identified, investigating 150 associations between several psychological interventions and 29 different types of pain. Of the 141 associations based on only randomized controlled trials, none presented strong or highly suggestive evidence by satisfying all the aforementioned criteria. The effect of psychological interventions on reducing cancer pain severity, pain in patients with arthritis, osteoarthritis, rheumatoid arthritis, breast cancer, fibromyalgia, irritable bowel syndrome, self-reported needle-related pain in children/adolescents or with chronic musculoskeletal pain, chronic non-headache pain and chronic pain in general were supported by suggestive evidence.

**Conclusions:**

The present findings reveal the lack of strong supporting empirical evidence for the effectiveness of psychological treatments for pain management and highlight the need to further evaluate the established approach of psychological interventions to ameliorate pain.

**Electronic supplementary material:**

The online version of this article (10.1186/s40359-017-0200-5) contains supplementary material, which is available to authorized users.

## Background

Chronic pain is a common medical condition that causes significant distress and disability [1]. The prevalence of chronic pain in adults, defined as lasting for at least 6 months, is estimated in the range of 10% to 55% depending on age, sex, setting and type of chronic pain with a weighted mean prevalence of 31% in US adults, and is consistently reported to be higher in women [2, 3]. Psychological interventions, either alone or in combination with pharmacological treatments, are widely recommended for pain management and treatment [4]. Psychological therapies consist of behavioural and cognitive treatments that are designed to ameliorate pain, distress and disability.

Psychological interventions were introduced over 40 years ago and are now well established in clinical practice [5]. Several randomized controlled trials (RCTs) but also uncontrolled trials, observational studies, and clinical case reports have suggested a positive effect of psychological interventions on pain management, although the reported effect sizes vary widely [6]. Moreover, narrative reviews have generally supported the effectiveness of psychological treatments on a range of pain conditions [7–9]. Meta-analyses and systematic reviews have provided additional evidence for the effectiveness of psychological treatments in the management of chronic pain [10–12]. However, the effect sizes across all meta-analyses are modest, only rising above a medium-size effect (i.e., standardised mean difference larger than 0.5) in lower quality studies [4]. The effectiveness of psychological treatments is shown to be over-estimated in poorly designed studies, and is reduced when controlled for quality and adjusted for potential bias [4, 13]. Thus, the reported heterogeneity in effect sizes is partly explained by the quality of the studies [13]. This observation is indicative of the possibility of bias in this literature, which could be due to publication or other selective reporting biases, where study authors employ several data collection and analysis techniques but publish only the most statistically significant findings [14–18]. Because of the wide implementation of psychological interventions in pain management and the elevated likelihood for biases in this field as shown in prior relevant empirical research [19, 20], we used an umbrella review approach [21, 22] that systematically appraises the evidence on an entire field across many meta-analyses. In the present study we aimed to broaden the scope of a typical umbrella review by further evaluating the strength of the evidence and the extent of potential biases [23–27] on this body of literature.

## Methods

### Literature search and data extraction

We identified all relevant meta-analyses investigating the association of psychological interventions on pain management. We searched PubMed (until July 2016) and the Cochrane (until September 2016) database of systematic reviews for papers written in English, performed in humans using the following three keywords: “pain”, “meta-analysis” and “psychology”. In addition, we performed a manual review of references from available systematic and narrative reviews. In total, 987 publications were identified in the electronic databases and additional 29 via manual review. Two investigators (GM and ER) examined independently the titles, abstracts and full texts of the shortlisted meta-analyses to decide on eligibility. Discrepancies were resolved by consensus and with discussion with a third investigator (KKT). We considered all age groups (i.e., children, adolescents and adults) and all types of pain, and examined the effect of psychological interventions both at short and long-term periods. Meta-analyses that did not report study-specific information (i.e., effect size, 95% confidence intervals [CIs], sample size) were excluded. When more than one meta-analysis on the same research question was identified, the one with the largest number of component studies was selected. Only seven meta-analyses were excluded by this criterion, all of them being substituted with updated meta-analyses published from the same author teams, thus no potentially relevant study was omitted. Two investigators (GM and ER) extracted independently the data from each meta-analysis, and a third investigator (ED) verified the validity of the extracted data. Information was abstracted from each study at the meta-analysis and individual study level. At the meta-analysis level, we abstracted information on first author, year of publication, examined interventions, outcomes, and number of included studies. At the individual study level, we abstracted information on study design, quality assessment/risk of bias score, sample size, effect estimate (i.e., mean difference [MD]; standardised mean difference [SMD]; risk ratio), and 95% CIs. For consistency, risk ratios and the corresponding CIs were converted into SMDs [28]. Positive and negative effect sizes were observed across the different meta-analyses because different outcome metrics were used, but all summary effect sizes were coined to express pain reduction. For example, assuming that a psychological intervention reduces pain, one can expect a positive effect in a meta-analysis examining the efficacy of the intervention in pain reduction, and a negative effect in another meta-analysis examining the difference in pain levels between intervention and control groups. In the current umbrella review, the primary analysis focused only in meta-analyses of RCTs and sensitivity analysis was performed including all study designs. Our study was conducted in accordance with guidelines for conducting and reporting umbrella reviews [21, 22].

### Types of interventions and outcomes considered

Meta-analyses of psychological interventions with a variety of theoretical underpinnings were considered. Any type of *cognitive* intervention such as hypnosis, guided imagery and distraction, and any type of *behavioural* intervention, such as biofeedback and relaxation, as well as their combinations were included [29]. All types of psychotherapy and psycho-education were also included in our umbrella review, whereas meta-analyses of other non-formal psychological interventions, such as acupuncture, massage, yoga and meditation were excluded. Interventions on single patients, pairs or families, either by physical contact between the therapist and the subjects, or by utilizing web-based platforms were considered. Some studies assessed the effectiveness of a single technique, such as biofeedback, whereas others assessed the effectiveness of a comprehensive psychological approach, such as Cognitive Behavioural Therapy. A complete list of interventions considered in our umbrella review is shown on Table 1, which illustrates the complete list of included studies.

**Table 1:** Characteristics of the 38 included meta-analysis papers

Author, Year	List of Interventions evaluated	List of Comparison groups	Type of pain	Number of included meta-analyses in this umbrella review^a^	Primary studies per included meta-analysis^b^	Sample size per included meta-analysis^b^
Adachi T, 2013	• Hypnosis	• Standard care • Other psychological treatments	• Chronic	3	4 to 12	163 to 505
Aqqarwal VR, 2011	• Any psychosocial intervention • Cognitive behavioural therapy only • Biofeedback only • Cognitive behavioural therapy + Biofeedback • Hypnotherapy	• Usual treatment • Relaxation	• Muscle palpation • Orofacial	7	2 to 4	45 to 411
Bawa F, 2015	• Mindfulness	• Active control • Inactive Control	• Chronic pain intensity	2	4 to 5	104 to 349
Bernardy K, 2011	• Hypnotherapy	• Cognitive behavioural therapy/Treated as usual/Waiting list/Attention placebo	• Fibromyalgia	1	6	178
Bernardy K, 2013	• Cognitive behavioural therapy • Operant therapy • Self-management	• Active control/Attention control/Education/Treated as usual/Support	• Fibromyalgia	9	2 to 18	123 to 1150
Birnie K, 2014	• Distraction	• Not Reported	• Needle-related (children, adolescents)	1	24	2472
Champaneria, 2012	• Psychological intervention	• No psychological intervention	• Chronic pelvic	2	2 to 2	139 to 156
Damen L, 2006	• Relaxation + Biofeedback + Cognitive behavioural therapy • Relaxation + Biofeedback • Relaxation + Cognitive behavioural therapy • Biofeedback	• Control • Attention placebo • Waiting list	• Headache	4	2 to 3	44 to 71
Dixon K, 2007	• Cognitive behavioural therapy/Stress management/Hypnotherapy	• Not Reported	• Arthritis	1	20	2303
Du S, 2011	• Arthritis Self-Management Program/Self-management	• Waiting list/Usual care/Conventional/No treatment	• Chronic musculoskeletal	3	3 to 8	1018 to 2968
Eccleston C, 2014a	• Psychological therapies (Internet-delivered)	• Active control/Treated as usual/Waiting list	• Chronic Non- headache • Chronic headache	3	2 to 11	131 to 1785
Eccleston C, 2014b	• Psychological therapies	• Control	• Chronic and recurrent non-headache (children, adolescents) • Chronic and recurrent headache (children, adolescents)	4	5 to 15	251 to 852
Fisher E, 2014	• Cognitive behavioural therapy/Biofeedback/ Relaxation/ Hypnotherapy	• Waiting list/Education/Standard care/ Self-monitoring	• Chronic Non- headache • Chronic headache	2	11 to 18	672 to 748
Flanagan E, 2015	• Cognitive behavioural therapy only • Cognitive behavioural therapy + behavioural	• Other psychological treatment • Waiting list • Medical treatment	• General vaginal pain • Pain on intercourse	6	2 to 3	83 to 148
Glombiewski JA, 2010	• Education/ Cognitive behavioural therapy/ Relaxation	• Cognitive behavioural therapy/Treated as usual/Waiting list/Attention placebo	• Fibromyalgia	1	21	1017
Guzman J, 2002	• Multidisciplinary bio-psychosocial rehabilitation program	• Not Reported	• Low back	8	2 to 4	142 to 442
Henrich J, 2015	• Psychological therapies	• Control	• Irritable bowel syndrome	1	32	2245
Henschke N, 2011	• Behavioural treatment • Behavioural treatment + physiotherapy • Cognitive behavioural therapy • Cognitive therapy • Operant therapy • Respondent therapy	• Usual care • Group exercise • Physiotherapy • Cognitive therapy • Operant therapy • Respondent therapy • Waiting list	• Chronic low back, IT	23	2 to 5	44 to 405
Johannsen M, 2013	• Education/ Relaxation, guided imagery, meditation or hypnosis / Supportive group therapy	• Waiting list/Standard care/Not Reported	• Breast cancer (patients/survivors)	1	21	1770
Kamper SJ, 2014	• Multidisciplinary biopsychological rehabilitation	• Usual care • Physical treatment • Surgery • Waiting list	• Chronic low back	8	2 to 12	213 to 1661
Kisely SR, 2015	• Psychological intervention	• No psychological intervention	• Chest	5	2 to 7	111 to 294
Knittle K, 2010	• Self-regulation	• Waiting list/Standard care/No Intervention	• Rheumatoid Arthritis	1	22	1316
Koranyi S, 2014	• Psychological intervention	• Control • Treated as usual	• Acute pain after open heart surgery	3	3 to 4	280 to 413
Kroon FP, 2014	• Self-management education programmes	• No Self-management education programmes • Information • Usual care/Waiting list/No treatment	• Osteoarthritis	6	2 to 13	118 to 2271
Lakhan S, 2013	• Mindfulness-based therapy/ Mindfulness-based cognitive therapy	• Education/Waiting list/Support	• Fibromyalgia • Irritable bowel syndrome	2	2 to 4	160 to 276
Lauche R, 2013	• Mindfulness-based stress reduction	• Active control • Usual care	• Fibromyalgia Syndrome	4	2 to 3	174 to 323
Macea DD, 2010	• Web-based Cognitive behavioural therapy interventions	• Control	• Chronic pain	1	11	2958
Mustafa M, 2013	• Supportive/expressive group therapy	• Usual treatment	• Metastatic breast cancer	1	3	279
Osborn RL, 2006	• Education	• Control	• Cancer survivors	1	3	250
Peerdeman K, 2016	• Verbal suggestion/ Imagery • Verbal suggestion only • Conditioning only • Imagery only	• Control/No treatment	• Affective pain • Expected pain • Pain relief	5	3 to 18	142 to 1061
Roldan-Barraza C, 2014	• Psychosocial Intervention/ Psychosocial Intervention + Usual Treatment	• Usual Treatment • Tailored Usual Treatment	• Myofascial Temporomandibular Disorder	5	3 to 7	238 to 470
Sheinfeld Gorin S, 2012	• Psychological intervention	• Control	• Cancer pain severity	1	38	4270
Sielski R, 2016	• Biofeedback/ Electromyographic Biofeedback	• Control	• Chronic back	2	11 to 22	471 to 1059
Sprenger L, 2011	• Psychoeducation/Imagination/Relaxation/Biofeedback/Cognitive behavioural therapy	• No treatment/Paediatric standard care	• Recurrent abdominal in children	1	9	449
Theadom A, 2015	• Psychological therapies • Mindfulness • Relaxation	• Usual care • Attention control	• Fibromyalgia	7	2 to 9	67 to 453
Uman LS, 2013	• Child distraction • Cognitive behavioural therapy-combined • Hypnosis • Parent coaching + child distraction • Preparation and information • Suggestion • Virtual reality	• Control • Standard care	• Needle-related (children, adolescents)	8	2 to 5	50 to 612
Vellemain S, 2010	• Computerized Cognitive behavioural therapy	• Waiting list/ Education	• Pain in children and adolescents	1	4	150
Williams AC, 2012	• Behavioural • Cognitive behavioural	• Treated as usual • Active control	• Chronic non- headache	6	2 to 16	182 to 1335

^a^Number of included meta-analyses may differ from the number of combinations of intervention group, control group and outcome because I) some possible combinations were not assessed in original studies, and II) there are instances where the outcome was evaluated in different time points. For a complete list of the combinations included in this umbrella review please refer to Additional file 1: Table S1

^b^When more than one meta-analysis is included per study, numbers represent minimum-maximum

### Assessment of summary effects and heterogeneity

In the present umbrella review, both fixed and random effects meta-analysis methods were applied. Fixed effect meta-analysis is based on the assumption that every study in the meta-analysis is estimating the one true underlying effect and that the observed differences and heterogeneity thereof is due to chance alone. A random effect meta-analysis is based on the assumption that every study is estimating a different underlying effect and that all these effects follow a distribution. In order to test for between-study heterogeneity, we implemented the χ^2^-based Cochran Q test [30] and the I^2^ metric of inconsistency [31], which is defined as the ratio of between-study variance over the sum of the within-study and between-study variances. The I^2^ metric takes values between 0 and 100 and represents the percentage of the variability in the effect sizes that is due to between-study heterogeneity. I^2^ values of 25%, 50%, and 75% indicate low, moderate, and large heterogeneities, respectively. Ninety-five percent prediction intervals were also calculated, which further take into account the between-study heterogeneity and estimate the effect that would be expected in a future study investigating the same association [32, 33].

### Assessment of small-study effects

The assessment of small-study effects was used to investigate whether smaller studies tend to give larger effect estimates compared to larger studies. Differences between small and large studies can reflect genuine heterogeneity, chance or biases. The regression asymmetry test, as proposed by Egger, was used to evaluate small-study effects [34, 35]. Based on the test, a *p*-value smaller than or equal to 0.10, along with the random effects summary estimate being inflated compared to the point estimate of the largest study in the meta-analysis, were an indication of small study effects. Effect magnitude asymmetry may arise due to several reasons, such as true heterogeneity, publication biases or chance, but the asymmetry test can only indicate its existence and cannot distinguish the reason behind it. However if the asymmetry is assumed to be a product of bias, the extrapolation of the Egger’s regression line to a zero standard error, which corresponds to a theoretical study of infinite size, can be regarded as an estimation of the effect size that is free from biases [35–37].

### Evaluation of excess statistical significance

The excess statistical significance test was performed to investigate whether the observed number of studies with nominally statistically significant results (*P* < 0.05) is greater compared to an expected number of studies with statistically significant results [38]. An excess of statistical significant findings in a meta-analysis may imply the presence of selective reporting bias, as many underpowered studies with statistically significant results may be identified in the field. The sum of the statistical power estimates for each component study in a meta-analysis was used to calculate the expected number of studies with statistically significant results. The power of each individual component study depends on the effect size that the tested psychological intervention has on pain. The actual size of the true effect is not known but was estimated in the current umbrella review using the effect size of the largest study (i.e., smallest standard error) in each meta-analysis [38, 39]. The statistical power of each study was calculated using the *power* command in Stata (College Station, TX). Excess statistical significance was claimed if *P* < 0.10 (one-sided *p* < 0.05 with observed > expected number of studies with statistically significant results).

### Quality of the included studies

We assessed the methodological quality of the included meta-analyses using the assessment of multiple systematic reviews (AMSTAR) tool [40]. We categorised the study quality based on the overall AMSTAR score as high (8-11 items achieved), moderate (4-7 items) and low (0-3 items). We further gathered any quality assessment/risk of bias score information pertaining to the primary studies, based on what the meta-analyses reported.

### Grading the evidence

Using the criteria mentioned above, associations that presented nominally statistically significant random effects summary estimates (i.e., *P* < 0.05) were categorised into strong, highly suggestive, suggestive, or weak evidence, following a grading scheme that has already been applied in various fields [23–27]. A strong association was claimed when the *p*-value of the random effects meta-analysis was smaller than 10^−6^, the meta-analysis had more than 1000 participants, the largest study in the meta-analysis was nominally statistically significant (i.e., *P* < 0.05), the I^2^ statistic of between study heterogeneity was smaller than 50%, the 95% prediction intervals were excluding the null value, and there was no indication of small study effects or excess significance bias. The criteria for a highly suggestive association were met if: *P* < 10^−6^, >1000 participants, and largest study in the meta-analysis presenting nominally significant estimate (i.e., *P* < 0.05). An association was supported by suggestive evidence if the meta-analysis included more than 1000 participants and the random effects P was smaller than 10^−3^. All other nominally statistically significant associations (i.e., *P* < 0.05) were deemed to have weak evidence.

The vast majority of the primary trials in the meta-analyses included very small numbers of participants. However, as the majority of these trials are randomized experiments one would expect to see valid estimates even with lower sample sizes. We conducted a sensitivity analysis by lowering the threshold for the number of participants in a meta-analysis, as a method of checking the robustness of our evidence grading approach. Therefore, we reclassified all associations using a sample size threshold of more than 500 participants instead of 1000. All analyses were performed using Stata version 13 (College Station, TX) [41].

## Results

### Description of meta-analyses

**Fig. 1:**
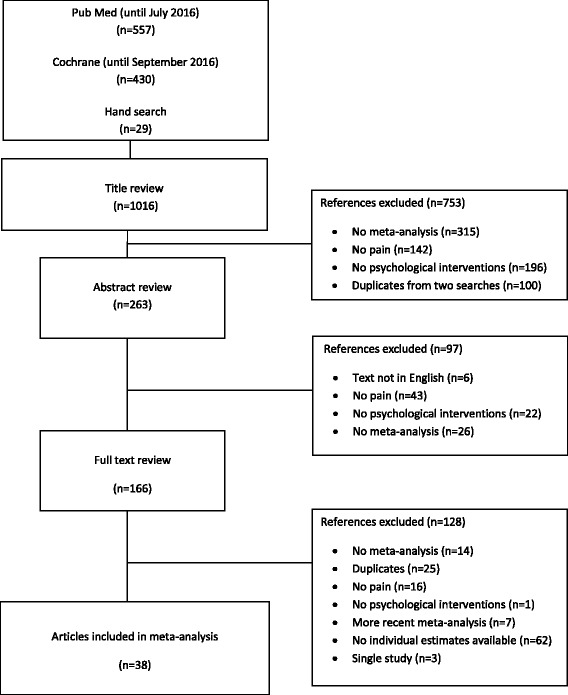
Flow chart of literature selection

Of the 1016 articles initially identified, 38 papers [6, 10, 11, 13, 42–75] including 150 meta-analyses models with 865 individual study estimates were finally selected (Table 1 and Fig. 1). These studies included associations between several psychological interventions (comprehensive therapies or single techniques) and 29 different types of pain (i.e., acute pain, affective pain, arthritis, breast cancer, cancer in general, cancer pain severity, chest, chronic and recurrent, chronic back, chronic low back, chronic musculoskeletal, chronic pain, chronic pelvic, expected pain, fibromyalgia, headache, irritable bowel syndrome, low back, muscle pain, muscle palpation, myofascial temporomandibular disorder, needle-related pain in children and adolescents, orofacial, osteoarthritis, pain on intercourse, pain relief, recurrent abdominal, rheumatoid arthritis, vaginal pain). Of the 865 individual studies included in this umbrella review, 741 (85.7%) were randomized controlled trials, 42 (4.9%) were non-randomized controlled trials or clinical controlled trials, 6 (0.7%) were quasi-RCTs, 4 (0.5%) were uncontrolled pre-post clinical trials, whereas for 72 studies this information was not reported. The evaluation of all 150 meta-analyses of the 865 individual studies is presented in detail on Additional file 1: Tables S1 and S2, but the critical appraisal of the evidence from now on focuses only on associations from the 141 meta-analyses using only RCTs that are summarized on Additional file 1: Tables S3 and S4. There were 2 to 38 individual studies combined per meta-analysis with a median of 3 studies. The median number of participants in the intervention and control groups in each meta-analysis were 115 and 107, respectively. The smallest total sample size in a meta-analysis was 44 and the largest was 4270.

### Summary effect size

Out of the 141 meta-analyses including only randomized evidence (Additional file 1: Table S3), the summary random effects estimates were statistically significant at the *P* = 0.05 level in 56 (40%) meta-analyses, whereas the summary fixed effects were significant in 75 (53%) meta-analyses. Reductions in pain were observed in all statistically significant meta-analyses comparing the intervention to the control group. When the *P* = 0.001 level was used as a threshold for statistical significance, only 28 (20%) and 47 (33%) meta-analyses remained statistically significant using the random and fixed effects method, respectively. Only four associations on psychological interventions for cancer pain severity, irritable bowel syndrome, headache, and chronic headache in children produced statistically significant results when a *P* value of 10^−6^ was used as the significance threshold based on the random effects model. The effect of the largest study included in each meta-analysis is also presented in Table S3, which was nominally statistically significant in only 41 (29%) out of the 141 meta-analyses. The findings from the largest studies were more conservative than the summary estimates in 65 (46%) comparisons. Finally, most of the largest studies in each meta-analysis (*n* = 103; 73%) suggested effects of small or small-to-medium magnitude (i.e., SMD < 0.5), and similar magnitudes were observed in the majority of the summary random effects estimates (*n* = 98; 70%). When 95% prediction intervals were calculated, the null value was excluded in only 9 meta-analyses that investigated psychological interventions for pain management in patients with irritable bowel syndrome, fibromyalgia, osteoarthritis, rheumatoid arthritis, arthritis and headache (Additional file 1: Table S3).

### Between-study heterogeneity

Τhe Q test showed statistically significant heterogeneity (*P* ≤ 0.10) in 58 (42%) meta-analyses (Additional file 1: Table S4). There was moderate to high heterogeneity (I^2^ = 50%-75%) in 34 (24%) meta-analyses and very high heterogeneity (I^2^ > 75%) in 25 meta-analyses (18%) of eight different types of pain (i.e., chest pain frequency; chronic low back pain; chronic pain-excluding headache; needle-related pain/distress in children and adolescents; chronic pelvic pain; headache; fibromyalgia; pain on intercourse). Uncertainty around the heterogeneity estimates was often large, as reflected by wide 95% CI of the I^2^ (Additional file 1: Table S4).

### Small study effects and excess significance bias

There was not substantial evidence for presence of small study effects according to the Egger’s regression asymmetry test. Only in eight out of 141 (6%) meta-analyses, the *p*-value was smaller than 0.10 and the effect of the largest study was more conservative than the summary effect estimate. Nominally statistically significant summary estimates were calculated only for five associations (4%) after extrapolating the Egger regression line on a funnel plot to an infinitively large study (Additional file 1: Table S4). Ten meta-analyses (7%) (i.e., pain in breast cancer patients and survivors, cancer pain severity, chronic pain-excluding headache; self-reported needle-related in children and adolescents for two different interventions; low back pain; chronic lows back pain for two different interventions, frequency of chest pain, and irritable bowel syndrome pain) had evidence of statistically significant excess of “positive” studies, when the plausible effect was assumed to be equal to the effect of the largest study in each meta-analysis (Additional file 1: Table S4). An excess of significant findings in a meta-analysis coupled with an indication of small study effects based on Egger’s *p*-value can provide further evidence for the presence of selective reporting biases in the field. Only two meta-analyses presented indication for both excess significance and small study effects bias.

### Grading the evidence

None of the examined associations could claim either strong (random effects *P* < 10^−6^, > 1000 participants, statistically significant largest study, the I^2^ < 50%, the 95% prediction intervals were excluding the null value, and no indication of small study or excess significance bias) or highly suggestive (random effects *P* < 10^−6^, > 1000 participants, statistically significant largest study) evidence (Table 2). Twelve associations (i.e., cancer pain severity, pain from breast cancer; chronic musculoskeletal pain at 4 and 6 months follow-up; chronic pain; arthritis; osteoarthritis, rheumatoid arthritis; fibromyalgia; self-reported needle-related pain in children and adolescents; chronic non-headache pain; irritable bowel syndrome pain) were supported by suggestive evidence with random effects *p*-values smaller than 0.001 and more than 1000 participants in the relevant meta-analyses. None of these meta-analyses could reach the higher categories of evidence for a combination of reasons. Only 2 out of the 12 meta-analyses had *P* < 10^−6^, the largest study in the meta-analysis was not statistically significant in 7 out of 12, prediction intervals included almost always the null value (8 out of 12), and there was potential for small study effects (3 out of 12) and excess significance bias (4 out of 12). Finally, 44 associations were supported by weak evidence reporting just nominally statistically significant (*P* < 0.05) random effects calculations.

**Table 2:** Grading of the evidence for the meta-analyses of RCTs investigating the effectiveness of various psychological interventions for pain reduction

Author, Year	Intervention Group	Control group	Type of pain	Total N	Largest Study^a, b^	Summary random effects (95% CI)^a, c^	Random *P*-value ^d^	95% Prediction interval	Egger’s *P*-value^a^	I^2^ (%)	Studies	Excess significance^e^	
												O/E^g^	*P*-value^h^
Associations supported by strong evidence													
None of the associations studied was supported by strong evidence													
Associations supported by highly suggestive evidence													
None of the associations studied was supported by highly suggestive evidence													
Associations supported by suggestive evidence													
Bernardy K, 2013	CBT	AC/AtC/EDU/TAU/Support	Fibromyalgia, end of treatment	1150	−0.62 (−0.89, −0.34)	−0.30 (−0.45, −0.15)	7.4E-05	−0.69, 0.09	0.37	30	18	4/17.17	NP
Birnie K, 2014	Distraction	NR	Needle-related (children, adolescents), self-reported	2472	0.09 (−0.08, 0.27)	−0.44 (−0.67, −0.21)	2.0E-04	−1.53, 0.65	0.13	86	24	7/3.4	0.07
Dixon K, 2007	CBT/Stress management/HYP	NR	Arthritis	2303	−0.15 (−0.28, −0.02)	−0.20 (−0.30, −0.10)	8.5E-05	−0.37, −0.02	0.02	9	20	5/5.03	NP
Du S, 2011	ASMP/Self-management	WL/ UC/ Conventional/No treatment	Chronic musculoskeletal, 6 m	1018	−0.27 (−0.42, −0.12)	−0.29 (−0.42, −0.16)	6.0E-06	−1.11, 0.53	0.25	0	3	3/2.78	1.00
Du S, 2011	ASMP/Self-management	WL/ UC/ Conventional/No treatment	Chronic musculoskeletal, 4 m	2968	−0.35 (−0.49, −0.20)	−0.23 (−0.36, −0.11)	2.9E-04	−0.59, 0.13	0.73	59	8	4/7.94	NP
Eccleston C, 2014	Psychological therapies (Internet-delivered)	AC/TAU/WL	Chronic (Non-HA), post-treatment	1785	−0.20 (−0.36, −0.05)	−0.37 (−0.59, −0.15)	9.9E-04	−1.12, 0.38	0.42	77	11	5/5.96	NP
Henrich J, 2015	Psychological therapies	Control	Irritable bowel syndrome	2245	0.05 (−0.19, 0.28)	0.40 (0.30, 0.51)	3.3E-14	0.09, 0.72	0.01	26	32	9/2.22	<0.001
Johannsen M, 2013	EDU/RIMH/SGT	WL/ St. Care/ NR	Breast cancer patients/survivors	1500	0.09 (−0.14, 0.31)	0.34 (0.18, 0.50)	3.3E-05	−0.15, 0.82	0.06	50	15	6/2.24	0.02
Knittle K, 2010	Self-regulation	WL/St. Care/No Intervention	Rheumatoid Arthritis	1316	0.13 (−0.16, 0.41)	0.18 (0.07, 0.29)	8.9E-04	0.07, 0.30	0.53	0	22	1/3.73	NP
Kroon FP, 2014	SMP	UC/WL/No treatment	Osteoarthritis, IT	2271	−0.29 (−0.51, −0.07)	−0.17 (−0.26, −0.08)	1.6E-04	−0.27, −0.07	0.12	0	13	2/10.85	NP
Macea DD, 2010	Web-based CBT interventions	Control	Chronic pain	2958	0.28 (0.13, 0.42)	0.29 (0.15, 0.43)	6.3E-05	−0.07, 0.64	0.11	45	11	3/6.99	NP
Sheinfeld Gorin S, 2012	Psychological intervention	Control	Cancer Pain severity	4270	0.14 (−0.08, 0.36)	0.34 (0.23, 0.46)	7.2E-09	−0.21, 0.89	0.67	60	38	17/9.63	0.01
Associations supported by weak evidence													
Adachi T, 2013	Hypnosis	St. Care	Chronic, post-intervention	46	0.64 (−0.27, 1.55)	1.10 (0.17, 2.02)	0.020	NA	NA	48	2	1/1.52	NP
Aqqarwal VR, 2011	Any psychosocial intervention	Usual treatment	Muscle palpation, >3 m	143	−1.11 (−1.63, −0.59) ^i^	−1.09 (−1.56, −0.61)^i^	7.0E-06	−4.15, 1.98	0.61	0	3	1/2.05	NP
Aqqarwal VR, 2011	CBT	Usual treatment	Orofacial, >3 m	383	−0.32 (−0.66, 0.01)	−0.25 (−0.46, −0.05)	0.014	−0.70, 0.19	0.91	0	4	1/3.11	NP
Aqqarwal VR, 2011	CBT + BFB	Usual treatment	Orofacial, >3 m	196	−0.82 (−1.23, −0.41)	−0.46 (−0.92, 0.00)	0.049	−5.22, 4.30	0.47	53	3	1/3	NP
Aqqarwal VR, 2011	Hypnosis	REL	Orofacial, ≤3 m	81	−1.90 (−3.37, −0.43) ^i^	−1.84 (−3.26, −0.42)^i^	0.011	NA	NA	0	2	1/1.26	NP
Bernardy K, 2013	CBT	AC/AtC/EDU/TAU/Support	Fibromyalgia (self-efficacy), end of treatment	589	−0.93 (−1.32, −0.54)	−0.39 (−0.73, −0.06)	0.022	−1.50, 0.71	0.88	74	9	5/8.99	NP
Bernardy K, 2013	CBT	AC/AtC/EDU/TAU/Support	Fibromyalgia (self-efficacy), LT	494	−1.01 (−1.40, −0.61)	−0.52 (−1.04, 0.00)	0.049	−2.32, 1.28	0.82	86	8	3/8	NP
Bernardy K, 2013	Operant therapy	AC/EDU/TAU	Fibromyalgia (self-efficacy), LT	123	−1.16 (−1.73, −0.59)	−1.69 (−2.76, −0.62)	0.002	NA	NA	83	2	2/2	1.00
Bernardy K, 2013	CBT	AC/AtC/EDU/TAU/Support	Fibromyalgia, LT	770	−0.37 (−0.74, 0.00)	−0.28 (−0.43, −0.14)	1.3E-04	−0.47, −0.10	0.64	2	13	3/9.36	NP
Bernardy K, 2013	Operant therapy	AC/EDU/TAU	Fibromyalgia, LT	123	−0.76 (−1.31, −0.21)	−1.27 (−2.30, −0.24)	0.015	NA	NA	83	2	2/2	1.00
Damen L, 2006	REL + CBT	Attention placebo	HA Post-treatment	69	0.33 (−0.14, 0.79)	0.39 (0.01, 0.77)	0.045	NA	NA	0	2	0/1.21	NP
Du S, 2011	ASMP/Self-management	WL/ UC/ Conventional/No treatment	Chronic musculoskeletal, 12 m	1570	−0.05 (−0.19, 0.08)	−0.13 (−0.24, −0.03)	0.008	−0.30, 0.03	0.17	0	5	1/0.71	0.53
Eccleston C, 2014	Psychological therapies	Control	Chronic and recurrent HA (children, adolescents), follow-up	251	0.12 (0.02, 0.23)	0.49 (0.08, 0.90)	0.019	−0.76, 1.75	0.00	60	5	2/1.4	0.62
Eccleston C, 2014	Psychological therapies	Control	Chronic and recurrent HA (children, adolescents), post-treatment	714	0.32 (0.13, 0.52)	0.44 (0.28, 0.60)	1.2E-07	0.08, 0.80	0.00	25	15	7/7.46	NP
Eccleston C, 2014	Psychological therapies	Control	Chronic and recurrent non-HA (children, adolescents), post-treatment	852	0.21 (−0.10, 0.51)	−0.57 (−0.86, −0.27)	2.0E-04	−1.63, 0.50	0.00	75	13	5/4.58	0.78
Eccleston C, 2014	Psychological therapies (Internet-delivered)	AC/TAU/WL	Chronic HA, post-treatment	131	0.99 (0.35, 1.64)	1.10 (0.54, 1.65)	1.0E-04	NA	NA	0	2	2/1.94	1.00
Fisher E, 2014	CBT/BFB/REL/HYP	WL/EDU/St. Care/Self-monitoring	Chronic (excluding HA)	672	0.21 (−0.10, 0.51)	−0.60 (−0.91, −0.29)	1.7E-04	−1.63, 0.44	0.00	71	11	4/3.65	0.76
Fisher E, 2014	CBT/BFB/REL/HYP	WL/EDU/St. Care/Self-monitoring	Headache	748	0.25 (−0.03, 0.54)	0.50 (0.34, 0.66)	3.9E-10	0.18, 0.83	0.00	16	18	7/6.65	1.00
Guzman J, 2002	Intensive (>100 h) daily MBPSR with functional restoration	NR	Low back, 3-4 m	165	−0.45 (−0.86, −0.04)	−0.57 (−0.88, −0.26)	3.5E-04	NA	NA	0	2	2/1.94	1.00
Henschke N, 2011	CBT	WL	Chronic low back, ST	239	−0.54 (−0.93, −0.15)	−0.60 (−0.97, −0.23)	0.002	−1.66, 0.46	0.34	43	5	2/4.12	NP
Henschke N, 2011	Operant therapy	WL	Chronic low back, ST	153	−0.63 (−1.12, −0.13)	−0.43 (−0.75, −0.11)	0.009	−2.52, 1.66	0.32	0	3	1/2.95	NP
Henschke N, 2011	Respondent therapy (EMG BFB)	WL	Chronic low back, ST	64	−1.19 (−2.01, −0.37)	−0.80 (−1.32, −0.28)	0.002	−4.17, 2.56	0.76	0	3	1/2.99	NP
Henschke N, 2011	Respondent therapy (progressive REL)	WL	Chronic low back, ST	74	−10.20 (−23.95, 3.55) ^i^	−19.77 (−34.34, −5.20)^i^	0.008	−175.53, 135.98	0.53	57	3	1/1.94	NP
Kamper SJ, 2014	MBR	UC	Chronic low back, IT	740	−0.24 (−0.50, 0.03)	−0.60 (−0.85, −0.34)	5.1E-06	−1.37, 0.18	0.01	63	6	5/3.95	0.67
Kamper SJ, 2014	MBR	Physical treatment	Chronic low back, IT	531	−0.04 (−0.40, 0.32)	−0.28 (−0.54, −0.01)	0.039	−1.01, 0.45	0.17	51	9	2/0.54	0.10
Kamper SJ, 2014	MBR	UC	Chronic low back, LT	821	−0.32 (−0.60, −0.04)	−0.21 (−0.37, −0.04)	0.013	−0.57, 0.15	0.68	26	7	2/5.94	NP
Kamper SJ, 2014	MBR	UC	Chronic low back, ST	879	−0.20 (−0.46, 0.05)	−0.55 (−0.83, −0.27)	1.0E-04	−1.44, 0.33	0.29	72	9	5/3.94	0.52
Kamper SJ, 2014	MBR	WL	Chronic low back, ST	213	−0.45 (−0.84, −0.06)	−0.73 (−1.22, −0.24)	0.003	−6.10, 4.64	0.78	63	3	2/2.66	NP
Kamper SJ, 2014	MBR	Physical treatment	Chronic low back, ST	1661	−0.15 (−0.36, 0.05)	−0.30 (−0.54, −0.06)	0.015	−1.15, 0.55	0.62	80	12	3/4.53	NP
Kisely SR, 2015	Psychological intervention	No psychological intervention	Chest (frequency), ≤3 m	294	−0.09 (−0.57, 0.39) ^i^	−2.26 (−4.41, −0.11)^i^	0.039	−8.95, 4.42	0.29	94	7	4/0.45	<0.001
Kisely SR, 2015	Psychological intervention	No psychological intervention	Chest, ≤3 m	172	−0.21 (−0.34, −0.06)	−0.20 (−0.35, −0.05)	0.008	−1.78, 1.38	0.75	58	3	2/2.98	NP
Kisely SR, 2015	Psychological intervention	No psychological intervention	Chest, 3-12 m	111	−0.29 (−0.49, −0.09)	−0.30 (−0.44, −0.15)	6.1E-05	NA	NA	0	2	2/2	1.00
Kroon FP, 2014	SMP	UC/WL/No treatment	Osteoarthritis, ST	755	−0.32 (−0.61, −0.03)	−0.26 (−0.41, −0.11)	8.2E-04	−0.47, −0.04	0.89	0	6	2/5.09	NP
Kroon FP, 2014	SMP	Control	Osteoarthritis, IT	574	−0.22 (−0.45, 0.01)	−0.26 (−0.43, −0.09)	0.003	−1.38, 0.86	0.27	0	3	0/2.16	NP
Lakhan S, 2013	MBT/MBCT	EDU/WL/Support	Irritable bowel syndrome	160	−0.64 (−1.08, −0.20)	−0.59 (−0.91, −0.27)	2.6E-04	NA	NA	0	2	2/2	1.00
Mustafa M, 2013	Supportive/expressive group therapy	Usual treatment	Metastatic breast cancer	279	−0.75 (−1.36, −0.14) ^i^	−0.58 (−0.99, −0.17)^i^	0.005	−3.21, 2.05	0.90	0	3	1/2.94	NP
Peerdeman K, 2016	Imagery	Control/No treatment	Pain relief	301	0.20 (−0.17, 0.56)	0.24 (0.01, 0.46)	0.039	−0.26, 0.73	0.28	0	4	0/1.6	NP
Peerdeman K, 2016	Verbal suggestion	Control/No treatment	Pain relief	383	0.24 (0.06, 0.41)	0.31 (0.16, 0.46)	6.4E-05	−0.02, 0.64	0.08	0	4	2/2.3	NP
Peerdeman K, 2016	Verbal suggestion/Imagery	Control/No treatment	Affective pain	169	0.16 (−0.27, 0.60)	0.34 (0.07, 0.61)	0.013	−1.41, 2.09	0.70	0	3	1/0.78	1.00
Roldan-Barraza C, 2014	Psychosocial Intervention/Psychosocial Intervention + Usual Treatment	Tailored Usual Treatment	MTMD (self-reported), LT	403	0.80 (0.14, 1.46)	0.66 (0.23, 1.09)	0.003	−2.13, 3.45	0.54	0	3	2/2.94	NP
Theadom A, 2015	Psychological therapies	UC	Fibromyalgia, 6 m	371	−0.38 (−0.75, −0.02)	−0.52 (−0.76, −0.29)	1.5E-05	−1.06, 0.02	0.15	20	5	2/4.12	NP
Theadom A, 2015	Psychological therapies	UC	Fibromyalgia, post-intervention	453	−0.23 (−0.60, 0.15)	−0.33 (−0.52, −0.15)	4.8E-04	−0.56, −0.11	0.90	0	9	2/3.1	NP
Uman LS, 2013	Hypnosis	Control	Needle-related (children, adolescents), self-reported	176	0.09 (−0.57, 0.74)	−1.4 (−2.32, −0.47)	0.003	−4.81, 2.01	0.41	85	5	4/0.37	<0.001
Williams AC, 2012	Cognitive behavioural	TAU	Chronic (excl. HA), post-treatment	1148	−0.53 (−0.87, −0.19)	−0.21 (−0.37, −0.05)	0.010	−0.72, 0.29	0.02	45	16	4/15.61	NP

*Abbreviations*: *AC* Active control, *AtC* Attention control, *ASMP* Arthritis Self-Management Program, *BFB* Biofeedback, *CBT* Cognitive behavioural therapy, *EDU* Education, *EMG BFB* Electromyographic biofeedback, *HA* Headache, *HYP* Hypnotherapy, *IM* Imagination, *IT* Intermediate term, *LT* Long term, *m* Months, *MBT* Mindfulness-based therapy, *MBCT* Mindfulness-based cognitive therapy, *MBPSR* Multidisciplinary bio-psychosocial rehabilitation programs, *MTMD* Myofascial Temporomandibular Disorder, *MBR* Multidisciplinary biopsychological rehabilitation, *NA* Not applicable, because only two studies were available, *NP* Not pertinent, because the expected number of statistically significant studies is larger than the observed, *NR* Not reported, *REL* Relaxation, *RIMH* Relaxation, guided imagery, meditation or hypnosis, *SGT* Supportive group therapy, *SMP* Self-management education programmes, *ST* Short term, *St. Care* Standard care, *TAU* Treated as usual, *UC* Usual Care, *WL* Waiting list

^a^All summary point estimates on this table were indicative of pain reduction comparing the intervention to the control group. However, the original meta-analyses reported both positive and negative effects as observed on this table because they used different outcome metrics (e.g., pain reduction or difference in pain levels)

^b^On these comparisons MD is reported, instead of SMD

^c^Summary effect and 95% confidence interval of largest study (smallest standard error) in each meta-analysis

^d^Random effects refer to summary effect (95% CI) using the random-effects model

^e^ *P* value of summary random effects estimate

^f^ *P*-value from the Egger’s regression asymmetry test

^g^Expected number of statistically significant studies using the point estimate of the largest study (smallest standard error) as the plausible effect size

^h^Observed/Expected number of statistically significant studies

^i^ *P* value of the excess statistical significance test. All statistical tests were two-sided

When in a sensitivity analysis, we altered the threshold of total population size to 500 instead of 1000 participants, seven associations (osteoarthritis, headache; chronic low back in two different time points; fibromyalgia in long term; chronic non-headache; chronic and recurrent non-headache in children and adolescents) were upgraded from weak to suggestive evidence and one (chronic and recurrent headache in children and adolescents) was upgraded from weak to highly suggestive evidence. When we also included non-RCT evidence in our appraisal, 13 and 51 associations were supported by suggestive and weak evidence, respectively (Additional file 1: Table S5). The evidence grading across all studies compared to the grading of the proposed associations using only randomized evidence did not change with the exception of biofeedback versus control on post-treatment chronic back pain and verbal suggestion on pain relief, which were supported by highly suggestive evidence in studies of unclear design.

### Quality of the included studies

Based on the AMSTAR quality assessment tool (Additional file 1: Table S6), the quality of the included meta-analyses ranged widely, from 2 to 11 points, with a median of 7 points. Most of the included meta-analyses had high (16 of 38; 42%), or moderate (*n* = 16; 42%) quality and only 6 (16%) meta-analyses had low quality. To further evaluate the potential existence of bias in this evidence base, we collected and summarized on Additional file 1: Table S7 the quality assessment scores that were originally included in the evaluated meta-analyses. Briefly, most meta-analyses included on average primary studies of low to moderate quality.

## Discussion

In the present large-scale umbrella review, we examined the strength of the evidence and extent of potential biases in 150 published meta-analyses of psychological interventions for pain reduction. None of the 150 associations was supported by either strong or highly suggestive evidence. Only 12 associations from the 141 RCT-only meta-analyses were supported by suggestive evidence indicating reductions in pain from breast cancer, arthritis, rheumatoid arthritis, osteoarthritis, chronic musculoskeletal pain (in two different time points), fibromyalgia, self-reported needle-related pain in children and adolescents, irritable bowel syndrome pain, chronic pain, chronic non-headache pain, and cancer pain severity comparing different psychological interventions to standard care.

Of the 12 associations that were supported by suggestive evidence, six were related to musculoskeletal conditions. Specifically, evidence suggested that the Arthritis Self-Management Program, a program of interventions that aim to increase the individual’s ability to manage pain, had a statistically significant effect in lowering chronic musculoskeletal pain after four (SMD, −0.23; 95% CI, −0.36 to −0.11) or 6 months (SMD, −0.29; 95% CI, −0.42 to −0.16) compared to usual care. There was only weak evidence of Arthritis Self-Management Program lowering chronic musculoskeletal pain after 1 year of intervention, and the magnitude of the effect was smaller (SMD, −0.13; 95% CI: -0.24 to −0.03) indicating that while such interventions are potentially effective in the short-term, the effect seems to wear off with time. Suggestive evidence supported the effect of psychological treatments, such as cognitive-behavioural therapy, hypnosis or stress management, in lowering arthritis pain (SMD, −0.2; 95% CI: -0.3 to −0.1). The evidence was suggestive also for the effect of self-regulation on pain reduction in patients with rheumatoid arthritis (SMD, 0.18; 95% CI: 0.07 to 0.29) compared to standard-care and for self-management programs on osteoarthritis pain reduction (SMD, −0.17; 95% CI: -0.26 to −0.08). Finally, the same was true for fibromyalgia (SMD, −0.30; 95% CI, −0.45 to −0.15). The remaining six associations that were supported by suggestive evidence regarded cancer pain severity (SMD, 0.34; 95% CI: 0.23 to 0.46), pain in breast cancer patients (SMD, 0.34; 95% CI: 0.18 to 0.50), self-reported needle-related pain in children and adolescents (SMD, −0.44; 95% CI: -0.67 to −0.21), irritable bowel syndrome (SMD, 0.40; 95% CI: 0.30 to 0.51), chronic non-headache pain (SMD, −0.37; 95% CI: -0.59 to −0.15), and chronic pain (SMD, 0.29; 95% CI: 0.15 to 0.43). Although the latter associations were statistically significant at *P* < 10^−3^ and the evidence was supported by an adequate sample size in the relevant meta-analyses (>1000 participants), they could not reach the strong and highly suggestive categories of evidence for a combination of reasons relevant to evidence strength (*P* < 10^−6^) and validity, as prediction intervals included almost always the null value and there was potential for small study effects and excess significance bias.

Our results come in discordance with the generally strong belief in the literature that psychological therapies are universally effective on a variety of pain conditions [76–78]. However, this belief is mainly established based on a limited number of small primary studies, and future larger studies are warranted. Notably, the median number of individuals in the intervention and control groups in each individual study included in our systematic evaluation was only 33 and 28 respectively, whereas the median number of studies included in each meta-analysis was only three. Our evaluation revealed that the reported effectiveness is usually overstated in the existing studies. The nominally statistically significant associations between psychological interventions and pain were confirmed in less than half of the examined meta-analyses. In addition, the random effects estimates were statistically significant in only 20% of the meta-analyses, when a *P*-value threshold of 0.001 was applied. Furthermore, in only nine meta-analyses the prediction interval excluded the null value, thus suggesting that only 6% of future studies are expected to demonstrate substantial “positive” (i.e. not null) associations between psychological interventions and pain treatment.

Regarding the validity of the examined associations, the effect of the largest study in each meta-analysis, which is expected to provide the most stable and valid estimate, was nominally statistically significant in only 29% of the cases and the effect size was of small magnitude and often more conservative than the summary effect estimate. Heterogeneity was high or very high (I^2^ > 50%) in 42% of the meta-analyses. The evidence for presence of small study effects or excess significance bias was low overall, but the existence of biases cannot be ruled out based only on a negative and potentially underpowered statistical test in meta-analyses with few primary studies. A combination of different forms of biases might still be affecting the results. One such is the selective reporting of “positive” versus “negative” findings. In various areas of clinical investigation “negative” findings are of “limited impact” and, therefore, remain often unpublished. Statistical significance testing should not be used in the future as a criterion for publication. Moreover, one cannot exclude the possibility of questionable research practices, such as selective reporting of study methods and results, *p*-value fishing, or deciding to collect more or stop collecting data only after looking whether the results are statistically significant, which have been shown to constitute common research practices [15, 79–81]. Most of the included meta-analyses had a moderate and high quality rating based on the AMSTAR quality assessment tool. However, the herein included meta-analyses evaluated the quality of their primary studies as low to moderate with only a few exceptions of high quality studies.

Pain is a challenging clinical entity to assess due to its multifaceted and subjective nature. In our approach, we assessed pain reduction as an outcome of interest. The pain management literature includes many more outcomes including, but not limited to, measures of function, quality of life, depression and perception of coping abilities, which lie beyond the scope of the present work. Nevertheless, the selection of valid outcome measures for pain and pain-related disability is of great importance due to its close relationship to treatment efficacy replication. Moreover, in pain-related clinical trials, there is generally a lack of standardization both in the pain-related outcome measurement and in pain-related outcome reporting, hampering efforts to synthesize evidence [82]. Even, for the pain reduction assessment per se, there are a number of parameters that can contribute to the observed heterogeneity and/or affect the level of bias operating in the field; statistical versus clinical significance and the usual lack of minimal important difference metrics, daily home data collection challenges, questionnaire and scale structure variations, length of follow-up and appropriateness thereof. The validity and feasibility of objective pain measurements are all attributes of the study design that affect the validity of the evidence base and jeopardize its translational potential.

A crisis of confidence in psychological science has recently emerged [83], following a series of revelations of questionable research practices and presence of bias coupled with reluctance to publish study protocols and conduct replication studies [14, 15, 80]. Psychotherapies have been questioned as effective approaches to reduce mental suffering in many conditions [84, 85], such as depression. There are few studies investigating potential biases in the reported associations of psychological interventions for pain management [86], although such interventions are widely used in clinical practice. A further strength of our study was that the main analysis used only evidence from randomized controlled trials, which are considered the gold standard for evidence. Some limitations should be also acknowledged in our work. Excess statistical significance and asymmetry tests offer hints of bias, not definitive proof thereof, but our estimates are likely to be conservative as a negative test result does not exclude the potential for bias.

## Conclusions

In conclusion, the present findings support that the effectiveness of psychological treatments for pain management is overstated and the supporting empirical evidence is weak. The present findings combined with the fact that psychological intervention trials are still at an early research stage and fall short compared to drug trials [87] underline the necessity for larger and better-conducted RCTs [85] Future research should further focus on building networks involving all stakeholder groups to achieve consensus and develop guidance on best practices for assessing and reporting pain outcomes [88, 89]. The use of standardized definitions and protocols for exposures, outcomes, and statistical analyses may diminish the threat of biases and improve the reliability of this important literature.

## Additional file

Additional file 1 Table S1. Description and summary effects of the 150 meta-analyses investigating the effectiveness of various psychological interventions for pain reduction. **Table S2.** Evaluation of bias and heterogeneity in the 150 meta-analyses investigating the effectiveness of various psychological interventions for pain reduction. **Table S3.** Description and summary effects of the 141 meta-analyses of RCTs investigating the effectiveness of various psychological interventions for pain reduction. **Table S4.** Evaluation of bias and heterogeneity in the 141 meta-analyses of RCTs investigating the effectiveness of various psychological interventions for pain reduction. **Table S5.** Grading of the evidence for all the meta-analyses investigating the effectiveness of various psychological interventions for pain reduction. **Table S6.** AMSTAR quality assessment of the 38 included meta-analysis papers. **Table S7.** Summary of the quality assessment scores performed in the 38 original meta-analysis papers. (DOCX 181 kb)

## Abbreviations

CI: Confidence interval
MD: Mean difference
RCT: Randomized controlled trial
SMD: Standardised mean difference
