# Supplementary material for: An umbrella review of the literature on the effectiveness of psychological interventions for pain reduction
Source: BMC Psychol. 2017 Aug 31;5:31. doi: 10.1186/s40359-017-0200-5 (PMC5580223; doi:10.1186/s40359-017-0200-5)
Supplement: Additional file 1 Table S1. — Description and summary effects of the 150 meta-analyses investigating the effectiveness of various psychological interventions for pain reduction. Table S2. Evaluation of bias and heterogeneity in the 150 meta-analyses investigating the effectiveness of various psychological interventions for pain reduction. Table S3. Description and summary effects of the 141 meta-analyses of RCTs investigating the effectiveness of various psychological interventions for pain reduction. Table S4. Evaluation of bias and heterogeneity in the 141 meta-analyses of RCTs investigating the effectiveness of various psychological interventions for pain reduction. Table S5. Grading of the evidence for all the meta-analyses investigating the effectiveness of various psychological interventions for pain reduction. Table S6. AMSTAR quality assessment of the 38 included meta-analysis papers. Table S7. Summary of the quality assessment scores performed in the 38 original meta-analysis papers. (DOCX 181 kb) [file 40359_2017_200_MOESM1_ESM.docx]

**Table S1:** Description and summary effects of the 150 meta-analyses investigating the effectiveness of various psychological interventions for pain reduction.

| **Author, Year** | **Intervention Group** | **Control group** | **Type of pain** | **Control/ Intervention N** | **Summary effect (95% Confidence interval) ^a^** | | | **Fixed  P-value ^e^** | **Random P-value ^f^** | **95% Prediction interval ^g^** |
| --- | --- | --- | --- | --- | --- | --- | --- | --- | --- | --- |
|  |  |  |  |  | **Fixed Effects ^b^** | **Random effects ^c^** | **Largest Study ^d^** |  |  |  |
| Adachi T, 2013 | Hypnosis | St. Care | Chronic, post-intervention | 79/84 | 0.43 (0.11, 0.76) | 0.60 (0.03, 1.17) | 0.19 (-0.23, 0.60) | 0.009 | 0.039 | -1.65, 2.85 |
| Adachi T, 2013 | Hypnosis | Other psychological treatment | Chronic, post-intervention | 246/259 | 0.00 (-0.18, 0.17) | 0.04 (-0.22, 0.30) | -0.46 (-0.9, -0.02) | 0.963 | 0.786 | -0.74, 0.81 |
| Adachi T, 2013 | Hypnosis | Other psychological treatment | Chronic, follow-up | 130/151 | -0.12 (-0.35, 0.12) | -0.12 (-0.38, 0.14) | -0.27 (-0.73, 0.20) | 0.336 | 0.382 | -0.61, 0.37 |
| Aqqarwal VR, 2011 | Any psychosocial intervention | Usual treatment | Muscle palpation, >3m | 69/74 | -1.09 (-1.56, -0.61) ^h^ | -1.09 (-1.56, -0.61) ^h^ | -1.11 (-1.63, -0.59) ^h^ | 7.0E-06 | 7.0E-06 | -4.15, 1.98 |
| Aqqarwal VR, 2011 | CBT | Usual treatment | Orofacial, >3m | 187/196 | -0.25 (-0.46, -0.05) | -0.25 (-0.46, -0.05) | -0.32 (-0.66, 0.01) | 0.014 | 0.014 | -0.70, 0.19 |
| Aqqarwal VR, 2011 | CBT+Biofeedback | Usual treatment | Orofacial, >3m | 81/115 | -0.52 (-0.82, -0.23) | -0.46 (-0.92, 0.00) | -0.82 (-1.23, -0.41) | 5.5E-04 | 0.049 | -5.22, 4.30 |
| Aqqarwal VR, 2011 | Biofeedback | Usual treatment | Orofacial, ≤3m | 15/30 | -0.40 (-1.06, 0.25) | -0.46 (-1.3, 0.39) | -0.11 (-0.91, 0.70) | 0.227 | 0.289 | NA |
| Aqqarwal VR, 2011 | CBT | Usual treatment | Orofacial, ≤3m | 203/208 | 0.03 (-0.16, 0.23) | 0.03 (-0.16, 0.23) | 0.24 (-0.09, 0.57) | 0.749 | 0.749 | -0.40, 0.46 |
| Aqqarwal VR, 2011 | CBT+Biofeedback | Usual treatment | Orofacial, ≤3m | 36/54 | 0.46 (0.02, 0.90) | 0.40 (-0.23, 1.03) | 0.66 (0.13, 1.19) | 0.042 | 0.214 | NA |
| Aqqarwal VR, 2011 | HYP | REL | Orofacial, ≤3m | 39/42 | -1.84 (-3.26, -0.42) ^h^ | -1.84 (-3.26, -0.42) ^h^ | -1.90 (-3.37, -0.43) ^h^ | 0.011 | 0.011 | NA |
| Bawa F, 2015 | Mindfulness | AC | Chronic pain intensity | 203/146 | 0.09 (-0.13, 0.31) | 0.09 (-0.13, 0.31) | 0.1 (-0.29, 0.49) | 0.420 | 0.420 | -0.27, 0.45 |
| Bawa F, 2015 | Mindfulness | Inactive Control | Chronic pain intensity | 49/55 | 0.38 (-0.01, 0.78) | 0.38 (-0.01, 0.78) | 0.23 (-0.42, 0.88) | 0.055 | 0.055 | -0.48, 1.25 |
| Bernardy K, 2013 | CBT | AC/AtC/EDU/TAU/Support | Fibromyalgia (self-efficacy), end of treatment | 275/314 | -0.38 (-0.55, -0.22) | -0.39 (-0.73, -0.06) | -0.93 (-1.32, -0.54) | 6.2E-06 | 0.022 | -1.50, 0.71 |
| Bernardy K, 2013 | Operant therapy | AC/EDU/TAU | Fibromyalgia (self-efficacy), end of treatment | 40/83 | -0.99 (-1.41, -0.58) | -1.18 (-3.02, 0.65) | -0.26 (-0.80, 0.27) | 2.9E-06 | 0.205 | NA |
| Bernardy K, 2013 | CBT | AC/AtC/EDU/TAU/Support | Fibromyalgia (self-efficacy), LT | 229/265 | -0.49 (-0.68, -0.30) | -0.52 (-1.04, 0.00) | -1.01 (-1.40, -0.61) | 2.4E-07 | 0.049 | -2.32, 1.28 |
| Bernardy K, 2013 | Operant therapy | AC/EDU/TAU | Fibromyalgia (self-efficacy), LT | 40/83 | -1.61 (-2.05, -1.17) | -1.69 (-2.76, -0.62) | -1.16 (-1.73, -0.59) | 5.1E-13 | 0.002 | NA |
| Bernardy K, 2013 | CBT | AC/AtC/EDU/TAU/Support | Fibromyalgia, end of treatment | 547/603 | -0.32 (-0.44, -0.20) | -0.30 (-0.45, -0.15) | -0.62 (-0.89, -0.34) | 9.9E-08 | 7.4E-05 | -0.69, 0.09 |
| Bernardy K, 2013 | Operant therapy | AC/AtC/EDU/Support | Fibromyalgia, end of treatment | 40/83 | -0.53 (-0.93, -0.12) | -0.66 (-2.56, 1.24) | 0.30 (-0.24, 0.83) | 0.011 | 0.494 | NA |
| Bernardy K, 2013 | Self-management | AC/TAU | Fibromyalgia, end of treatment | 94/86 | -0.10 (-0.39, 0.20) | 0.02 (-0.80, 0.84) | -0.38 (-0.75, -0.02) | 0.528 | 0.961 | NA |
| Bernardy K, 2013 | CBT | AC/AtC/EDU/TAU/Support | Fibromyalgia, LT | 360/410 | -0.28 (-0.43, -0.14) | -0.28 (-0.43, -0.14) | -0.37 (-0.74, 0.00) | 1.0E-04 | 1.3E-04 | -0.47, -0.10 |
| Bernardy K, 2013 | Operant therapy | AC/EDU/TAU | Fibromyalgia, LT | 40/83 | -1.21 (-1.63, -0.80) | -1.27 (-2.30, -0.24) | -0.76 (-1.31, -0.21) | 9.3E-09 | 0.015 | NA |
| Bernardy K, 2011 | HYP | CBT/ TAU/ WL/ Attention placebo | Fibromyalgia | 80/98 | -1.10 (-1.44, -0.75) | -1.17 (-2.21, -0.13) | -1.52 (-2.17, -0.87) | 3.9E-10 | 0.028 | -4.83, 2.49 |
| Birnie K, 2014 | Distraction | NR | Needle-related (children, adolescents), self-reported | 1229/1243 | -0.32 (-0.41, -0.24) | -0.44 (-0.67, -0.21) | 0.09 (-0.08, 0.27) | 8.8E-15 | 2.0E-04 | -1.53, 0.65 |
| Champaneria, 2012 | Psychological intervention | No psychological intervention | Chronic pelvic, ≥6m | 76/63 | -1.57 (-2.53, -0.61) ^h^ | -1.70 (-6.1, 2.70) ^h^ | 0.54 (-0.78, 1.86) ^h^ | 0.001 | 0.449 | NA |
| Champaneria, 2012 | Psychological intervention | No psychological intervention | Chronic pelvic, 3m | 79/77 | -0.92 (-1.77, -0.07) ^h^ | -1.07 (-5.37, 3.22) ^h^ | 1.11 (-0.05, 2.27) ^h^ | 0.035 | 0.624 | NA |
| Damen L, 2006 | Biofeedback | Control | HA Post-treatment | 34/37 | 1.16 (0.25, 2.08) | 1.16 (0.25, 2.08) | 1.41 (-0.11, 2.93) | 0.012 | 0.012 | -4.75, 7.08 |
| Damen L, 2006 | REL + Biofeedback + CBT | WL | HA Post-treatment | 15/38 | 0.58 (0.02, 1.13) | 0.58 (0.02, 1.13) | 0.51 (-0.15, 1.16) | 0.042 | 0.042 | NA |
| Damen L, 2006 | REL + CBT | Attention placebo | HA Post-treatment | 34/35 | 0.39 (0.01, 0.77) | 0.39 (0.01, 0.77) | 0.33 (-0.14, 0.79) | 0.045 | 0.045 | NA |
| Damen L, 2006 | REL+Biofeedback | WL | HA Post-treatment | 24/20 | 0.36 (0.09, 0.63) | 0.73 (-0.35, 1.82) | 0.28 (0.00, 0.56) | 0.009 | 0.186 | NA |
| Dixon K, 2007 | CBT/ Stress management/ HYP | NR | Arthritis | 1136/1167 | -0.18 (-0.26, -0.10) | -0.20 (-0.30, -0.10) | -0.15 (-0.28, -0.02) | 2.0E-05 | 8.5E-05 | -0.37, -0.02 |
| Du S, 2011 | ASMP/ Self-management | WL/ UC/ Conventional/No treatment | Chronic musculoskeletal, 12m | 770/800 | -0.13 (-0.24, -0.03) | -0.13 (-0.24, -0.03) | -0.05 (-0.19, 0.08) | 0.008 | 0.008 | -0.30, 0.03 |
| Du S, 2011 | ASMP/ Self-management | WL/ UC/ Conventional/No treatment | Chronic musculoskeletal, 4m | 1279/1689 | -0.22 (-0.30, -0.15) | -0.23 (-0.36, -0.11) | -0.35 (-0.49, -0.20) | 4.1E-09 | 2.9E-04 | -0.59, 0.13 |
| Du S, 2011 | ASMP/ Self-management | WL/ UC/ Conventional/No treatment | Chronic musculoskeletal, 6m | 524/494 | -0.29 (-0.42, -0.16) | -0.29 (-0.42, -0.16) | -0.27 (-0.42, -0.12) | 6.0E-06 | 6.0E-06 | -1.11, 0.53 |
| Eccleston C, 2014 | Psychological therapies (Internet-delivered) | AC/TAU/WL | Chronic (Non-HA), follow-up | 617/585 | -0.29 (-0.41, -0.18) | -0.48 (-1.18, 0.22) | -0.14 (-0.29, 0.02) | 6.9E-07 | 0.181 | -3.83, 2.88 |
| Eccleston C, 2014 | Psychological therapies (Internet-delivered) | AC/TAU/WL | Chronic (Non-HA), post-treatment | 903/882 | -0.31 (-0.40, -0.22) | -0.37 (-0.59, -0.15) | -0.20 (-0.36, -0.05) | 9.4E-11 | 9.9E-04 | -1.12, 0.38 |
| Eccleston C, 2014 | Psychological therapies | Control | Chronic and recurrent HA (children, adolescents), follow-up | 111/140 | 0.16 (0.06, 0.27) | 0.49 (0.08, 0.90) | 0.12 (0.02, 0.23) | 0.002 | 0.019 | -0.76, 1.75 |
| Eccleston C, 2014 | Psychological therapies | Control | Chronic and recurrent HA (children, adolescents), post-treatment | 287/427 | 0.38 (0.26, 0.49) | 0.44 (0.28, 0.60) | 0.32 (0.13, 0.52) | 3.8E-10 | 1.2E-07 | 0.08, 0.80 |
| Eccleston C, 2014 | Psychological therapies | Control | Chronic and recurrent non-HA (children, adolescents), follow-up | 270/273 | -0.04 (-0.21, 0.13) | -0.11 (-0.41, 0.19) | 0.16 (-0.16, 0.47) | 0.644 | 0.469 | -0.98, 0.76 |
| Eccleston C, 2014 | Psychological therapies | Control | Chronic and recurrent non-HA (children, adolescents), post-treatment | 404/448 | -0.36 (-0.50, -0.22) | -0.57 (-0.86, -0.27) | 0.21 (-0.10, 0.51) | 3.4E-07 | 2.0E-04 | -1.63, 0.50 |
| Eccleston C, 2014 | Psychological therapies (Internet-delivered) | AC/TAU/WL | Chronic HA, post-treatment | 72/59 | 1.10 (0.54, 1.65) | 1.10 (0.54, 1.65) | 0.99 (0.35, 1.64) | 1.0E-04 | 1.0E-04 | NA |
| Fisher E, 2014 | CBT/ Biofeedback/ REL/ HYP | WL/ EDU/ St. Care/ Self-monitoring | Chronic (excluding HA) | 310/362 | -0.42 (-0.58, -0.26) | -0.60 (-0.91, -0.29) | 0.21 (-0.10, 0.51) | 1.7E-07 | 1.7E-04 | -1.63, 0.44 |
| Fisher E, 2014 | CBT/ Biofeedback/ REL/ HYP | WL/ EDU/ St. Care/ Self-monitoring | Headache | 289/459 | 0.45 (0.32, 0.59) | 0.50 (0.34, 0.66) | 0.25 (-0.03, 0.54) | 2.9E-11 | 3.9E-10 | 0.18, 0.83 |
| Flanagan E, 2015 | CBT | Medical treatment | General vaginal, post-treatment | 56/87 | 0.28 (-0.06, 0.62) | 0.29 (-0.12, 0.70) | 0.15 (-0.32, 0.63) | 0.105 | 0.166 | -3.37, 3.95 |
| Flanagan E, 2015 | CBT | Medical treatment | Pain on intercourse, 6m | 39/62 | -0.12 (-1.34, 1.09) | 0.00 (-1.63, 1.63) | -0.66 (-2.13, 0.81) | 0.844 | 0.999 | NA |
| Flanagan E, 2015 | CBT | Other psychological treatment | Pain on intercourse, follow-up | 42/41 | -0.46 (-0.90, -0.02) | -0.44 (-1.23, 0.36) | -0.83 (-1.43, -0.23) | 0.041 | 0.281 | NA |
| Flanagan E, 2015 | CBT | Medical treatment | Pain on intercourse, post-treatment | 44/66 | 0.68 (-0.46, 1.83) | 0.86 (-1.37, 3.09) | -0.21 (-1.68, 1.26) | 0.242 | 0.449 | NA |
| Flanagan E, 2015 | CBT | Other psychological treatment | Pain on intercourse, post-treatment | 67/81 | -0.06 (-0.39, 0.27) | -0.06 (-0.41, 0.3) | 0.02 (-0.50, 0.53) | 0.729 | 0.750 | -2.75, 2.63 |
| Flanagan E, 2015 | CBT + behavioural | WL | Pain on intercourse, post-treatment | 58/66 | -0.79 (-1.18, -0.40) | -0.81 (-2.61, 0.98) | 0.1 (-0.44, 0.64) | 6.3E-05 | 0.374 | NA |
| Glombiewski JA, 2010 | EDU/ CBT/ REL | CBT/ TAU/ WL/ Attention placebo | Fibromyalgia | 489/528 | 0.35 (0.29, 0.42) | 0.41 (0.29, 0.54) | 0.07 (-0.13, 0.26) | 5.6E-26 | 5.2E-11 | -0.09, 0.92 |
| Guzman J, 2002 | Less intensive (<30 h) once or twice weekly MBPSR | NR | Low back, 12m | 177/188 | 0.15 (-0.05, 0.36) | 0.13 (-0.25, 0.51) | 0.14 (-0.10, 0.38) | 0.149 | 0.496 | -3.85, 4.11 |
| Guzman J, 2002 | Other types of MBPSR | NR | Low back, 12m | 205/237 | 0.00 (-0.18, 0.19) | 0.00 (-0.18, 0.19) | -0.05 (-0.28, 0.18) | 0.982 | 0.982 | NA |
| Guzman J, 2002 | Less intensive (<30 h) once or twice weekly MBPSR | NR | Low back, 24-30m | 155/165 | 0.17 (-0.05, 0.39) | 0.17 (-0.05, 0.39) | 0.12 (-0.13, 0.36) | 0.141 | 0.141 | NA |
| Guzman J, 2002 | Intensive (>100h) daily MBPSR with functional restoration | NR | Low back, 24m | 77/90 | -0.39 (-0.70, -0.08) | -0.43 (-0.97, 0.10) | -0.18 (-0.57, 0.22) | 0.013 | 0.114 | NA |
| Guzman J, 2002 | Intensive (>100h) daily MBPSR with functional restoration | NR | Low back, 3-4m | 80/85 | -0.57 (-0.88, -0.26) | -0.57 (-0.88, -0.26) | -0.45 (-0.86, -0.04) | 3.5E-04 | 3.5E-04 | NA |
| Guzman J, 2002 | Less intensive (<30 h) once or twice weekly MBPSR | NR | Low back, 3-6m | 206/214 | -0.12 (-0.31, 0.08) | -0.07 (-0.50, 0.37) | -0.20 (-0.43, 0.03) | 0.231 | 0.758 | -1.81, 1.67 |
| Guzman J, 2002 | Intensive (>100h) daily MBPSR with functional restoration | NR | Low back, 60m | 71/83 | -0.21 (-0.53, 0.11) | -0.24 (-0.74, 0.26) | 0.00 (-0.42, 0.42) | 0.191 | 0.351 | NA |
| Guzman J, 2002 | Less intensive (<30 h) once or twice weekly MBPSR | NR | Low back, at treatment completion | 65/77 | -0.23 (-0.57, 0.11) | -0.22 (-0.89, 0.45) | -0.06 (-0.51, 0.39) | 0.182 | 0.529 | -7.77, 7.34 |
| Henrich J, 2015 | Psychological therapies | Control | Irritable bowel syndrome | 1038/1207 | 0.37 (0.28, 0.45) | 0.40 (0.30, 0.51) | 0.05 (-0.19, 0.28) | 1.5E-17 | 3.3E-14 | 0.09, 0.72 |
| Henschke N, 2011 | Behavioural treatment | UC | Chronic low back, IT | 155/164 | -4.29 (-9.28, 0.69) ^h^ | -4.29 (-9.28, 0.69) ^h^ | -5.00 (-10.56, 0.56) ^h^ | 0.091 | 0.091 | NA |
| Henschke N, 2011 | Behavioural treatment | Group exercise | Chronic low back, IT | 68/69 | 1.18 (-3.16, 5.53) ^h^ | 1.18 (-3.16, 5.53) ^h^ | 0.44 (-4.47, 5.35) ^h^ | 0.594 | 0.594 | NA |
| Henschke N, 2011 | Behavioural treatment + physiotherapy | Physiotherapy | Chronic low back, IT | 16/29 | -0.12 (-0.68, 0.44) ^h^ | -0.12 (-0.68, 0.44) ^h^ | -0.29 (-1.01, 0.44) ^h^ | 0.684 | 0.684 | NA |
| Henschke N, 2011 | CBT | Cognitive therapy | Chronic low back, IT | 19/25 | 0.14 (-0.51, 0.79) | -0.31 (-2.59, 1.98) | 0.81 (0.04, 1.59) | 0.675 | 0.793 | NA |
| Henschke N, 2011 | CBT | Operant therapy | Chronic low back, IT | 65/75 | -0.23 (-0.57, 0.11) | -0.23 (-0.57, 0.11) | -0.10 (-0.54, 0.34) | 0.182 | 0.182 | -2.44, 1.97 |
| Henschke N, 2011 | CBT | Respondent therapy | Chronic low back, IT | 32/30 | 0.44 (-0.07, 0.95) | 0.47 (-0.42, 1.35) | 0.03 (-0.66, 0.72) | 0.094 | 0.301 | NA |
| Henschke N, 2011 | Cognitive therapy | Operant therapy | Chronic low back, IT | 39/43 | 0.13 (-0.31, 0.57) | 0.35 (-0.64, 1.35) | 0.03 (-0.43, 0.49) | 0.572 | 0.485 | NA |
| Henschke N, 2011 | Behavioural treatment | Group exercise | Chronic low back, LT | 67/69 | 0.13 (-4.40, 4.67) ^h^ | 0.13 (-4.40, 4.67) ^h^ | -0.62 (-6.29, 5.05) ^h^ | 0.953 | 0.953 | NA |
| Henschke N, 2011 | CBT | Cognitive therapy | Chronic low back, LT | 18/30 | -0.08 (-0.72, 0.55) | -0.89 (-3.64, 1.86) | 0.44 (-0.26, 1.15) | 0.799 | 0.527 | NA |
| Henschke N, 2011 | CBT | Operant therapy | Chronic low back, LT | 67/73 | -0.31 (-0.65, 0.03) | -0.31 (-0.65, 0.03) | -0.24 (-0.69, 0.21) | 0.073 | 0.073 | -2.51, 1.89 |
| Henschke N, 2011 | Behavioural treatment | UC | Chronic low back, ST | 163/167 | -5.18 (-9.78, -0.57) ^h^ | -5.63 (-11.44, 0.17) ^h^ | -4.00 (-9.05, 1.05) ^h^ | 0.028 | 0.057 | NA |
| Henschke N, 2011 | Behavioural treatment | Group exercise | Chronic low back, ST | 73/73 | -2.31 (-6.33, 1.71) ^h^ | -2.31 (-6.33, 1.71) ^h^ | -3.50 (-8.38, 1.38) ^h^ | 0.260 | 0.260 | NA |
| Henschke N, 2011 | Behavioural treatment + physiotherapy | Physiotherapy | Chronic low back, ST | 18/41 | -0.20 (-0.65, 0.24) ^h^ | -0.13 (-1.01, 0.75) ^h^ | -0.55 (-1.12, 0.02) ^h^ | 0.373 | 0.776 | NA |
| Henschke N, 2011 | Behavioural treatment + physiotherapy | Inpatient rehabilitation | Chronic low back, ST | 214/191 | -0.15 (-0.34, 0.05) | -0.15 (-0.34, 0.05) | -0.17 (-0.37, 0.04) | 0.140 | 0.140 | NA |
| Henschke N, 2011 | CBT | WL | Chronic low back, ST | 110/129 | -0.56 (-0.82, -0.30) | -0.6 (-0.97, -0.23) | -0.54 (-0.93, -0.15) | 2.7E-05 | 0.002 | -1.66, 0.46 |
| Henschke N, 2011 | CBT | Cognitive therapy | Chronic low back, ST | 24/37 | -0.11 (-0.64, 0.42) | -0.24 (-1.35, 0.88) | 0.29 (-0.37, 0.94) | 0.681 | 0.675 | NA |
| Henschke N, 2011 | CBT | Operant therapy | Chronic low back, ST | 76/85 | -0.15 (-0.46, 0.16) | -0.15 (-0.46, 0.16) | -0.18 (-0.61, 0.25) | 0.349 | 0.349 | -2.18, 1.88 |
| Henschke N, 2011 | CBT | Respondent therapy | Chronic low back, ST | 47/50 | 0.10 (-0.30, 0.50) | 0.10 (-0.30, 0.50) | 0.25 (-0.39, 0.89) | 0.641 | 0.641 | -2.50, 2.69 |
| Henschke N, 2011 | Cognitive therapy | WL | Chronic low back, ST | 39/29 | -0.27 (-0.75, 0.22) | -0.27 (-0.79, 0.25) | -0.53 (-1.21, 0.16) | 0.282 | 0.314 | NA |
| Henschke N, 2011 | Cognitive therapy | Operant therapy | Chronic low back, ST | 44/49 | 0.14 (-0.27, 0.55) | 0.41 (-0.63, 1.46) | -0.02 (-0.46, 0.43) | 0.502 | 0.440 | NA |
| Henschke N, 2011 | Operant therapy | WL | Chronic low back, ST | 70/83 | -0.43 (-0.75, -0.11) | -0.43 (-0.75, -0.11) | -0.63 (-1.12, -0.13) | 0.009 | 0.009 | -2.52, 1.66 |
| Henschke N, 2011 | Respondent therapy (EMG Biofeedback) | WL | Chronic low back, ST | 30/34 | -0.80 (-1.32, -0.28) | -0.80 (-1.32, -0.28) | -1.19 (-2.01, -0.37) | 0.002 | 0.002 | -4.17, 2.56 |
| Henschke N, 2011 | Respondent therapy (progressive REL) | WL | Chronic low back, ST | 35/39 | -18.67 (-28.00, -9.33)^h^ | -19.77 (-34.34, -5.20)^h^ | -10.20 (-23.95, 3.55) ^h^ | 8.9E-05 | 0.008 | -175.53, 135.98 |
| Johannsen M, 2013 | EDU/ RIMH/ SGT | WL/ St. Care/ NR | Breast cancer patients/survivors | 755/1015 | 0.29 (0.19, 0.38) | 0.33 (0.19, 0.47) | 0.09 (-0.14, 0.31) | 7.1E-09 | 2.5E-06 | -0.08, 0.74 |
| Kamper SJ, 2014 | MBR | UC | Chronic low back, IT | 369/371 | -0.51 (-0.66, -0.36) | -0.60 (-0.85, -0.34) | -0.24 (-0.50, 0.03) | 1.7E-11 | 5.1E-06 | -1.37, 0.18 |
| Kamper SJ, 2014 | MBR | Physical treatment | Chronic low back, IT | 265/266 | -0.23 (-0.40, -0.05) | -0.28 (-0.54, -0.01) | -0.04 (-0.40, 0.32) | 0.011 | 0.039 | -1.01, 0.45 |
| Kamper SJ, 2014 | MBR | UC | Chronic low back, LT | 373/448 | -0.20 (-0.34, -0.07) | -0.21 (-0.37, -0.04) | -0.32 (-0.60, -0.04) | 0.004 | 0.013 | -0.57, 0.15 |
| Kamper SJ, 2014 | MBR | Physical treatment | Chronic low back, LT | 437/435 | -0.29 (-0.43, -0.15) | -0.51 (-1.04, 0.01) | -0.17 (-0.42, 0.08) | 3.8E-05 | 0.057 | -2.41, 1.39 |
| Kamper SJ, 2014 | MBR | Surgery | Chronic low back, LT | 188/197 | -0.23 (-0.43, -0.02) | -0.25 (-0.54, 0.04) | -0.12 (-0.37, 0.13) | 0.028 | 0.096 | NA |
| Kamper SJ, 2014 | MBR | UC | Chronic low back, ST | 446/433 | -0.47 (-0.60, -0.33) | -0.55 (-0.83, -0.27) | -0.20 (-0.46, 0.05) | 9.5E-12 | 1.0E-04 | -1.44, 0.33 |
| Kamper SJ, 2014 | MBR | Physical treatment | Chronic low back, ST | 851/810 | -0.25 (-0.35, -0.16) | -0.3 (-0.54, -0.06) | -0.15 (-0.36, 0.05) | 3.2E-07 | 0.015 | -1.15, 0.55 |
| Kamper SJ, 2014 | MBR | WL | Chronic low back, ST | 107/106 | -0.69 (-0.98, -0.41) | -0.73 (-1.22, -0.24) | -0.45 (-0.84, -0.06) | 1.2E-06 | 0.003 | -6.10, 4.64 |
| Kisely SR, 2015 | Psychological intervention | No psychological intervention | Chest (frequency), ≤3m | 140/154 | -1.21 (-1.58, -0.84) ^h^ | -2.26 (-4.41, -0.11) ^h^ | -0.09 (-0.57, 0.39) ^h^ | 1.9E-10 | 0.039 | -8.95, 4.42 |
| Kisely SR, 2015 | Psychological intervention | No psychological intervention | Chest (frequency), 3-12m | 80/84 | 0.03 (-0.41, 0.48) ^h^ | -0.81 (-2.36, 0.75) ^h^ | 0.21 (-0.27, 0.69) ^h^ | 0.877 | 0.309 | -7.28, 5.67 |
| Kisely SR, 2015 | Psychological intervention | No psychological intervention | Chest (severity), ≤3m | 86/94 | -0.91 (-1.55, -0.26) ^h^ | -4.64 (-12.18, 2.89) ^h^ | -0.85 (-1.50, -0.20) ^h^ | 0.006 | 0.227 | -34.88, 25.59 |
| Kisely SR, 2015 | Psychological intervention | No psychological intervention | Chest, ≤3m | 82/90 | -0.19 (-0.29, -0.10) | -0.20 (-0.35, -0.05) | -0.21 (-0.34, -0.06) | 5.5E-05 | 0.008 | -1.78, 1.38 |
| Kisely SR, 2015 | Psychological intervention | No psychological intervention | Chest, 3-12m | 61/50 | -0.30 (-0.44, -0.15) | -0.30 (-0.44, -0.15) | -0.29 (-0.49, -0.09) | 6.1E-05 | 6.1E-05 | NA |
| Knittle K, 2010 | Self-regulation | WL/ St. Care/ No Intervention | Rheumatoid Arthritis | 638/678 | 0.18 (0.07, 0.29) | 0.18 (0.07, 0.29) | 0.13 (-0.16, 0.41) | 8.9E-04 | 8.9E-04 | 0.07, 0.30 |
| Koranyi S, 2014 | Psychological intervention | Control | Acute pain after open heart surgery, IT | 210/203 | -0.01 (-0.19, 0.16) | -0.03 (-0.25, 0.19) | 0.16 (-0.11, 0.44) | 0.880 | 0.814 | -0.78, 0.72 |
| Koranyi S, 2014 | Psychological intervention | TAU | Acute pain after open heart surgery, IT | 150/143 | 0.09 (-0.11, 0.29) | 0.09 (-0.11, 0.29) | 0.16 (-0.11, 0.44) | 0.392 | 0.392 | -1.21, 1.39 |
| Koranyi S, 2014 | Psychological intervention | Control | Acute pain after open heart surgery, LT | 143/137 | 0.12 (-0.09, 0.33) | 0.12 (-0.09, 0.33) | 0.06 (-0.25, 0.37) | 0.251 | 0.251 | -1.24, 1.48 |
| Kroon FP, 2014 | SMP | UC/WL/No treatment | Osteoarthritis, ST | 282/473 | -0.26 (-0.41, -0.11) | -0.26 (-0.41, -0.11) | -0.32 (-0.61, -0.03) | 8.2E-04 | 8.2E-04 | -0.47, -0.04 |
| Kroon FP, 2014 | SMP | No SMP | Osteoarthritis, ST | 158/198 | 0.03 (-0.18, 0.25) | 0.04 (-0.28, 0.36) | 0.24 (-0.15, 0.63) | 0.763 | 0.826 | -0.95, 1.02 |
| Kroon FP, 2014 | SMP | UC/WL/No treatment | Osteoarthritis, IT | 981/1290 | -0.17 (-0.26, -0.08) | -0.17 (-0.26, -0.08) | -0.29 (-0.51, -0.07) | 1.6E-04 | 1.6E-04 | -0.27, -0.07 |
| Kroon FP, 2014 | SMP | No SMP | Osteoarthritis, IT | 52/66 | -0.19 (-0.56, 0.19) | -0.19 (-0.56, 0.19) | -0.13 (-0.62, 0.36) | 0.329 | 0.329 | NA |
| Kroon FP, 2014 | SMP | Control | Osteoarthritis, IT | 290/284 | -0.26 (-0.43, -0.09) | -0.26 (-0.43, -0.09) | -0.22 (-0.45, 0.01) | 0.003 | 0.003 | -1.38, 0.86 |
| Kroon FP, 2014 | SMP | Information | Osteoarthritis | 378/373 | -0.07 (-0.21, 0.08) | -0.07 (-0.21, 0.08) | -0.10 (-0.27, 0.06) | 0.371 | 0.371 | -1.00, 0.87 |
| Lakhan S, 2013 | MBT/MBCT | EDU/WL/Support | Fibromyalgia | 111/165 | -0.12 (-0.37, 0.13) | -0.12 (-0.37, 0.13) | -0.11 (-0.48, 0.27) | 0.352 | 0.352 | -0.66, 0.43 |
| Lakhan S, 2013 | MBT/MBCT | EDU/WL/Support | Irritable bowel syndrome | 82/78 | -0.59 (-0.91, -0.27) | -0.59 (-0.91, -0.27) | -0.64 (-1.08, -0.2) | 2.6E-04 | 2.6E-04 | NA |
| Lauche R, 2013 | MBSR | UC | Fibromyalgia Syndrome, LT | 99/91 | -0.01 (-0.30, 0.27) | -0.01 (-0.30, 0.27) | -0.05 (-0.42, 0.32) | 0.927 | 0.927 | NA |
| Lauche R, 2013 | MBSR | AC | Fibromyalgia Syndrome, LT | 89/85 | -0.10 (-0.39, 0.20) | -0.10 (-0.39, 0.20) | -0.13 (-0.51, 0.25) | 0.530 | 0.530 | NA |
| Lauche R, 2013 | MBSR | UC | Fibromyalgia Syndrome, ST | 146/177 | -0.23 (-0.46, -0.01) | -0.23 (-0.48, 0.01) | -0.07 (-0.44, 0.30) | 0.040 | 0.059 | -2.13, 1.67 |
| Lauche R, 2013 | MBSR | AC | Fibromyalgia Syndrome, ST | 102/124 | -0.44 (-0.73, -0.16) | -0.6 (-1.38, 0.17) | -0.32 (-0.7, 0.06) | 0.002 | 0.126 | -9.98, 8.77 |
| Macea DD, 2010 | Web-based CBT interventions | Control | Chronic pain | 1476/1482 | 0.23 (0.15, 0.31) | 0.29 (0.15, 0.43) | 0.28 (0.13, 0.42) | 2.7E-08 | 6.3E-05 | -0.07, 0.64 |
| Mustafa M, 2013 | Supportive/expressive group therapy | Usual treatment | Metastatic breast cancer | 104/175 | -0.58 (-0.99, -0.17) ^h^ | -0.58 (-0.99, -0.17) ^h^ | -0.75 (-1.36, -0.14) ^h^ | 0.005 | 0.005 | -3.21, 2.05 |
| Osborn RL, 2006 | EDU | Control | Cancer survivors | 122/128 | 0.23 (-0.02, 0.49) | 0.27 (-0.07, 0.61) | 0.06 (-0.27, 0.4) | 0.073 | 0.125 | -2.99, 3.53 |
| Peerdeman K, 2016 | Verbal suggestion/ Imagery | Control/ No treatment | Affective pain | 161/152 | 0.44 (0.25, 0.63) | 0.45 (0.20, 0.70) | 0.48 (0.10, 0.87) | 8.1E-06 | 3.5E-04 | -0.13, 1.04 |
| Peerdeman K, 2016 | Verbal suggestion | Control/ No treatment | Expected pain | 72/72 | 0.66 (0.43, 0.90) | 0.66 (0.43, 0.90) | 0.65 (0.24, 1.06) | 3.3E-08 | 3.3E-08 | 0.28, 1.04 |
| Peerdeman K, 2016 | Conditioning | Control/ No treatment | Pain relief | 71/71 | 0.66 (0.39, 0.92) | 0.65 (0.18, 1.11) | 0.62 (0.30, 0.94) | 1.2E-06 | 0.006 | -4.25, 5.55 |
| Peerdeman K, 2016 | Imagery | Control/ No treatment | Pain relief | 192/191 | 0.30 (0.11, 0.49) | 0.28 (0.02, 0.53) | 0.20 (-0.17, 0.56) | 0.002 | 0.036 | -0.40, 0.95 |
| Peerdeman K, 2016 | Verbal suggestion | Control/ No treatment | Pain relief | 527/534 | 0.51 (0.41, 0.62) | 0.75 (0.50, 1.00) | 0.24 (0.06, 0.41) | 1.0E-21 | 4.1E-09 | -0.23, 1.73 |
| Roldan-Barraza C, 2014 | Psychosocial Intervention/ Psychosocial Intervention + Usual Treatment | Usual Treatment | MTMD (self-reported), LT | 117/121 | 0.13 (-0.13, 0.38) | 0.23 (-0.25, 0.70) | -0.15 (-0.49, 0.19) | 0.334 | 0.352 | -5.07, 5.52 |
| Roldan-Barraza C, 2014 | Psychosocial Intervention/ Psychosocial Intervention + Usual Treatment | Tailored Usual Treatment | MTMD (self-reported), LT | 210/193 | 0.66 (0.23, 1.09) | 0.66 (0.23, 1.09) | 0.80 (0.14, 1.46) | 0.003 | 0.003 | -2.13, 3.45 |
| Roldan-Barraza C, 2014 | Psychosocial Intervention/ Psychosocial Intervention + Usual Treatment | Usual Treatment | MTMD (self-reported), ST | 210/208 | 0.05 (-0.15, 0.24) | 0.08 (-0.18, 0.34) | -0.10 (-0.44, 0.24) | 0.647 | 0.560 | -0.55, 0.71 |
| Roldan-Barraza C, 2014 | Psychosocial Intervention/ Psychosocial Intervention + Usual Treatment | Tailored Usual Treatment | MTMD (self-reported), ST | 241/229 | 0.03 (-0.15, 0.21) | 0.07 (-0.22, 0.37) | 0.00 (-0.32, 0.32) | 0.766 | 0.633 | -1.12, 1.27 |
| Roldan-Barraza C, 2014 | Psychosocial Intervention/ Psychosocial Intervention + Usual Treatment | Usual Treatment | Muscle Pain, ST | 142/138 | 0.23 (-0.02, 0.48) | 0.23 (-0.02, 0.48) | 0.27 (-0.07, 0.61) | 0.067 | 0.067 | -0.31, 0.77 |
| Sheinfeld Gorin S, 2012 | Psychological intervention | Control | Cancer pain severity | 1957/2313 | 0.34 (0.27, 0.40) | 0.34 (0.23, 0.46) | 0.14 (-0.08, 0.36) | 1.8E-22 | 7.2E-09 | -0.21, 0.89 |
| Sielski R, 2016 | Biofeedback/ EMG-Biofeedback | Control | Chronic back, follow-up | 212/259 | 0.55 (0.43, 0.67) | 0.62 (0.40, 0.84) | 0.31 (0.08, 0.54) | 1.2E-19 | 2.3E-08 | -0.09, 1.34 |
| Sielski R, 2016 | Biofeedback/ EMG-Biofeedback | Control | Chronic back, post-treatment | 486/573 | 0.57 (0.50, 0.65) | 0.61 (0.44, 0.77) | 0.33 (0.13, 0.52) | <1E-30 | 7.9E-13 | -0.13, 1.34 |
| Sprenger L, 2011 | Psychoeducation, IM, REL, Biofeedback, CBT | No treatment/ Paediatric standard care | Recurrent abdominal in children | 223/226 | 1.22 (1.21, 1.23) | 0.73 (0.33, 1.13) | 1.22 (1.21, 1.23) | <1E-30 | 3.2E-04 | -0.63, 2.10 |
| Theadom A, 2015 | Psychological therapies | UC | Fibromyalgia, 3m | 56/59 | -0.85 (-1.76, 0.06) | -0.85 (-1.76, 0.06) | -0.9 (-1.91, 0.11) | 0.067 | 0.067 | NA |
| Theadom A, 2015 | Psychological therapies | AtC | Fibromyalgia, 3m | 52/63 | 0.13 (-0.24, 0.50) | 0.13 (-0.24, 0.50) | 0.19 (-0.29, 0.67) | 0.494 | 0.494 | NA |
| Theadom A, 2015 | Psychological therapies | UC | Fibromyalgia, 6m | 180/191 | -0.51 (-0.72, -0.30) | -0.52 (-0.76, -0.29) | -0.38 (-0.75, -0.02) | 1.7E-06 | 1.5E-05 | -1.06, 0.02 |
| Theadom A, 2015 | Mindfulness | UC | Fibromyalgia, post-intervention | 68/60 | -0.09 (-0.43, 0.26) | -0.09 (-0.43, 0.26) | -0.07 (-0.47, 0.33) | 0.624 | 0.624 | NA |
| Theadom A, 2015 | Psychological therapies | UC | Fibromyalgia, post-intervention | 215/238 | -0.33 (-0.52, -0.15) | -0.33 (-0.52, -0.15) | -0.23 (-0.6, 0.15) | 4.8E-04 | 4.8E-04 | -0.56, -0.11 |
| Theadom A, 2015 | Psychological therapies | AtC | Fibromyalgia, post-intervention | 140/184 | -0.28 (-0.51, -0.06) | -0.30 (-0.66, 0.05) | -0.66 (-1.1, -0.21) | 0.013 | 0.097 | -1.46, 0.85 |
| Theadom A, 2015 | REL | UC | Fibromyalgia, post-intervention | 34/33 | -1.03 (-1.55, -0.50) | -0.82 (-2.29, 0.64) | -1.54 (-2.19, -0.89) | 1.4E-04 | 0.272 | NA |
| Uman LS, 2013 | Distraction | Control | Needle-related (children, adolescents), Behavioural measures | 75/77 | -0.20 (-0.52, 0.11) | -0.15 (-0.68, 0.39) | -0.39 (-0.78, 0.00) | 0.208 | 0.584 | NA |
| Uman LS, 2013 | Distraction | Control | Needle-related (children, adolescents), observer-reported | 200/247 | -0.82 (-1.02, -0.61) | -0.87 (-1.75, 0.02) | -1.34 (-1.74, -0.95) | 9.1E-15 | 0.054 | -4.29, 2.56 |
| Uman LS, 2013 | CBT-combined | Control | Needle-related (children, adolescents), self-reported | 126/124 | -0.34 (-0.59, -0.09) | -0.59 (-1.62, 0.44) | -0.34 (-0.62, -0.07) | 0.008 | 0.261 | -13.01, 11.83 |
| Uman LS, 2013 | Hypnosis | Control | Needle-related (children, adolescents), self-reported | 79/97 | -1.23 (-1.58, -0.88) | -1.4 (-2.32, -0.47) | 0.09 (-0.57, 0.74) | 3.2E-12 | 0.003 | -4.81, 2.01 |
| Uman LS, 2013 | Parent coaching + child distraction | Standard Care | Needle-related (children, adolescents), self-reported | 328/284 | 0.07 (-0.09, 0.23) | 0.05 (-0.20, 0.30) | 0.09 (-0.08, 0.27) | 0.384 | 0.675 | -2.21, 2.31 |
| Uman LS, 2013 | Preparation and information | Control | Needle-related (children, adolescents), self-reported | 78/76 | -0.34 (-0.66, -0.02) | -0.22 (-1.20, 0.76) | -0.7 (-1.11, -0.30) | 0.040 | 0.665 | NA |
| Uman LS, 2013 | Suggestion | Control | Needle-related (children, adolescents), self-reported | 129/89 | -0.13 (-0.41, 0.15) | -0.13 (-0.41, 0.15) | -0.17 (-0.55, 0.21) | 0.354 | 0.354 | -1.91, 1.65 |
| Uman LS, 2013 | Virtual reality | Standard Care | Needle-related (children, adolescents), self-reported | 23/27 | -0.23 (-0.78, 0.33) | -0.23 (-0.78, 0.33) | -0.29 (-1.02, 0.43) | 0.430 | 0.430 | NA |
| Vellemain S, 2010 | Computerized CBT | WL/ EDU | Pain in children and adolescents | 73/77 | -0.41 (-0.74, -0.07) | -0.41 (-0.74, -0.07) | -0.55 (-1.13, 0.03) | 0.019 | 0.019 | -1.15, 0.34 |
| Williams AC, 2012 | Behavioural | TAU | Chronic (excl. HA), post-treatment | 230/254 | -0.10 (-0.29, 0.08) | -0.28 (-0.79, 0.24) | 0.09 (-0.22, 0.40) | 0.265 | 0.295 | -2.19, 1.64 |
| Williams AC, 2012 | Cognitive behavioural | AC | Chronic (excl. HA), post-treatment | 639/696 | -0.09 (-0.20, 0.02) | -0.09 (-0.22, 0.03) | -0.05 (-0.31, 0.21) | 0.101 | 0.146 | -0.39, 0.20 |
| Williams AC, 2012 | Cognitive behavioural | TAU | Chronic (excl. HA), post-treatment | 562/586 | -0.24 (-0.36, -0.13) | -0.21 (-0.37, -0.05) | -0.53 (-0.87, -0.19) | 4.7E-05 | 0.010 | -0.72, 0.29 |
| Williams AC, 2012 | Behavioural | TAU | Chronic pain (excl. HA), follow-up | 88/94 | -0.03 (-0.32, 0.26) | -0.03 (-0.32, 0.26) | -0.05 (-0.44, 0.34) | 0.828 | 0.828 | NA |
| Williams AC, 2012 | Cognitive behavioural | AC | Chronic pain (excl. HA), follow-up | 667/667 | -0.07 (-0.17, 0.04) | -0.08 (-0.21, 0.06) | -0.12 (-0.37, 0.14) | 0.237 | 0.281 | -0.42, 0.26 |
| Williams AC, 2012 | Cognitive behavioural | TAU | Chronic pain (excl. HA), follow-up | 325/310 | -0.09 (-0.25, 0.06) | -0.09 (-0.25, 0.08) | -0.14 (-0.42, 0.15) | 0.243 | 0.311 | -0.38, 0.20 |

Abbreviations: AC: Active control; AtC: Attention control; ASMP: Arthritis Self-Management Program; Biofeedback: biofeedback; CBT: cognitive behavioural therapy; EDU: education; EMG Biofeedback: Electromyographic biofeedback; HA: headache; HYP: hypnotherapy; IM: imagination; IT: Intermediate term; LT: Long term; m: months; MBT: Mindfulness-based therapy; MBCT: Mindfulness-based cognitive therapy; MBPSR: multidisciplinary bio-psychosocial rehabilitation programs; MBR: Multidisciplinary biopsychological rehabilitation; MBSR: Mindfulness-based stress reduction; MTMD, Myofascial Temporomandibular Disorder; NA: Not applicable, because only two studies were available; NR: not reported; REL: relaxation; RIMH: relaxation, guided imagery, meditation or hypnosis; SGT: Supportive group therapy; SMP: Self-management education programmes; ST: Short term; St. Care: Standard care; TAU: Treated as usual; UC: Usual Care; WL, Waiting list.

^a^ All summary point estimates on this table were indicative of pain reduction comparing the intervention to the control group. However, the original meta-analyses reported both positive and negative effects as observed on this table because they used different outcome metrics (e.g., pain reduction or difference in pain levels).

^b^ Fixed effects refers to summary effect (95% CI) using the meta-analysis fixed-effects model.

^c^ Random effects refers to summary effect (95% CI) using the meta-analysis random-effects model.

^d^ Relative risk and 95% confidence interval of largest study (smallest SE) in each meta-analysis.
^e^ P value of summary fixed effects estimate.
^f^ P value of summary random effects estimate.

^g^ Prediction intervals are reported only for meta-analyses including at least 3 studies.

^h^ On these comparisons MD is reported, instead of SMD.

**Table S2:** Evaluation of bias and heterogeneity in the 150 meta-analyses investigating the effectiveness of various psychological interventions for pain reduction.

| **Author, Year** | **Intervention Group** | **Control group** | **Type of pain** | **Control/ Intervention N** | **Egger’s  P-value ^a, g^** | **Effect for SE=0 ^b, g^** | **I2 (95% CI;  P-value) ^c, g^** | **Studies** | **Excess significance ^d^** | |
| --- | --- | --- | --- | --- | --- | --- | --- | --- | --- | --- |
|  |  |  |  |  |  |  |  |  | **O/E ^e^** | **P-value ^f^** |
| Adachi T, 2013 | Hypnosis | St. Care | Chronic, post-intervention | 79/84 | 0.22 | -0.43 | 57 (0-84; 0.07) | 4 | 1/0.83 | 1 |
| Adachi T, 2013 | Hypnosis | Other psychological treatment | Chronic, post-intervention | 246/259 | 0.15 | -0.95 | 51 (0-73; 0.02) | 12 | 2/9.26 | NP |
| Adachi T, 2013 | Hypnosis | Other psychological treatment | Chronic, follow-up | 130/151 | 0.88 | -0.01 | 15 (0-64; 0.32) | 7 | 1/2.62 | NP |
| Aqqarwal VR, 2011 | Any psychosocial intervention | Usual treatment | Muscle palpation, >3m | 69/74 | 0.61 | -0.95 | 0 (0-73; 0.76) | 3 | 1/2.05 | NP |
| Aqqarwal VR, 2011 | CBT | Usual treatment | Orofacial, >3m | 187/196 | 0.91 | -0.3 | 0 (0-68; 0.59) | 4 | 1/3.11 | NP |
| Aqqarwal VR, 2011 | CBT+Biofeedback | Usual treatment | Orofacial, >3m | 81/115 | 0.47 | -1.44 | 53 (0-85; 0.12) | 3 | 1/3 | NP |
| Aqqarwal VR, 2011 | Biofeedback | Usual treatment | Orofacial, ≤3m | 15/30 | NA | NA | 35 (NA; 0.22) | 2 | 0/0.16 | NP |
| Aqqarwal VR, 2011 | CBT | Usual treatment | Orofacial, ≤3m | 203/208 | 0.67 | 0.22 | 0 (0-68; 0.40) | 4 | 0/2.53 | NP |
| Aqqarwal VR, 2011 | CBT+Biofeedback | Usual treatment | Orofacial, ≤3m | 36/54 | NA | NA | 45 (NA; 0.18) | 2 | 1/1.95 | NP |
| Aqqarwal VR, 2011 | HYP | REL | Orofacial, ≤3m | 39/42 | NA | NA | 0 (NA; 0.76) | 2 | 1/1.26 | NP |
| Bawa F, 2015 | Mindfulness | AC | Chronic pain intensity | 203/146 | 0.09 | 0.53 | 0 (0-64; 0.67) | 5 | 0/0.64 | NP |
| Bawa F, 2015 | Mindfulness | Inactive Control | Chronic pain intensity | 49/55 | 0.52 | -0.37 | 0 (0-68; 0.57) | 4 | 1/0.8 | 0.59 |
| Bernardy K, 2013 | CBT | AC/AtC/EDU/TAU/Support | Fibromyalgia (self-efficacy), end of treatment | 275/314 | 0.88 | -0.23 | 74 (41-85; <0.001) | 9 | 5/8.99 | NP |
| Bernardy K, 2013 | Operant therapy | AC/EDU/TAU | Fibromyalgia (self-efficacy), end of treatment | 40/83 | NA | NA | 95 (NA; <0.001) | 2 | 1/0.88 | 1.00 |
| Bernardy K, 2013 | CBT | AC/AtC/EDU/TAU/Support | Fibromyalgia (self-efficacy), LT | 229/265 | 0.82 | -0.20 | 86 (74-91; <0.001) | 8 | 3/8 | NP |
| Bernardy K, 2013 | Operant therapy | AC/EDU/TAU | Fibromyalgia (self-efficacy), LT | 40/83 | NA | NA | 83 (NA; 0.02) | 2 | 2/2 | 1.00 |
| Bernardy K, 2013 | CBT | AC/AtC/EDU/TAU/Support | Fibromyalgia, end of treatment | 547/603 | 0.37 | -0.52* | 30 (0-59; 0.11) | 18 | 4/17.17 | NP |
| Bernardy K, 2013 | Operant therapy | AC/AtC/EDU/Support | Fibromyalgia, end of treatment | 40/83 | NA | NA | 95 (NA; <0.001) | 2 | 1/1.15 | NP |
| Bernardy K, 2013 | Self-management | AC/TAU | Fibromyalgia, end of treatment | 94/86 | NA | NA | 85 (NA; 0.01) | 2 | 1/1.81 | NP |
| Bernardy K, 2013 | CBT | AC/AtC/EDU/TAU/Support | Fibromyalgia, LT | 360/410 | 0.64 | -0.43 | 2 (0-50; 0.43) | 13 | 3/9.36 | NP |
| Bernardy K, 2013 | Operant therapy | AC/EDU/TAU | Fibromyalgia, LT | 40/83 | NA | NA | 83 (NA; 0.01) | 2 | 2/2 | 1.00 |
| Bernardy K, 2011 | HYP | CBT/ TAU/ WL/ Attention placebo | Fibromyalgia | 80/98 | 0.84 | -0.66 | 88 (75-93; <0.001) | 6 | 3/5.99 | NP |
| Birnie K, 2014 | Distraction | NR | Needle-related (children, adolescents), self-reported | 1229/1243 | 0.13 | 0.05 | 86 (80-89; <0.001) | 24 | 7/3.4 | 0.07 |
| Champaneria, 2012 | Psychological intervention | No psychological intervention | Chronic pelvic, ≥6m | 76/63 | NA | NA | 95 (NA; <0.001) | 2 | 1/0.67 | 1.00 |
| Champaneria, 2012 | Psychological intervention | No psychological intervention | Chronic pelvic, 3m | 79/77 | NA | NA | 96 (NA; <0.001) | 2 | 1/1.88 | NP |
| Damen L, 2006 | Biofeedback | Control | HA Post-treatment | 34/37 | 0.04 | 7.43* | 0 (0-73; 0.78) | 3 | 0/2.74 | NP |
| Damen L, 2006 | REL + Biofeedback + CBT | WL | HA Post-treatment | 15/38 | NA | NA | 0 (NA; 0.69) | 2 | 0/1.4 | NP |
| Damen L, 2006 | REL + CBT | Attention placebo | HA Post-treatment | 34/35 | NA | NA | 0 (NA; 0.66) | 2 | 0/1.21 | NP |
| Damen L, 2006 | REL+Biofeedback | WL | HA Post-treatment | 24/20 | NA | NA | 76 (NA; 0.04) | 2 | 2/1.14 | 0.51 |
| Dixon K, 2007 | CBT/ Stress management/ HYP | NR | Arthritis | 1136/1167 | 0.02 | -0.05 | 9 (0-47; 0.34) | 20 | 5/5.03 | NP |
| Du S, 2011 | ASMP/ Self-management | WL/ UC/ Conventional/No treatment | Chronic musculoskeletal, 12m | 770/800 | 0.17 | 0.03 | 0 (0-64; 0.45) | 5 | 1/0.71 | 0.53 |
| Du S, 2011 | ASMP/ Self-management | WL/ UC/ Conventional/No treatment | Chronic musculoskeletal, 4m | 1279/1689 | 0.73 | -0.15 | 59 (0-79; 0.02) | 8 | 4/7.94 | NP |
| Du S, 2011 | ASMP/ Self-management | WL/ UC/ Conventional/No treatment | Chronic musculoskeletal, 6m | 524/494 | 0.25 | -0.21 | 0 (0-73; 0.84) | 3 | 3/2.78 | 1.00 |
| Eccleston C, 2014 | Psychological therapies (Internet-delivered) | AC/TAU/WL | Chronic (Non-HA), follow-up | 617/585 | 0.59 | 0.21 | 96 (94-98; <0.001) | 4 | 1/2.17 | NP |
| Eccleston C, 2014 | Psychological therapies (Internet-delivered) | AC/TAU/WL | Chronic (Non-HA), post-treatment | 903/882 | 0.42 | -0.12 | 77 (56-86; <0.001) | 11 | 5/5.96 | NP |
| Eccleston C, 2014 | Psychological therapies | Control | Chronic and recurrent HA (children, adolescents), follow-up | 111/140 | <0.001 | 0.02 | 60 (0-83; 0.04) | 5 | 2/1.4 | 0.62 |
| Eccleston C, 2014 | Psychological therapies | Control | Chronic and recurrent HA (children, adolescents), post-treatment | 287/427 | <0.001 | 0.08 | 25 (0-59; 0.18) | 15 | 7/7.46 | NP |
| Eccleston C, 2014 | Psychological therapies | Control | Chronic and recurrent non-HA (children, adolescents), follow-up | 270/273 | 0.23 | 0.47 | 62 (0-81; 0.01) | 7 | 1/1.95 | NP |
| Eccleston C, 2014 | Psychological therapies | Control | Chronic and recurrent non-HA (children, adolescents), post-treatment | 404/448 | <0.001 | 0.77* | 75 (54-84; <0.001) | 13 | 5/4.58 | 0.78 |
| Eccleston C, 2014 | Psychological therapies (Internet-delivered) | AC/TAU/WL | Chronic HA, post-treatment | 72/59 | NA | NA | 0 (NA; 0.53) | 2 | 2/1.94 | 1.00 |
| Fisher E, 2014 | CBT/ Biofeedback/ REL/ HYP | WL/ EDU/ St. Care/ Self-monitoring | Chronic (excluding HA) | 310/362 | <0.001 | 0.73* | 71 (38-83; <0.001) | 11 | 4/3.65 | 0.76 |
| Fisher E, 2014 | CBT/ Biofeedback/ REL/ HYP | WL/ EDU/ St. Care/ Self-monitoring | Headache | 289/459 | <0.001 | -0.05 | 16 (0-52; 0.26) | 18 | 7/6.65 | 1.00 |
| Flanagan E, 2015 | CBT | Medical treatment | General vaginal, post-treatment | 56/87 | 0.84 | -0.09 | 28 (0-80; 0.25) | 3 | 1/0.51 | 0.43 |
| Flanagan E, 2015 | CBT | Medical treatment | Pain on intercourse, 6m | 39/62 | NA | NA | 39 (NA; 0.20) | 2 | 0/0.67 | NP |
| Flanagan E, 2015 | CBT | Other psychological treatment | Pain on intercourse, follow-up | 42/41 | NA | NA | 69 (NA; 0.07) | 2 | 1/2 | NP |
| Flanagan E, 2015 | CBT | Medical treatment | Pain on intercourse, post-treatment | 44/66 | NA | NA | 72 (NA; 0.06) | 2 | 1/0.16 | 0.15 |
| Flanagan E, 2015 | CBT | Other psychological treatment | Pain on intercourse, post-treatment | 67/81 | 0.85 | -0.75 | 13 (0-76; 0.32) | 3 | 0/0.16 | NP |
| Flanagan E, 2015 | CBT + behavioural | WL | Pain on intercourse, post-treatment | 58/66 | NA | NA | 95 (NA; <0.001) | 2 | 1/0.22 | 0.21 |
| Glombiewski JA, 2010 | EDU/ CBT/ REL | CBT/ TAU/ WL/ Attention placebo | Fibromyalgia | 489/528 | <0.001 | -0.16 | 69 (49-79; <0.001) | 21 | 14/3.15 | <0.001 |
| Guzman J, 2002 | Less intensive (<30 h) once or twice weekly MBPSR | NR | Low back, 12m | 177/188 | 0.84 | 0.27 | 53 (0-85; 0.12) | 3 | 0/0.94 | NP |
| Guzman J, 2002 | Other types of MBPSR | NR | Low back, 12m | 205/237 | NA | NA | 0 (NA; 0.45) | 2 | 0/0.23 | NP |
| Guzman J, 2002 | Less intensive (<30 h) once or twice weekly MBPSR | NR | Low back, 24-30m | 155/165 | NA | NA | 0 (NA; 0.40) | 2 | 0/0.63 | NP |
| Guzman J, 2002 | Intensive (>100h) daily MBPSR with functional restoration | NR | Low back, 24m | 77/90 | NA | NA | 65 (NA; 0.09) | 2 | 1/0.72 | 1.00 |
| Guzman J, 2002 | Intensive (>100h) daily MBPSR with functional restoration | NR | Low back, 3-4m | 80/85 | NA | NA | 0 (NA; 0.37) | 2 | 2/1.94 | 1.00 |
| Guzman J, 2002 | Less intensive (<30 h) once or twice weekly MBPSR | NR | Low back, 3-6m | 206/214 | 0.80 | -0.22 | 63 (0-85; 0.04) | 4 | 0/1.59 | NP |
| Guzman J, 2002 | Intensive (>100h) daily MBPSR with functional restoration | NR | Low back, 60m | 71/83 | NA | NA | 58 (NA; 0.12) | 2 | 1/0.1 | 0.10 |
| Guzman J, 2002 | Less intensive (<30 h) once or twice weekly MBPSR | NR | Low back, at treatment completion | 65/77 | 0.92 | -0.42 | 69 (0-89; 0.04) | 3 | 1/0.21 | 0.19 |
| Henrich J, 2015 | Psychological therapies | Control | Irritable bowel syndrome | 1038/1207 | 0.01 | 0.03 | 26 (0-52; 0.09) | 32 | 9/2.22 | <0.001 |
| Henschke N, 2011 | Behavioural treatment | UC | Chronic low back, IT | 155/164 | NA | NA | 0 (NA; 0.57) | 2 | 0/1.35 | NP |
| Henschke N, 2011 | Behavioural treatment | Group exercise | Chronic low back, IT | 68/69 | NA | NA | 0 (NA; 0.53) | 2 | 0/0.12 | NP |
| Henschke N, 2011 | Behavioural treatment + physiotherapy | Physiotherapy | Chronic low back, IT | 16/29 | NA | NA | 0 (NA; 0.46) | 2 | 0/0.62 | NP |
| Henschke N, 2011 | CBT | Cognitive therapy | Chronic low back, IT | 19/25 | NA | NA | 90 (NA; <0.001) | 2 | 2/1.72 | 1.00 |
| Henschke N, 2011 | CBT | Operant therapy | Chronic low back, IT | 65/75 | 0.76 | -0.05 | 0 (0-73; 0.61) | 3 | 0/0.3 | NP |
| Henschke N, 2011 | CBT | Respondent therapy | Chronic low back, IT | 32/30 | NA | NA | 66 (NA; 0.09) | 2 | 1/0.11 | 0.10 |
| Henschke N, 2011 | Cognitive therapy | Operant therapy | Chronic low back, IT | 39/43 | NA | NA | 49 (NA; 0.16) | 2 | 0/0.11 | NP |
| Henschke N, 2011 | Behavioural treatment | Group exercise | Chronic low back, LT | 67/69 | NA | NA | 0 (NA; 0.66) | 2 | 0/0.13 | NP |
| Henschke N, 2011 | CBT | Cognitive therapy | Chronic low back, LT | 18/30 | NA | NA | 91 (NA; <0.001) | 2 | 1/0.91 | 1.00 |
| Henschke N, 2011 | CBT | Operant therapy | Chronic low back, LT | 67/73 | 0.77 | -0.21 | 0 (0-73; 0.88) | 3 | 0/1.05 | NP |
| Henschke N, 2011 | Behavioural treatment | UC | Chronic low back, ST | 163/167 | NA | NA | 20 (NA; 0.26) | 2 | 0/1.16 | NP |
| Henschke N, 2011 | Behavioural treatment | Group exercise | Chronic low back, ST | 73/73 | NA | NA | 0 (NA; 0.40) | 2 | 0/1.27 | NP |
| Henschke N, 2011 | Behavioural treatment + physiotherapy | Physiotherapy | Chronic low back, ST | 18/41 | NA | NA | 73 (NA; 0.05) | 2 | 0/1.8 | NP |
| Henschke N, 2011 | Behavioural treatment + physiotherapy | Inpatient rehabilitation | Chronic low back, ST | 214/191 | NA | NA | 0 (NA; 0.48) | 2 | 0/1.09 | NP |
| Henschke N, 2011 | CBT | WL | Chronic low back, ST | 110/129 | 0.34 | 0.02 | 43 (0-78; 0.14) | 5 | 2/4.12 | NP |
| Henschke N, 2011 | CBT | Cognitive therapy | Chronic low back, ST | 24/37 | NA | NA | 76 (NA; 0.04) | 2 | 0/0.65 | NP |
| Henschke N, 2011 | CBT | Operant therapy | Chronic low back, ST | 76/85 | 0.32 | -0.51 | 0 (0-73; 0.80) | 3 | 0/0.76 | NP |
| Henschke N, 2011 | CBT | Respondent therapy | Chronic low back, ST | 47/50 | 0.27 | 2.97 | 0 (0-73; 0.56) | 3 | 0/0.84 | NP |
| Henschke N, 2011 | Cognitive therapy | WL | Chronic low back, ST | 39/29 | NA | NA | 12 (NA; 0.29) | 2 | 0/1.69 | NP |
| Henschke N, 2011 | Cognitive therapy | Operant therapy | Chronic low back, ST | 44/49 | NA | NA | 71 (NA; 0.07) | 2 | 0/0.1 | NP |
| Henschke N, 2011 | Operant therapy | WL | Chronic low back, ST | 70/83 | 0.32 | -1.81 | 0 (0-73; 0.59) | 3 | 1/2.95 | NP |
| Henschke N, 2011 | Respondent therapy (EMG Biofeedback) | WL | Chronic low back, ST | 30/34 | 0.76 | -2.23 | 0 (0-73; 0.40) | 3 | 1/2.99 | NP |
| Henschke N, 2011 | Respondent therapy (progressive REL) | WL | Chronic low back, ST | 35/39 | 0.53 | 33.89 | 57 (0-86; 0.10) | 3 | 1/1.94 | NP |
| Johannsen M, 2013 | EDU/ RIMH/ SGT | WL/ St. Care/ NR | Breast cancer patients/survivors | 755/1015 | 0.03 | 0.02 | 38 (0-62; 0.04) | 21 | 7/2.71 | 0.01 |
| Kamper SJ, 2014 | MBR | UC | Chronic low back, IT | 369/371 | 0.01 | 0.4 | 63 (0-83; 0.02) | 6 | 5/3.95 | 0.67 |
| Kamper SJ, 2014 | MBR | Physical treatment | Chronic low back, IT | 265/266 | 0.17 | 0.31 | 51 (0-75; 0.04) | 9 | 2/0.54 | 0.10 |
| Kamper SJ, 2014 | MBR | UC | Chronic low back, LT | 373/448 | 0.68 | -0.06 | 26 (0-68; 0.23) | 7 | 2/5.94 | NP |
| Kamper SJ, 2014 | MBR | Physical treatment | Chronic low back, LT | 437/435 | 0.26 | 0.54 | 92 (88-95; <0.001) | 9 | 2/3.08 | NP |
| Kamper SJ, 2014 | MBR | Surgery | Chronic low back, LT | 188/197 | NA | NA | 48 (NA; 0.16) | 2 | 1/0.75 | 1.00 |
| Kamper SJ, 2014 | MBR | UC | Chronic low back, ST | 446/433 | 0.29 | -0.08 | 72 (35-84; <0.001) | 9 | 5/3.94 | 0.52 |
| Kamper SJ, 2014 | MBR | Physical treatment | Chronic low back, ST | 851/810 | 0.62 | -0.12 | 80 (65-87; <0.001) | 12 | 3/4.53 | NP |
| Kamper SJ, 2014 | MBR | WL | Chronic low back, ST | 107/106 | 0.78 | -0.17 | 63 (0-88; 0.06) | 3 | 2/2.66 | NP |
| Kisely SR, 2015 | Psychological intervention | No psychological intervention | Chest (frequency), ≤3m | 140/154 | 0.29 | -0.23 | 94 (90-96; <0.001) | 7 | 4/0.45 | <0.001 |
| Kisely SR, 2015 | Psychological intervention | No psychological intervention | Chest (frequency), 3-12m | 80/84 | 0.30 | 0.7 | 75 (0-89; 0.01) | 4 | 1/0.6 | 0.48 |
| Kisely SR, 2015 | Psychological intervention | No psychological intervention | Chest (severity), ≤3m | 86/94 | 0.36 | -0.47 | 65 (0-86; 0.04) | 4 | 2/1.17 | 0.59 |
| Kisely SR, 2015 | Psychological intervention | No psychological intervention | Chest, ≤3m | 82/90 | 0.75 | 0.07 | 58 (0-86; 0.09) | 3 | 2/2.98 | NP |
| Kisely SR, 2015 | Psychological intervention | No psychological intervention | Chest, 3-12m | 61/50 | NA | NA | 0 (NA; 0.95) | 2 | 2/2 | 1.00 |
| Knittle K, 2010 | Self-regulation | WL/ St. Care/ No Intervention | Rheumatoid Arthritis | 638/678 | 0.53 | 0.1 | 0 (0-41; 0.94) | 22 | 1/3.73 | NP |
| Koranyi S, 2014 | Psychological intervention | Control | Acute pain after open heart surgery, IT | 210/203 | 0.55 | 0.46 | 35 (0-78; 0.20) | 4 | 0/1.68 | NP |
| Koranyi S, 2014 | Psychological intervention | TAU | Acute pain after open heart surgery, IT | 150/143 | 0.07 | 0.46 | 0 (0-73; 0.75) | 3 | 0/1.26 | NP |
| Koranyi S, 2014 | Psychological intervention | Control | Acute pain after open heart surgery, LT | 143/137 | 0.89 | -0.01 | 0 (0-73; 0.58) | 3 | 0/0.3 | NP |
| Kroon FP, 2014 | SMP | UC/WL/No treatment | Osteoarthritis, ST | 282/473 | 0.89 | -0.21 | 0 (0-61; 0.43) | 6 | 2/5.09 | NP |
| Kroon FP, 2014 | SMP | No SMP | Osteoarthritis, ST | 158/198 | 0.88 | -0.17 | 52 (0-81; 0.08) | 5 | 0/2.46 | NP |
| Kroon FP, 2014 | SMP | UC/WL/No treatment | Osteoarthritis, IT | 981/1290 | 0.12 | -0.35* | 0 (0-49; 0.77) | 13 | 2/10.85 | NP |
| Kroon FP, 2014 | SMP | No SMP | Osteoarthritis, IT | 52/66 | NA | NA | 0 (NA; 0.73) | 2 | 0/0.33 | NP |
| Kroon FP, 2014 | SMP | Control | Osteoarthritis, IT | 290/284 | 0.27 | -0.11 | 0 (0-73; 0.87) | 3 | 0/2.16 | NP |
| Kroon FP, 2014 | SMP | Information | Osteoarthritis | 378/373 | 0.14 | -0.19 | 0 (0-73; 0.69) | 3 | 0/0.97 | NP |
| Lakhan S, 2013 | MBT/MBCT | EDU/WL/Support | Fibromyalgia | 111/165 | 0.68 | 0.11 | 0 (0-68; 0.72) | 4 | 0/0.59 | NP |
| Lakhan S, 2013 | MBT/MBCT | EDU/WL/Support | Irritable bowel syndrome | 82/78 | NA | NA | 0 (NA; 0.76) | 2 | 2/2 | 1.00 |
| Lauche R, 2013 | MBSR | UC | Fibromyalgia Syndrome, LT | 99/91 | NA | NA | 0 (NA; 0.76) | 2 | 0/0.15 | NP |
| Lauche R, 2013 | MBSR | AC | Fibromyalgia Syndrome, LT | 89/85 | NA | NA | 0 (NA; 0.77) | 2 | 0/0.44 | NP |
| Lauche R, 2013 | MBSR | UC | Fibromyalgia Syndrome, ST | 146/177 | 0.97 | -0.41 | 15 (0-77; 0.31) | 3 | 1/0.33 | 0.30 |
| Lauche R, 2013 | MBSR | AC | Fibromyalgia Syndrome, ST | 102/124 | 0.53 | 0.93 | 84 (18-93; <0.001) | 3 | 1/2.06 | NP |
| Macea DD, 2010 | Web-based CBT interventions | Control | Chronic pain | 1476/1482 | 0.11 | 0.1 | 45 (0-71; 0.05) | 11 | 3/6.99 | NP |
| Mustafa M, 2013 | Supportive/expressive group therapy | Usual treatment | Metastatic breast cancer | 104/175 | 0.90 | -0.83 | 0 (0-73; 0.57) | 3 | 1/2.94 | NP |
| Osborn RL, 2006 | EDU | Control | Cancer survivors | 122/128 | 0.56 | -0.33 | 38 (0-82; 0.20) | 3 | 1/0.25 | 0.23 |
| Peerdeman K, 2016 | Verbal suggestion/ Imagery | Control/ No treatment | Affective pain | 161/152 | 0.35 | 0.04 | 34 (0-71; 0.17) | 7 | 4/5.78 | NP |
| Peerdeman K, 2016 | Verbal suggestion | Control/ No treatment | Expected pain | 72/72 | 0.66 | 0.55 | 0 (0-64; 0.94) | 5 | 3/4.68 | NP |
| Peerdeman K, 2016 | Conditioning | Control/ No treatment | Pain relief | 71/71 | 0.92 | 0.75 | 55 (0-86; 0.11) | 3 | 2/2.83 | NP |
| Peerdeman K, 2016 | Imagery | Control/ No treatment | Pain relief | 192/191 | 0.23 | 0.93 | 42 (0-76; 0.12) | 6 | 1/2.27 | NP |
| Peerdeman K, 2016 | Verbal suggestion | Control/ No treatment | Pain relief | 527/534 | <0.001 | -0.06 | 78 (63-85; <0.001) | 18 | 11/7.51 | 0.15 |
| Roldan-Barraza C, 2014 | Psychosocial Intervention/ Psychosocial Intervention + Usual Treatment | Usual Treatment | MTMD (self-reported), LT | 117/121 | 0.40 | -0.75 | 66 (0-88; 0.05) | 3 | 1/0.77 | 1.00 |
| Roldan-Barraza C, 2014 | Psychosocial Intervention/ Psychosocial Intervention + Usual Treatment | Tailored Usual Treatment | MTMD (self-reported), LT | 210/193 | 0.54 | 2.2 | 0 (0-73; 0.58) | 3 | 2/2.94 | NP |
| Roldan-Barraza C, 2014 | Psychosocial Intervention/ Psychosocial Intervention + Usual Treatment | Usual Treatment | MTMD (self-reported), ST | 210/208 | 0.24 | -0.39 | 37 (0-72; 0.15) | 7 | 0/0.83 | NP |
| Roldan-Barraza C, 2014 | Psychosocial Intervention/ Psychosocial Intervention + Usual Treatment | Tailored Usual Treatment | MTMD (self-reported), ST | 241/229 | 0.11 | -1.24 | 61 (0-85; 0.05) | 4 | 1/0.2 | 0.19 |
| Roldan-Barraza C, 2014 | Psychosocial Intervention/ Psychosocial Intervention + Usual Treatment | Usual Treatment | Muscle Pain, ST | 142/138 | 0.92 | 0.21 | 0 (0-68; 0.74) | 4 | 0/1.97 | NP |
| Sheinfeld Gorin S, 2012 | Psychological intervention | Control | Cancer pain severity | 1957/2313 | 0.67 | 0.28 | 60 (40-71.; <0.01) | 38 | 17/9.63 | 0.01 |
| Sielski R, 2016 | Biofeedback/ EMG-Biofeedback | Control | Chronic back, follow-up | 212/259 | 0.08 | -0.07 | 67 (27-81; <0.001) | 11 | 9/8.36 | 1.00 |
| Sielski R, 2016 | Biofeedback/ EMG-Biofeedback | Control | Chronic back, post-treatment | 486/573 | 0.43 | 0.36 | 78 (67-85; <0.001) | 22 | 15/19.03 | NP |
| Sprenger L, 2011 | Psychoeducation, IM, REL, Biofeedback, CBT | No treatment/ Paediatric standard care | Recurrent abdominal in children | 223/226 | 0.04 | 1.23 | 94 (92-96; <0.001) | 9 | 4/8.96 | NP |
| Theadom A, 2015 | Psychological therapies | UC | Fibromyalgia, 3m | 56/59 | NA | NA | 0 (NA; 0.82) | 2 | 0/1.32 | NP |
| Theadom A, 2015 | Psychological therapies | AtC | Fibromyalgia, 3m | 52/63 | NA | NA | 0 (NA; 0.70) | 2 | 0/0.58 | NP |
| Theadom A, 2015 | Psychological therapies | UC | Fibromyalgia, 6m | 180/191 | 0.15 | 0.34 | 20 (0-71; 0.29) | 5 | 2/4.12 | NP |
| Theadom A, 2015 | Mindfulness | UC | Fibromyalgia, post-intervention | 68/60 | NA | NA | 0 (NA; 0.87) | 2 | 0/0.17 | NP |
| Theadom A, 2015 | Psychological therapies | UC | Fibromyalgia, post-intervention | 215/238 | 0.90 | -0.28 | 0 (0-54; 0.6) | 9 | 2/3.1 | NP |
| Theadom A, 2015 | Psychological therapies | AtC | Fibromyalgia, post-intervention | 140/184 | 0.53 | 0.79 | 60 (0-83; 0.04) | 5 | 2/4.97 | NP |
| Theadom A, 2015 | REL | UC | Fibromyalgia, post-intervention | 34/33 | NA | NA | 86 (NA; 0.01) | 2 | 1/2 | NP |
| Uman LS, 2013 | Distraction | Control | Needle-related (children, adolescents), Behavioural measures | 75/77 | NA | NA | 61 (NA; 0.11) | 2 | 0/1.76 | NP |
| Uman LS, 2013 | Distraction | Control | Needle-related (children, adolescents), observer-reported | 200/247 | 0.73 | 0.59 | 94 (90-96; <0.001) | 5 | 2/5 | NP |
| Uman LS, 2013 | CBT-combined | Control | Needle-related (children, adolescents), self-reported | 126/124 | 0.78 | -0.07 | 86 (32-93; <0.001) | 3 | 2/1.62 | 1.00 |
| Uman LS, 2013 | Hypnosis | Control | Needle-related (children, adolescents), self-reported | 79/97 | 0.41 | 0.88 | 85 (61-92; <0.001) | 5 | 4/0.37 | <0.001 |
| Uman LS, 2013 | Parent coaching + child distraction | Standard Care | Needle-related (children, adolescents), self-reported | 328/284 | 0.82 | 0.13 | 24 (0-79; 0.27) | 3 | 0/0.71 | NP |
| Uman LS, 2013 | Preparation and information | Control | Needle-related (children, adolescents), self-reported | 78/76 | NA | NA | 88 (NA; <0.001) | 2 | 1/2 | NP |
| Uman LS, 2013 | Suggestion | Control | Needle-related (children, adolescents), self-reported | 129/89 | 0.49 | 0.3 | 0 (0-73; 0.38) | 3 | 0/0.88 | NP |
| Uman LS, 2013 | Virtual reality | Standard Care | Needle-related (children, adolescents), self-reported | 23/27 | NA | NA | 0 (NA; 0.78) | 2 | 0/0.57 | NP |
| Vellemain S, 2010 | Computerized CBT | WL/ EDU | Pain in children and adolescents | 73/77 | 0.53 | -0.86 | 0 (0-68; 0.78) | 4 | 0/3.29 | NP |
| Williams AC, 2012 | Behavioural | TAU | Chronic (excl. HA), post-treatment | 230/254 | 0.15 | 1.4 | 87 (67-92; <0.001) | 5 | 1/0.7 | 0.53 |
| Williams AC, 2012 | Cognitive behavioural | AC | Chronic (excl. HA), post-treatment | 639/696 | 0.57 | 0.08 | 25 (0-60; 0.18) | 14 | 3/1.08 | 0.09 |
| Williams AC, 2012 | Cognitive behavioural | TAU | Chronic (excl. HA), post-treatment | 562/586 | 0.02 | -1.27* | 45 (0-68; 0.03) | 16 | 4/15.61 | NP |
| Williams AC, 2012 | Behavioural | TAU | Chronic pain (excl. HA), follow-up | 88/94 | NA | NA | 0 (NA; 0.89) | 2 | 0/0.15 | NP |
| Williams AC, 2012 | Cognitive behavioural | AC | Chronic pain (excl. HA), follow-up | 667/667 | 0.46 | 0.14 | 33 (0-65; 0.13) | 12 | 0/2.84 | NP |
| Williams AC, 2012 | Cognitive behavioural | TAU | Chronic pain (excl. HA), follow-up | 325/310 | 0.16 | -0.56 | 10 (0-63; 0.35) | 7 | 0/1.82 | NP |

Abbreviations: AC: Active control; AtC: Attention control; ASMP: Arthritis Self-Management Program; Biofeedback: biofeedback; CBT: cognitive behavioural therapy; EDU: education; EMG Biofeedback: Electromyographic biofeedback; HA: headache; HYP: hypnotherapy; IM: imagination; IT: Intermediate term; LT: Long term; m: months; MBT: Mindfulness-based therapy; MBCT: Mindfulness-based cognitive therapy; MBPSR: multidisciplinary bio-psychosocial rehabilitation programs; MBR: Multidisciplinary biopsychological rehabilitation; MBSR: Mindfulness-based stress reduction; MTMD, Myofascial Temporomandibular Disorder; NA: Not applicable, because only two studies were available; NP: not pertinent, because the expected number of statistically significant studies is larger than the observed; NR: not reported; REL: relaxation; RIMH: relaxation, guided imagery, meditation or hypnosis; SGT: Supportive group therapy; SMP: Self-management education programmes; ST: Short term; St. Care: Standard care; TAU: Treated as usual; UC: Usual Care; WL, Waiting list.

^a^ P-value from the Egger’s regression asymmetry test.

^b^ Summary effect when standard error equals zero, extrapolated from the fitted Egger’s regression line. * Denotes a statistically significant estimate.

^c^ I^2^ metric of inconsistency (95% CI) and the P-value of the Q test.

^d^ Expected number of statistically significant studies using the point estimate of the largest study (smallest standard error) as the plausible effect size.

^e^ Observed/Expected number of statistically significant studies

^f^ P value of the excess statistical significance test. All statistical tests were two-sided.

^g^ Reported only for meta-analyses including at least 3 studies.

**Table S3:** Description and summary effects of the 141 meta-analyses of RCTs investigating the effectiveness of various psychological interventions for pain reduction.

| **Author, Year** | **Intervention Group** | **Control group** | **Type of pain** | **Control/ Intervention N** | **Summary effect (95% Confidence interval) ^a^** | | | **Fixed  P-value ^e^** | **Random P-value ^f^** | **95% Prediction interval ^g^** |
| --- | --- | --- | --- | --- | --- | --- | --- | --- | --- | --- |
|  |  |  |  |  | **Fixed Effects ^b^** | **Random effects ^c^** | **Largest Study ^d^** |  |  |  |
| Adachi T, 2013 | Hypnosis | St. Care | Chronic, post-intervention | 17/29 | 1.08 (0.42, 1.74) | 1.10 (0.17, 2.02) | 0.64 (-0.27, 1.55) | 0.001 | 0.020 | NA |
| Adachi T, 2013 | Hypnosis | Other psychological treatment | Chronic, post-intervention | 92/106 | 0.25 (-0.03, 0.54) | 0.22 (-0.24, 0.68) | 0.60 (-0.03, 1.22) | 0.082 | 0.339 | -1.17, 1.62 |
| Adachi T, 2013 | Hypnosis | Other psychological treatment | Chronic, follow-up | 24/33 | -0.35 (-0.90, 0.19) | -0.35 (-1.42, 0.71) | 0.19 (-0.57, 0.97) | 0.201 | 0.515 | NA |
| Aqqarwal VR, 2011 | Any psychosocial intervention | Usual treatment | Muscle palpation, >3m | 69/74 | -1.09 (-1.56, -0.61) ^h^ | -1.09 (-1.56, -0.61) ^h^ | -1.11 (-1.63, -0.59) ^h^ | 7.0E-06 | 7.0E-06 | -4.15, 1.98 |
| Aqqarwal VR, 2011 | CBT | Usual treatment | Orofacial, >3m | 187/196 | -0.25 (-0.46, -0.05) | -0.25 (-0.46, -0.05) | -0.32 (-0.66, 0.01) | 0.014 | 0.014 | -0.70, 0.19 |
| Aqqarwal VR, 2011 | CBT+Biofeedback | Usual treatment | Orofacial, >3m | 81/115 | -0.52 (-0.82, -0.23) | -0.46 (-0.92, 0.00) | -0.82 (-1.23, -0.41) | 5.5E-04 | 0.049 | -5.22, 4.30 |
| Aqqarwal VR, 2011 | Biofeedback | Usual treatment | Orofacial, ≤3m | 15/30 | -0.40 (-1.06, 0.25) | -0.46 (-1.30, 0.39) | -0.11 (-0.91, 0.70) | 0.227 | 0.289 | NA |
| Aqqarwal VR, 2011 | CBT | Usual treatment | Orofacial, ≤3m | 203/208 | 0.03 (-0.16, 0.23) | 0.03 (-0.16, 0.23) | 0.24 (-0.09, 0.57) | 0.749 | 0.749 | -0.40, 0.46 |
| Aqqarwal VR, 2011 | CBT+Biofeedback | Usual treatment | Orofacial, ≤3m | 36/54 | 0.46 (0.02, 0.90) | 0.40 (-0.23, 1.03) | 0.66 (0.13, 1.19) | 0.042 | 0.214 | NA |
| Aqqarwal VR, 2011 | Hypnosis | REL | Orofacial, ≤3m | 39/42 | -1.84 (-3.26, -0.42) ^h^ | -1.84 (-3.26, -0.42) ^h^ | -1.90 (-3.37, -0.43) ^h^ | 0.011 | 0.011 | NA |
| Bawa F, 2015 | Mindfulness | AC | Chronic pain intensity | 203/146 | 0.09 (-0.13, 0.31) | 0.09 (-0.13, 0.31) | 0.10 (-0.29, 0.49) | 0.420 | 0.420 | -0.27, 0.45 |
| Bawa F, 2015 | Mindfulness | Inactive Control | Chronic pain intensity | 49/55 | 0.38 (-0.01, 0.78) | 0.38 (-0.01, 0.78) | 0.23 (-0.42, 0.88) | 0.055 | 0.055 | -0.48, 1.25 |
| Bernardy K, 2013 | CBT | AC/AtC/EDU/TAU/Support | Fibromyalgia (self-efficacy), end of treatment | 275/314 | -0.38 (-0.55, -0.22) | -0.39 (-0.73, -0.06) | -0.93 (-1.32, -0.54) | 6.2E-06 | 0.022 | -1.50, 0.71 |
| Bernardy K, 2013 | Operant therapy | AC/EDU/TAU | Fibromyalgia (self-efficacy), end of treatment | 40/83 | -0.99 (-1.41, -0.58) | -1.18 (-3.02, 0.65) | -0.26 (-0.80, 0.27) | 2.9E-06 | 0.205 | NA |
| Bernardy K, 2013 | CBT | AC/AtC/EDU/TAU/Support | Fibromyalgia (self-efficacy), LT | 229/265 | -0.49 (-0.68, -0.30) | -0.52 (-1.04, 0.00) | -1.01 (-1.40, -0.61) | 2.4E-07 | 0.049 | -2.32, 1.28 |
| Bernardy K, 2013 | Operant therapy | AC/EDU/TAU | Fibromyalgia (self-efficacy), LT | 40/83 | -1.61 (-2.05, -1.17) | -1.69 (-2.76, -0.62) | -1.16 (-1.73, -0.59) | 5.1E-13 | 0.002 | NA |
| Bernardy K, 2013 | CBT | AC/AtC/EDU/TAU/Support | Fibromyalgia, end of treatment | 547/603 | -0.32 (-0.44, -0.20) | -0.30 (-0.45, -0.15) | -0.62 (-0.89, -0.34) | 9.9E-08 | 7.4E-05 | -0.69, 0.09 |
| Bernardy K, 2013 | Operant therapy | AC/AtC/EDU/Support | Fibromyalgia, end of treatment | 40/83 | -0.53 (-0.93, -0.12) | -0.66 (-2.56, 1.24) | 0.30 (-0.24, 0.83) | 0.011 | 0.494 | NA |
| Bernardy K, 2013 | Self-management | AC/TAU | Fibromyalgia, end of treatment | 94/86 | -0.10 (-0.39, 0.20) | 0.02 (-0.80, 0.84) | -0.38 (-0.75, -0.02) | 0.528 | 0.961 | NA |
| Bernardy K, 2013 | CBT | AC/AtC/EDU/TAU/Support | Fibromyalgia, LT | 360/410 | -0.28 (-0.43, -0.14) | -0.28 (-0.43, -0.14) | -0.37 (-0.74, 0.00) | 1.0E-04 | 1.3E-04 | -0.47, -0.10 |
| Bernardy K, 2013 | Operant therapy | AC/EDU/TAU | Fibromyalgia, LT | 40/83 | -1.21 (-1.63, -0.80) | -1.27 (-2.30, -0.24) | -0.76 (-1.31, -0.21) | 9.3E-09 | 0.015 | NA |
| Birnie K, 2014 | Distraction | NR | Needle-related (children, adolescents), self-reported | 1229/1243 | -0.32 (-0.41, -0.24) | -0.44 (-0.67, -0.21) | 0.09 (-0.08, 0.27) | 8.8E-15 | 2.0E-04 | -1.53, 0.65 |
| Champaneria, 2012 | Psychological intervention | No psychological intervention | Chronic pelvic, ≤3m | 79/77 | -0.92 (-1.77, -0.07) ^h^ | -1.07 (-5.37, 3.22) ^h^ | 1.11 (-0.05, 2.27) ^h^ | 0.035 | 0.624 | NA |
| Champaneria, 2012 | Psychological intervention | No psychological intervention | Chronic pelvic, ≥6m | 76/63 | -1.57 (-2.53, -0.61) ^h^ | -1.70 (-6.10, 2.70) ^h^ | 0.54 (-0.78, 1.86) ^h^ | 0.001 | 0.449 | NA |
| Damen L, 2006 | Biofeedback | Control | HA Post-treatment | 24/25 | 1.08 (-0.06, 2.21) | 1.08 (-0.06, 2.21) | 1.41 (-0.11, 2.93) | 0.063 | 0.063 | NA |
| Damen L, 2006 | REL + CBT | Attention placebo | HA Post-treatment | 34/35 | 0.39 (0.01, 0.77) | 0.39 (0.01, 0.77) | 0.33 (-0.14, 0.79) | 0.045 | 0.045 | NA |
| Damen L, 2006 | REL+Biofeedback | WL | HA Post-treatment | 24/20 | 0.36 (0.09, 0.63) | 0.73 (-0.35, 1.82) | 0.28 (0.00, 0.56) | 0.009 | 0.186 | NA |
| Dixon K, 2007 | CBT/ Stress management/ HYP | NR | Arthritis | 1136/1167 | -0.18 (-0.26, -0.10) | -0.20 (-0.30, -0.10) | -0.15 (-0.28, -0.02) | 2.0E-05 | 8.5E-05 | -0.37, -0.02 |
| Du S, 2011 | ASMP/ Self-management | WL/ UC/ Conventional/No treatment | Chronic musculoskeletal, 12m | 770/800 | -0.13 (-0.24, -0.03) | -0.13 (-0.24, -0.03) | -0.05 (-0.19, 0.08) | 0.008 | 0.008 | -0.30, 0.03 |
| Du S, 2011 | ASMP/ Self-management | WL/ UC/ Conventional/No treatment | Chronic musculoskeletal, 4m | 1279/1689 | -0.22 (-0.30, -0.15) | -0.23 (-0.36, -0.11) | -0.35 (-0.49, -0.20) | 4.1E-09 | 2.9E-04 | -0.59, 0.13 |
| Du S, 2011 | ASMP/ Self-management | WL/ UC/ Conventional/No treatment | Chronic musculoskeletal, 6m | 524/494 | -0.29 (-0.42, -0.16) | -0.29 (-0.42, -0.16) | -0.27 (-0.42, -0.12) | 6.0E-06 | 6.0E-06 | -1.11, 0.53 |
| Eccleston C, 2014 | Psychological therapies (Internet-delivered) | AC/TAU/WL | Chronic (Non-HA), follow-up | 617/585 | -0.29 (-0.41, -0.18) | -0.48 (-1.18, 0.22) | -0.14 (-0.29, 0.02) | 6.9E-07 | 0.181 | -3.83, 2.88 |
| Eccleston C, 2014 | Psychological therapies (Internet-delivered) | AC/TAU/WL | Chronic (Non-HA), post-treatment | 903/882 | -0.31 (-0.4, -0.22) | -0.37 (-0.59, -0.15) | -0.20 (-0.36, -0.05) | 9.4E-11 | 9.9E-04 | -1.12, 0.38 |
| Eccleston C, 2014 | Psychological therapies | Control | Chronic and recurrent HA (children, adolescents), follow-up | 111/140 | 0.16 (0.06, 0.27) | 0.49 (0.08, 0.90) | 0.12 (0.02, 0.23) | 0.002 | 0.019 | -0.76, 1.75 |
| Eccleston C, 2014 | Psychological therapies | Control | Chronic and recurrent HA (children, adolescents), post-treatment | 287/427 | 0.38 (0.26, 0.49) | 0.44 (0.28, 0.60) | 0.32 (0.13, 0.52) | 3.8E-10 | 1.2E-07 | 0.08, 0.80 |
| Eccleston C, 2014 | Psychological therapies | Control | Chronic and recurrent non-HA (children, adolescents), follow-up | 270/273 | -0.04 (-0.21, 0.13) | -0.11 (-0.41, 0.19) | 0.16 (-0.16, 0.47) | 0.644 | 0.469 | -0.98, 0.76 |
| Eccleston C, 2014 | Psychological therapies | Control | Chronic and recurrent non-HA (children, adolescents), post-treatment | 404/448 | -0.36 (-0.50, -0.22) | -0.57 (-0.86, -0.27) | 0.21 (-0.10, 0.51) | 3.4E-07 | 2.0E-04 | -1.63, 0.50 |
| Eccleston C, 2014 | Psychological therapies (Internet-delivered) | AC/TAU/WL | Chronic HA, post-treatment | 72/59 | 1.10 (0.54, 1.65) | 1.10 (0.54, 1.65) | 0.99 (0.35, 1.64) | 1.0E-04 | 1.0E-04 | NA |
| Fisher E, 2014 | CBT/ Biofeedback/ REL/ HYP | WL/ EDU/ St. Care/ Self -monitoring | Chronic (excluding HA) | 310/362 | -0.42 (-0.58, -0.26) | -0.60 (-0.91, -0.29) | 0.21 (-0.10, 0.51) | 1.7E-07 | 1.7E-04 | -1.63, 0.44 |
| Fisher E, 2014 | CBT/ Biofeedback/ REL/ HYP | WL/ EDU/ St. Care/ Self- monitoring | Headache | 289/459 | 0.45 (0.32, 0.59) | 0.50 (0.34, 0.66) | 0.25 (-0.03, 0.54) | 2.9E-11 | 3.9E-10 | 0.18, 0.83 |
| Flanagan E, 2015 | CBT | Medical treatment | General vaginal, post-treatment | 56/87 | 0.28 (-0.06, 0.62) | 0.29 (-0.12, 0.70) | 0.15 (-0.32, 0.63) | 0.105 | 0.166 | -3.37, 3.95 |
| Flanagan E, 2015 | CBT | Medical treatment | Pain on intercourse, 6m | 39/62 | -0.12 (-1.34, 1.09) | 0.00 (-1.63, 1.63) | -0.66 (-2.13, 0.81) | 0.844 | 0.999 | NA |
| Flanagan E, 2015 | CBT | Other psychological treatment | Pain on intercourse, follow-up | 42/41 | -0.46 (-0.90, -0.02) | -0.44 (-1.23, 0.36) | -0.83 (-1.43, -0.23) | 0.041 | 0.281 | NA |
| Flanagan E, 2015 | CBT | Medical treatment | Pain on intercourse, post-treatment | 44/66 | 0.68 (-0.46, 1.83) | 0.86 (-1.37, 3.09) | -0.21 (-1.68, 1.26) | 0.242 | 0.449 | NA |
| Flanagan E, 2015 | CBT | Other psychological treatment | Pain on intercourse, post-treatment | 67/81 | -0.06 (-0.39, 0.27) | -0.06 (-0.41, 0.30) | 0.02 (-0.50, 0.53) | 0.729 | 0.750 | -2.75, 2.63 |
| Flanagan E, 2015 | CBT + behavioural | WL | Pain on intercourse, post-treatment | 58/66 | -0.79 (-1.18, -0.40) | -0.81 (-2.61, 0.98) | 0.10 (-0.44, 0.64) | 6.3E-05 | 0.374 | NA |
| Guzman J, 2002 | Less intensive (<30 h) once or twice weekly MBPSR | NR | Low back, 12m | 177/188 | 0.15 (-0.05, 0.36) | 0.13 (-0.25, 0.51) | 0.14 (-0.10, 0.38) | 0.149 | 0.496 | -3.85, 4.11 |
| Guzman J, 2002 | Other types of MBPSR | NR | Low back, 12m | 205/237 | 0.00 (-0.18, 0.19) | 0.00 (-0.18, 0.19) | -0.05 (-0.28, 0.18) | 0.982 | 0.982 | NA |
| Guzman J, 2002 | Less intensive (<30 h) once or twice weekly MBPSR | NR | Low back, 24-30m | 155/165 | 0.17 (-0.05, 0.39) | 0.17 (-0.05, 0.39) | 0.12 (-0.13, 0.36) | 0.141 | 0.141 | NA |
| Guzman J, 2002 | Intensive (>100h) daily MBPSR with functional restoration | NR | Low back, 24m | 77/90 | -0.39 (-0.70, -0.08) | -0.43 (-0.97, 0.10) | -0.18 (-0.57, 0.22) | 0.013 | 0.114 | NA |
| Guzman J, 2002 | Intensive (>100h) daily MBPSR with functional restoration | NR | Low back, 3-4m | 80/85 | -0.57 (-0.88, -0.26) | -0.57 (-0.88, -0.26) | -0.45 (-0.86, -0.04) | 3.5E-04 | 3.5E-04 | NA |
| Guzman J, 2002 | Less intensive (<30 h) once or twice weekly MBPSR | NR | Low back, 3-6m | 206/214 | -0.12 (-0.31, 0.08) | -0.07 (-0.5, 0.37) | -0.20 (-0.43, 0.03) | 0.231 | 0.758 | -1.81, 1.67 |
| Guzman J, 2002 | Intensive (>100h) daily MBPSR with functional restoration | NR | Low back, 60m | 71/83 | -0.21 (-0.53, 0.11) | -0.24 (-0.74, 0.26) | 0.00 (-0.42, 0.42) | 0.191 | 0.351 | NA |
| Guzman J, 2002 | Less intensive (<30 h) once or twice weekly MBPSR | NR | Low back, at treatment completion | 65/77 | -0.23 (-0.57, 0.11) | -0.22 (-0.89, 0.45) | -0.06 (-0.51, 0.39) | 0.182 | 0.529 | -7.77, 7.34 |
| Henrich J, 2015 | Psychological therapies | Control | Irritable bowel syndrome | 1038/1207 | 0.37 (0.28, 0.45) | 0.40 (0.30, 0.51) | 0.05 (-0.19, 0.28) | 1.5E-17 | 3.3E-14 | 0.09, 0.72 |
| Henschke N, 2011 | Behavioural treatment | UC | Chronic low back, IT | 155/164 | -4.29 (-9.28, 0.69) ^h^ | -4.29 (-9.28, 0.69) ^h^ | -5.00 (-10.56, 0.56) ^h^ | 0.091 | 0.091 | NA |
| Henschke N, 2011 | Behavioural treatment | Group exercise | Chronic low back, IT | 68/69 | 1.18 (-3.16, 5.53) ^h^ | 1.18 (-3.16, 5.53) ^h^ | 0.44 (-4.47, 5.35) ^h^ | 0.594 | 0.594 | NA |
| Henschke N, 2011 | Behavioural treatment + physiotherapy | Physiotherapy | Chronic low back, IT | 16/29 | -0.12 (-0.68, 0.44) ^h^ | -0.12 (-0.68, 0.44) ^h^ | -0.29 (-1.01, 0.44) ^h^ | 0.684 | 0.684 | NA |
| Henschke N, 2011 | CBT | Cognitive therapy | Chronic low back, IT | 19/25 | 0.14 (-0.51, 0.79) | -0.31 (-2.59, 1.98) | 0.81 (0.04, 1.59) | 0.675 | 0.793 | NA |
| Henschke N, 2011 | CBT | Operant therapy | Chronic low back, IT | 65/75 | -0.23 (-0.57, 0.11) | -0.23 (-0.57, 0.11) | -0.10 (-0.54, 0.34) | 0.182 | 0.182 | -2.44, 1.97 |
| Henschke N, 2011 | CBT | Respondent therapy | Chronic low back, IT | 32/30 | 0.44 (-0.07, 0.95) | 0.47 (-0.42, 1.35) | 0.03 (-0.66, 0.72) | 0.094 | 0.301 | NA |
| Henschke N, 2011 | Cognitive therapy | Operant therapy | Chronic low back, IT | 39/43 | 0.13 (-0.31, 0.57) | 0.35 (-0.64, 1.35) | 0.03 (-0.43, 0.49) | 0.572 | 0.485 | NA |
| Henschke N, 2011 | Behavioural treatment | Group exercise | Chronic low back, LT | 67/69 | 0.13 (-4.40, 4.67) ^h^ | 0.13 (-4.40, 4.67) ^h^ | -0.62 (-6.29, 5.05) ^h^ | 0.953 | 0.953 | NA |
| Henschke N, 2011 | CBT | Cognitive therapy | Chronic low back, LT | 18/30 | -0.08 (-0.72, 0.55) | -0.89 (-3.64, 1.86) | 0.44 (-0.26, 1.15) | 0.799 | 0.527 | NA |
| Henschke N, 2011 | CBT | Operant therapy | Chronic low back, LT | 67/73 | -0.31 (-0.65, 0.03) | -0.31 (-0.65, 0.03) | -0.24 (-0.69, 0.21) | 0.073 | 0.073 | -2.51, 1.89 |
| Henschke N, 2011 | Behavioural treatment | UC | Chronic low back, ST | 163/167 | -5.18 (-9.78, -0.57) ^h^ | -5.63 (-11.44, 0.17) ^h^ | -4.00 (-9.05, 1.05) ^h^ | 0.028 | 0.057 | NA |
| Henschke N, 2011 | Behavioural treatment | Group exercise | Chronic low back, ST | 73/73 | -2.31 (-6.33, 1.71) ^h^ | -2.31 (-6.33, 1.71) ^h^ | -3.50 (-8.38, 1.38) ^h^ | 0.260 | 0.260 | NA |
| Henschke N, 2011 | Behavioural treatment + physiotherapy | Physiotherapy | Chronic low back, ST | 18/41 | -0.20 (-0.65, 0.24) ^h^ | -0.13 (-1.01, 0.75) ^h^ | -0.55 (-1.12, 0.02) ^h^ | 0.373 | 0.776 | NA |
| Henschke N, 2011 | Behavioural treatment + physiotherapy | Inpatient rehabilitation | Chronic low back, ST | 214/191 | -0.15 (-0.34, 0.05) | -0.15 (-0.34, 0.05) | -0.17 (-0.37, 0.04) | 0.140 | 0.140 | NA |
| Henschke N, 2011 | CBT | WL | Chronic low back, ST | 110/129 | -0.56 (-0.82, -0.30) | -0.60 (-0.97, -0.23) | -0.54 (-0.93, -0.15) | 2.7E-05 | 0.002 | -1.66, 0.46 |
| Henschke N, 2011 | CBT | Cognitive therapy | Chronic low back, ST | 24/37 | -0.11 (-0.64, 0.42) | -0.24 (-1.35, 0.88) | 0.29 (-0.37, 0.94) | 0.681 | 0.675 | NA |
| Henschke N, 2011 | CBT | Operant therapy | Chronic low back, ST | 76/85 | -0.15 (-0.46, 0.16) | -0.15 (-0.46, 0.16) | -0.18 (-0.61, 0.25) | 0.349 | 0.349 | -2.18, 1.88 |
| Henschke N, 2011 | CBT | Respondent therapy | Chronic low back, ST | 47/50 | 0.10 (-0.30, 0.50) | 0.10 (-0.30, 0.50) | 0.25 (-0.39, 0.89) | 0.641 | 0.641 | -2.50, 2.69 |
| Henschke N, 2011 | Cognitive therapy | WL | Chronic low back, ST | 39/29 | -0.27 (-0.75, 0.22) | -0.27 (-0.79, 0.25) | -0.53 (-1.21, 0.16) | 0.282 | 0.314 | NA |
| Henschke N, 2011 | Cognitive therapy | Operant therapy | Chronic low back, ST | 44/49 | 0.14 (-0.27, 0.55) | 0.41 (-0.63, 1.46) | -0.02 (-0.46, 0.43) | 0.502 | 0.440 | NA |
| Henschke N, 2011 | Operant therapy | WL | Chronic low back, ST | 70/83 | -0.43 (-0.75, -0.11) | -0.43 (-0.75, -0.11) | -0.63 (-1.12, -0.13) | 0.009 | 0.009 | -2.52, 1.66 |
| Henschke N, 2011 | Respondent therapy (EMG Biofeedback) | WL | Chronic low back, ST | 30/34 | -0.80 (-1.32, -0.28) | -0.80 (-1.32, -0.28) | -1.19 (-2.01, -0.37) | 0.002 | 0.002 | -4.17, 2.56 |
| Henschke N, 2011 | Respondent therapy (progressive REL) | WL | Chronic low back, ST | 35/39 | -18.67 (-28.00, -9.33) ^h^ | -19.77 (-34.34, -5.20)^h^ | -10.20 (-23.95, 3.55) ^h^ | 8.9E-05 | 0.008 | -175.53, 135.98 |
| Johannsen M, 2013 | EDU/ RIMH/ SGT | WL/ St. Care/ NR | Breast cancer patients/survivors | 625/875 | 0.29 (0.18, 0.39) | 0.34 (0.18, 0.50) | 0.09 (-0.14, 0.31) | 4.8E-08 | 3.3E-05 | -0.15, 0.82 |
| Kamper SJ, 2014 | MBR | UC | Chronic low back, IT | 369/371 | -0.51 (-0.66, -0.36) | -0.60 (-0.85, -0.34) | -0.24 (-0.50, 0.03) | 1.7E-11 | 5.1E-06 | -1.37, 0.18 |
| Kamper SJ, 2014 | MBR | Physical treatment | Chronic low back, IT | 265/266 | -0.23 (-0.40, -0.05) | -0.28 (-0.54, -0.01) | -0.04 (-0.40, 0.32) | 0.011 | 0.039 | -1.01, 0.45 |
| Kamper SJ, 2014 | MBR | UC | Chronic low back, LT | 373/448 | -0.20 (-0.34, -0.07) | -0.21 (-0.37, -0.04) | -0.32 (-0.60, -0.04) | 0.004 | 0.013 | -0.57, 0.15 |
| Kamper SJ, 2014 | MBR | Physical treatment | Chronic low back, LT | 437/435 | -0.29 (-0.43, -0.15) | -0.51 (-1.04, 0.01) | -0.17 (-0.42, 0.08) | 3.8E-05 | 0.057 | -2.41, 1.39 |
| Kamper SJ, 2014 | MBR | Surgery | Chronic low back, LT | 188/197 | -0.23 (-0.43, -0.02) | -0.25 (-0.54, 0.04) | -0.12 (-0.37, 0.13) | 0.028 | 0.096 | NA |
| Kamper SJ, 2014 | MBR | UC | Chronic low back, ST | 446/433 | -0.47 (-0.60, -0.33) | -0.55 (-0.83, -0.27) | -0.20 (-0.46, 0.05) | 9.5E-12 | 1.0E-04 | -1.44, 0.33 |
| Kamper SJ, 2014 | MBR | Physical treatment | Chronic low back, ST | 851/810 | -0.25 (-0.35, -0.16) | -0.30 (-0.54, -0.06) | -0.15 (-0.36, 0.05) | 3.2E-07 | 0.015 | -1.15, 0.55 |
| Kamper SJ, 2014 | MBR | WL | Chronic low back, ST | 107/106 | -0.69 (-0.98, -0.41) | -0.73 (-1.22, -0.24) | -0.45 (-0.84, -0.06) | 1.2E-06 | 0.003 | -6.10, 4.64 |
| Kisely SR, 2015 | Psychological intervention | No psychological intervention | Chest (frequency), ≤3m | 140/154 | -1.21 (-1.58, -0.84) ^h^ | -2.26 (-4.41, -0.11) ^h^ | -0.09 (-0.57, 0.39) ^h^ | 1.9E-10 | 0.039 | -8.95, 4.42 |
| Kisely SR, 2015 | Psychological intervention | No psychological intervention | Chest (frequency), 3-12m | 80/84 | 0.03 (-0.41, 0.48) ^h^ | -0.81 (-2.36, 0.75) ^h^ | 0.21 (-0.27, 0.69) ^h^ | 0.877 | 0.309 | -7.28, 5.67 |
| Kisely SR, 2015 | Psychological intervention | No psychological intervention | Chest (severity), ≤3m | 86/94 | -0.91 (-1.55, -0.26) ^h^ | -4.64 (-12.18, 2.89) ^h^ | -0.85 (-1.50, -0.20) ^h^ | 0.006 | 0.227 | -34.88, 25.59 |
| Kisely SR, 2015 | Psychological intervention | No psychological intervention | Chest, ≤3m | 82/90 | -0.19 (-0.29, -0.10) | -0.20 (-0.35, -0.05) | -0.21 (-0.34, -0.06) | 5.5E-05 | 0.008 | -1.78, 1.38 |
| Kisely SR, 2015 | Psychological intervention | No psychological intervention | Chest, 3-12m | 61/50 | -0.30 (-0.44, -0.15) | -0.30 (-0.44, -0.15) | -0.29 (-0.49, -0.09) | 6.1E-05 | 6.1E-05 | NA |
| Knittle K, 2010 | Self-regulation | WL/ St. Care/ No Intervention | Rheumatoid Arthritis | 638/678 | 0.18 (0.07, 0.29) | 0.18 (0.07, 0.29) | 0.13 (-0.16, 0.41) | 8.9E-04 | 8.9E-04 | 0.07, 0.30 |
| Koranyi S, 2014 | Psychological intervention | Control | Acute pain after open heart surgery, IT | 210/203 | -0.01 (-0.19, 0.16) | -0.03 (-0.25, 0.19) | 0.16 (-0.11, 0.44) | 0.880 | 0.814 | -0.78, 0.72 |
| Koranyi S, 2014 | Psychological intervention | TAU | Acute pain after open heart surgery, IT | 150/143 | 0.09 (-0.11, 0.29) | 0.09 (-0.11, 0.29) | 0.16 (-0.11, 0.44) | 0.392 | 0.392 | -1.21, 1.39 |
| Koranyi S, 2014 | Psychological intervention | Control | Acute pain after open heart surgery, LT | 143/137 | 0.12 (-0.09, 0.33) | 0.12 (-0.09, 0.33) | 0.06 (-0.25, 0.37) | 0.251 | 0.251 | -1.24, 1.48 |
| Kroon FP, 2014 | SMP | UC/WL/No treatment | Osteoarthritis, ST | 282/473 | -0.26 (-0.41, -0.11) | -0.26 (-0.41, -0.11) | -0.32 (-0.61, -0.03) | 8.2E-04 | 8.2E-04 | -0.47, -0.04 |
| Kroon FP, 2014 | SMP | No SMP | Osteoarthritis, ST | 158/198 | 0.03 (-0.18, 0.25) | 0.04 (-0.28, 0.36) | 0.24 (-0.15, 0.63) | 0.763 | 0.826 | -0.95, 1.02 |
| Kroon FP, 2014 | SMP | UC/WL/No treatment | Osteoarthritis, IT | 981/1290 | -0.17 (-0.26, -0.08) | -0.17 (-0.26, -0.08) | -0.29 (-0.51, -0.07) | 1.6E-04 | 1.6E-04 | -0.27, -0.07 |
| Kroon FP, 2014 | SMP | No SMP | Osteoarthritis, IT | 52/66 | -0.19 (-0.56, 0.19) | -0.19 (-0.56, 0.19) | -0.13 (-0.62, 0.36) | 0.329 | 0.329 | NA |
| Kroon FP, 2014 | SMP | Control | Osteoarthritis, IT | 290/284 | -0.26 (-0.43, -0.09) | -0.26 (-0.43, -0.09) | -0.22 (-0.45, 0.01) | 0.003 | 0.003 | -1.38, 0.86 |
| Kroon FP, 2014 | SMP | Information | Osteoarthritis | 378/373 | -0.07 (-0.21, 0.08) | -0.07 (-0.21, 0.08) | -0.10 (-0.27, 0.06) | 0.371 | 0.371 | -1.00, 0.87 |
| Lakhan S, 2013 | MBT/MBCT | EDU/WL/Support | Fibromyalgia | 111/165 | -0.12 (-0.37, 0.13) | -0.12 (-0.37, 0.13) | -0.11 (-0.48, 0.27) | 0.352 | 0.352 | -0.66, 0.43 |
| Lakhan S, 2013 | MBT/MBCT | EDU/WL/Support | Irritable bowel syndrome | 82/78 | -0.59 (-0.91, -0.27) | -0.59 (-0.91, -0.27) | -0.64 (-1.08, -0.20) | 2.6E-04 | 2.6E-04 | NA |
| Lauche R, 2013 | MBSR | UC | Fibromyalgia Syndrome, LT | 99/91 | -0.01 (-0.30, 0.27) | -0.01 (-0.30, 0.27) | -0.05 (-0.42, 0.32) | 0.927 | 0.927 | NA |
| Lauche R, 2013 | MBSR | AC | Fibromyalgia Syndrome, LT | 89/85 | -0.10 (-0.39, 0.20) | -0.10 (-0.39, 0.20) | -0.13 (-0.51, 0.25) | 0.530 | 0.530 | NA |
| Lauche R, 2013 | MBSR | UC | Fibromyalgia Syndrome, ST | 104/98 | -0.11 (-0.39, 0.17) | -0.11 (-0.39, 0.17) | -0.07 (-0.44, 0.30) | 0.436 | 0.436 | NA |
| Lauche R, 2013 | MBSR | AC | Fibromyalgia Syndrome, ST | 89/85 | -0.22 (-0.53, 0.09) | -0.22 (-0.53, 0.09) | -0.32 (-0.70, 0.06) | 0.159 | 0.159 | NA |
| Macea DD, 2010 | Web-based CBT interventions | Control | Chronic pain | 1476/1482 | 0.23 (0.15, 0.31) | 0.29 (0.15, 0.43) | 0.28 (0.13, 0.42) | 2.7E-08 | 6.3E-05 | -0.07, 0.64 |
| Mustafa M, 2013 | Supportive/expressive group therapy | Usual treatment | Metastatic breast cancer | 104/175 | -0.58 (-0.99, -0.17) ^h^ | -0.58 (-0.99, -0.17) ^h^ | -0.75 (-1.36, -0.14) ^h^ | 0.005 | 0.005 | -3.21, 2.05 |
| Osborn RL, 2006 | EDU | Control | Cancer survivors | 122/128 | 0.23 (-0.02, 0.49) | 0.27 (-0.07, 0.61) | 0.06 (-0.27, 0.40) | 0.073 | 0.125 | -2.99, 3.53 |
| Peerdeman K, 2016 | Imagery | Control/ No treatment | Pain relief | 151/150 | 0.24 (0.01, 0.46) | 0.24 (0.01, 0.46) | 0.20 (-0.17, 0.56) | 0.039 | 0.039 | -0.26, 0.73 |
| Peerdeman K, 2016 | Verbal suggestion | Control/ No treatment | Pain relief | 192/191 | 0.31 (0.16, 0.46) | 0.31 (0.16, 0.46) | 0.24 (0.06, 0.41) | 6.4E-05 | 6.4E-05 | -0.02, 0.64 |
| Peerdeman K, 2016 | Verbal suggestion/ Imagery | Control/ No treatment | Affective pain | 84/85 | 0.34 (0.07, 0.61) | 0.34 (0.07, 0.61) | 0.16 (-0.27, 0.60) | 0.013 | 0.013 | -1.41, 2.09 |
| Roldan-Barraza C, 2014 | Psychosocial Intervention/ Psychosocial Intervention + Usual Treatment | Usual Treatment | MTMD (self-reported), LT | 117/121 | 0.13 (-0.13, 0.38) | 0.23 (-0.25, 0.70) | -0.15 (-0.49, 0.19) | 0.334 | 0.352 | -5.07, 5.52 |
| Roldan-Barraza C, 2014 | Psychosocial Intervention/ Psychosocial Intervention + Usual Treatment | Tailored Usual Treatment | MTMD (self-reported), LT | 210/193 | 0.66 (0.23, 1.09) | 0.66 (0.23, 1.09) | 0.80 (0.14, 1.46) | 0.003 | 0.003 | -2.13, 3.45 |
| Roldan-Barraza C, 2014 | Psychosocial Intervention/ Psychosocial Intervention + Usual Treatment | Usual Treatment | MTMD (self-reported), ST | 210/208 | 0.05 (-0.15, 0.24) | 0.08 (-0.18, 0.34) | -0.10 (-0.44, 0.24) | 0.647 | 0.560 | -0.55, 0.71 |
| Roldan-Barraza C, 2014 | Psychosocial Intervention/ Psychosocial Intervention + Usual Treatment | Tailored Usual Treatment | MTMD (self-reported), ST | 241/229 | 0.03 (-0.15, 0.21) | 0.07 (-0.22, 0.37) | 0.00 (-0.32, 0.32) | 0.766 | 0.633 | -1.12, 1.27 |
| Roldan-Barraza C, 2014 | Psychosocial Intervention/ Psychosocial Intervention + Usual Treatment | Usual Treatment | Muscle Pain, ST | 142/138 | 0.23 (-0.02, 0.48) | 0.23 (-0.02, 0.48) | 0.27 (-0.07, 0.61) | 0.067 | 0.067 | -0.31, 0.77 |
| Sheinfeld Gorin S, 2012 | Psychological intervention | Control | Cancer pain severity | 1957/2313 | 0.34 (0.27, 0.40) | 0.34 (0.23, 0.46) | 0.14 (-0.08, 0.36) | 1.8E-22 | 7.2E-09 | -0.21, 0.89 |
| Theadom A, 2015 | Psychological therapies | UC | Fibromyalgia, 3m | 56/59 | -0.85 (-1.76, 0.06) | -0.85 (-1.76, 0.06) | -0.90 (-1.91, 0.11) | 0.067 | 0.067 | NA |
| Theadom A, 2015 | Psychological therapies | AtC | Fibromyalgia, 3m | 52/63 | 0.13 (-0.24, 0.50) | 0.13 (-0.24, 0.50) | 0.19 (-0.29, 0.67) | 0.494 | 0.494 | NA |
| Theadom A, 2015 | Psychological therapies | UC | Fibromyalgia, 6m | 180/191 | -0.51 (-0.72, -0.30) | -0.52 (-0.76, -0.29) | -0.38 (-0.75, -0.02) | 1.7E-06 | 1.5E-05 | -1.06, 0.02 |
| Theadom A, 2015 | Mindfulness | UC | Fibromyalgia, post-intervention | 68/60 | -0.09 (-0.43, 0.26) | -0.09 (-0.43, 0.26) | -0.07 (-0.47, 0.33) | 0.624 | 0.624 | NA |
| Theadom A, 2015 | Psychological therapies | UC | Fibromyalgia, post-intervention | 215/238 | -0.33 (-0.52, -0.15) | -0.33 (-0.52, -0.15) | -0.23 (-0.60, 0.15) | 4.8E-04 | 4.8E-04 | -0.56, -0.11 |
| Theadom A, 2015 | Psychological therapies | AtC | Fibromyalgia, post-intervention | 140/184 | -0.28 (-0.51, -0.06) | -0.30 (-0.66, 0.05) | -0.66 (-1.10, -0.21) | 0.013 | 0.097 | -1.46, 0.85 |
| Theadom A, 2015 | REL | UC | Fibromyalgia, post-intervention | 34/33 | -1.03 (-1.55, -0.50) | -0.82 (-2.29, 0.64) | -1.54 (-2.19, -0.89) | 1.4E-04 | 0.272 | NA |
| Uman LS, 2013 | Distraction | Control | Needle-related (children, adolescents), Behavioural measures | 75/77 | -0.20 (-0.52, 0.11) | -0.15 (-0.68, 0.39) | -0.39 (-0.78, 0.00) | 0.208 | 0.584 | NA |
| Uman LS, 2013 | Distraction | Control | Needle-related (children, adolescents), observer-reported | 200/247 | -0.82 (-1.02, -0.61) | -0.87 (-1.75, 0.02) | -1.34 (-1.74, -0.95) | 9.1E-15 | 0.054 | -4.29, 2.56 |
| Uman LS, 2013 | CBT-combined | Control | Needle-related (children, adolescents), self-reported | 126/124 | -0.34 (-0.59, -0.09) | -0.59 (-1.62, 0.44) | -0.34 (-0.62, -0.07) | 0.008 | 0.261 | -13.01, 11.83 |
| Uman LS, 2013 | Hypnosis | Control | Needle-related (children, adolescents), self-reported | 79/97 | -1.23 (-1.58, -0.88) | -1.4 (-2.32, -0.47) | 0.09 (-0.57, 0.74) | 3.2E-12 | 0.003 | -4.81, 2.01 |
| Uman LS, 2013 | Parent coaching + child distraction | Standard Care | Needle-related (children, adolescents), self-reported | 328/284 | 0.07 (-0.09, 0.23) | 0.05 (-0.20, 0.30) | 0.09 (-0.08, 0.27) | 0.384 | 0.675 | -2.21, 2.31 |
| Uman LS, 2013 | Preparation and information | Control | Needle-related (children, adolescents), self-reported | 78/76 | -0.34 (-0.66, -0.02) | -0.22 (-1.20, 0.76) | -0.70 (-1.11, -0.30) | 0.040 | 0.665 | NA |
| Uman LS, 2013 | Suggestion | Control | Needle-related (children, adolescents), self-reported | 129/89 | -0.13 (-0.41, 0.15) | -0.13 (-0.41, 0.15) | -0.17 (-0.55, 0.21) | 0.354 | 0.354 | -1.91, 1.65 |
| Uman LS, 2013 | Virtual reality | Standard Care | Needle-related (children, adolescents), self-reported | 23/27 | -0.23 (-0.78, 0.33) | -0.23 (-0.78, 0.33) | -0.29 (-1.02, 0.43) | 0.430 | 0.430 | NA |
| Williams AC, 2012 | Behavioural | TAU | Chronic (excl. HA), post-treatment | 230/254 | -0.10 (-0.29, 0.08) | -0.28 (-0.79, 0.24) | 0.09 (-0.22, 0.40) | 0.265 | 0.295 | -2.19, 1.64 |
| Williams AC, 2012 | Cognitive behavioural | AC | Chronic (excl. HA), post-treatment | 639/696 | -0.09 (-0.20, 0.02) | -0.09 (-0.22, 0.03) | -0.05 (-0.31, 0.21) | 0.101 | 0.146 | -0.39, 0.20 |
| Williams AC, 2012 | Cognitive behavioural | TAU | Chronic (excl. HA), post-treatment | 562/586 | -0.24 (-0.36, -0.13) | -0.21 (-0.37, -0.05) | -0.53 (-0.87, -0.19) | 4.7E-05 | 0.010 | -0.72, 0.29 |
| Williams AC, 2012 | Behavioural | TAU | Chronic pain (excl. HA), follow-up | 88/94 | -0.03 (-0.32, 0.26) | -0.03 (-0.32, 0.26) | -0.05 (-0.44, 0.34) | 0.828 | 0.828 | NA |
| Williams AC, 2012 | Cognitive behavioural | AC | Chronic pain (excl. HA), follow-up | 667/667 | -0.07 (-0.17, 0.04) | -0.08 (-0.21, 0.06) | -0.12 (-0.37, 0.14) | 0.237 | 0.281 | -0.42, 0.26 |
| Williams AC, 2012 | Cognitive behavioural | TAU | Chronic pain (excl. HA), follow-up | 325/310 | -0.09 (-0.25, 0.06) | -0.09 (-0.25, 0.08) | -0.14 (-0.42, 0.15) | 0.243 | 0.311 | -0.38, 0.20 |

Abbreviations: AC: Active control; AtC: Attention control; ASMP: Arthritis Self-Management Program; Biofeedback: biofeedback; CBT: cognitive behavioural therapy; EDU: education; EMG Biofeedback: Electromyographic biofeedback; HA: headache; HYP: hypnotherapy; IM: imagination; IT: Intermediate term; LT: Long term; m: months; MBT: Mindfulness-based therapy; MBCT: Mindfulness-based cognitive therapy; MBPSR: multidisciplinary bio-psychosocial rehabilitation programs; MBR: Multidisciplinary biopsychological rehabilitation; MBSR: Mindfulness-based stress reduction; MTMD, Myofascial Temporomandibular Disorder; NA: Not applicable, because only two studies were available; NR: not reported; REL: relaxation; RIMH: relaxation, guided imagery, meditation or hypnosis; SGT: Supportive group therapy; SMP: Self-management education programmes; ST: Short term; St. Care: Standard care; TAU: Treated as usual; UC: Usual Care; WL, Waiting list.

^a^ All summary point estimates on this table were indicative of pain reduction comparing the intervention to the control group. However, the original meta-analyses reported both positive and negative effects as observed on this table because they used different outcome metrics (e.g., pain reduction or difference in pain levels).

^b^ Fixed effects refers to summary effect (95% CI) using the meta-analysis fixed-effects model.

^c^ Random effects refers to summary effect (95% CI) using the meta-analysis random-effects model.

^d^ Relative risk and 95% confidence interval of largest study (smallest SE) in each meta-analysis.
^e^ P value of summary fixed effects estimate.
^f^ P value of summary random effects estimate.

^g^ Prediction intervals are reported only for meta-analyses including at least 3 studies.

^h^ On these comparisons MD is reported, instead of SMD

**Table S4:** Evaluation of bias and heterogeneity in the 141 meta-analyses of RCTs investigating the effectiveness of various psychological interventions for pain reduction.

| **Author, Year** | **Intervention Group** | **Control group** | **Type of pain** | **Control/ Intervention N** | **Egger’s  P-value ^a, g^** | **Effect for SE=0 ^b, g^** | **I^2^ (95% CI;  P-value) ^c, g^** | **Studies** | **Excess significance ^d^** | |
| --- | --- | --- | --- | --- | --- | --- | --- | --- | --- | --- |
|  |  |  |  |  |  |  |  |  | **O/E ^e^** | **P-value ^f^** |
| Adachi T, 2013 | Hypnosis | St. Care | Chronic, post-intervention | 17/29 | NA | NA | 48 (NA; 0.16) | 2 | 1/1.52 | NP |
| Adachi T, 2013 | Hypnosis | Other psychological treatment | Chronic, post-intervention | 92/106 | 0.31 | 2.95 | 60 (0-82; 0.03) | 6 | 1/5.35 | NP |
| Adachi T, 2013 | Hypnosis | Other psychological treatment | Chronic, follow-up | 24/33 | NA | NA | 74 (NA; 0.05) | 2 | 1/0.32 | 0.29 |
| Aqqarwal VR, 2011 | Any psychosocial intervention | Usual treatment | Muscle palpation, >3m | 69/74 | 0.61 | -0.95 | 0 (0-73; 0.76) | 3 | 1/2.05 | NP |
| Aqqarwal VR, 2011 | CBT | Usual treatment | Orofacial, >3m | 187/196 | 0.91 | -0.30 | 0 (0-68; 0.59) | 4 | 1/3.11 | NP |
| Aqqarwal VR, 2011 | CBT+Biofeedback | Usual treatment | Orofacial, >3m | 81/115 | 0.47 | -1.44 | 53 (0-85; 0.12) | 3 | 1/3 | NP |
| Aqqarwal VR, 2011 | Biofeedback | Usual treatment | Orofacial, ≤3m | 15/30 | NA | NA | 35 (NA; 0.22) | 2 | 0/0.16 | NP |
| Aqqarwal VR, 2011 | CBT | Usual treatment | Orofacial, ≤3m | 203/208 | 0.67 | 0.22 | 0 (0-68; 0.40) | 4 | 0/2.53 | NP |
| Aqqarwal VR, 2011 | CBT+Biofeedback | Usual treatment | Orofacial, ≤3m | 36/54 | NA | NA | 45 (NA; 0.18) | 2 | 1/1.95 | NP |
| Aqqarwal VR, 2011 | Hypnosis | REL | Orofacial, ≤3m | 39/42 | NA | NA | 0 (NA; 0.76) | 2 | 1/1.26 | NP |
| Bawa F, 2015 | Mindfulness | AC | Chronic pain intensity | 203/146 | 0.09 | 0.53 | 0 (0-64; 0.67) | 5 | 0/0.64 | NP |
| Bawa F, 2015 | Mindfulness | Inactive Control | Chronic pain intensity | 49/55 | 0.52 | -0.37 | 0 (0-68; 0.57) | 4 | 1/0.8 | 0.59 |
| Bernardy K, 2013 | CBT | AC/AtC/EDU/TAU/Support | Fibromyalgia (self-efficacy), end of treatment | 275/314 | 0.88 | -0.23 | 74 (41-85; <0.001) | 9 | 5/8.99 | NP |
| Bernardy K, 2013 | Operant therapy | AC/EDU/TAU | Fibromyalgia (self-efficacy), end of treatment | 40/83 | NA | NA | 95 (NA; <0.001) | 2 | 1/0.88 | 1.00 |
| Bernardy K, 2013 | CBT | AC/AtC/EDU/TAU/Support | Fibromyalgia (self-efficacy), LT | 229/265 | 0.82 | -0.20 | 86 (74-91; <0.001) | 8 | 3/8 | NP |
| Bernardy K, 2013 | Operant therapy | AC/EDU/TAU | Fibromyalgia (self-efficacy), LT | 40/83 | NA | NA | 83 (NA; 0.02) | 2 | 2/2 | 1.00 |
| Bernardy K, 2013 | CBT | AC/AtC/EDU/TAU/Support | Fibromyalgia, end of treatment | 547/603 | 0.37 | -0.52* | 30 (0-59; 0.11) | 18 | 4/17.17 | NP |
| Bernardy K, 2013 | Operant therapy | AC/AtC/EDU/Support | Fibromyalgia, end of treatment | 40/83 | NA | NA | 95 (NA; <0.001) | 2 | 1/1.15 | NP |
| Bernardy K, 2013 | Self-management | AC/TAU | Fibromyalgia, end of treatment | 94/86 | NA | NA | 85 (NA; 0.01) | 2 | 1/1.81 | NP |
| Bernardy K, 2013 | CBT | AC/AtC/EDU/TAU/Support | Fibromyalgia, LT | 360/410 | 0.64 | -0.43 | 2 (0-50; 0.43) | 13 | 3/9.36 | NP |
| Bernardy K, 2013 | Operant therapy | AC/EDU/TAU | Fibromyalgia, LT | 40/83 | NA | NA | 83 (NA; 0.01) | 2 | 2/2 | 1.00 |
| Birnie K, 2014 | Distraction | NR | Needle-related (children, adolescents), self-reported | 1229/1243 | 0.13 | 0.05 | 86 (80-89; <0.001) | 24 | 7/3.4 | 0.07 |
| Champaneria, 2012 | Psychological intervention | No psychological intervention | Chronic pelvic, ≤3m | 79/77 | NA | NA | 96 (NA; <0.001) | 2 | 1/1.88 | NP |
| Champaneria, 2012 | Psychological intervention | No psychological intervention | Chronic pelvic, ≥6m | 76/63 | NA | NA | 95 (NA; <0.001) | 2 | 1/0.67 | 1.00 |
| Damen L, 2006 | Biofeedback | Control | HA Post-treatment | 24/25 | NA | NA | 0 (NA; 0.52) | 2 | 0/1.81 | NP |
| Damen L, 2006 | REL + CBT | Attention placebo | HA Post-treatment | 34/35 | NA | NA | 0 (NA; 0.66) | 2 | 0/1.21 | NP |
| Damen L, 2006 | REL+Biofeedback | WL | HA Post-treatment | 24/20 | NA | NA | 76 (NA; 0.04) | 2 | 2/1.14 | 0.51 |
| Dixon K, 2007 | CBT/ Stress management/ HYP | NR | Arthritis | 1136/1167 | 0.02 | -0.05 | 9 (0-47; 0.34) | 20 | 5/5.03 | NP |
| Du S, 2011 | ASMP/ Self-management | WL/ UC/ Conventional/No treatment | Chronic musculoskeletal, 12m | 770/800 | 0.17 | 0.03 | 0 (0-64; 0.45) | 5 | 1/0.71 | 0.53 |
| Du S, 2011 | ASMP/ Self-management | WL/ UC/ Conventional/No treatment | Chronic musculoskeletal, 4m | 1279/1689 | 0.73 | -0.15 | 59 (0-79; 0.02) | 8 | 4/7.94 | NP |
| Du S, 2011 | ASMP/ Self-management | WL/ UC/ Conventional/No treatment | Chronic musculoskeletal, 6m | 524/494 | 0.25 | -0.21 | 0 (0-73; 0.84) | 3 | 3/2.78 | 1.00 |
| Eccleston C, 2014 | Psychological therapies (Internet-delivered) | AC/TAU/WL | Chronic (Non-HA), follow-up | 617/585 | 0.59 | 0.21 | 96 (94-98; <0.001) | 4 | 1/2.17 | NP |
| Eccleston C, 2014 | Psychological therapies (Internet-delivered) | AC/TAU/WL | Chronic (Non-HA), post-treatment | 903/882 | 0.42 | -0.12 | 77 (56-86; <0.001) | 11 | 5/5.96 | NP |
| Eccleston C, 2014 | Psychological therapies | Control | Chronic and recurrent HA (children, adolescents), follow-up | 111/140 | <0.01 | 0.02 | 60 (0-83; 0.04) | 5 | 2/1.4 | 0.62 |
| Eccleston C, 2014 | Psychological therapies | Control | Chronic and recurrent HA (children, adolescents), post-treatment | 287/427 | <0.01 | 0.08 | 25 (0-59; 0.18) | 15 | 7/7.46 | NP |
| Eccleston C, 2014 | Psychological therapies | Control | Chronic and recurrent non-HA (children, adolescents), follow-up | 270/273 | 0.23 | 0.47 | 62 (0-81; 0.01) | 7 | 1/1.95 | NP |
| Eccleston C, 2014 | Psychological therapies | Control | Chronic and recurrent non-HA (children, adolescents), post-treatment | 404/448 | <0.01 | 0.77* | 75 (54-84; <0.001) | 13 | 5/4.58 | 0.78 |
| Eccleston C, 2014 | Psychological therapies (Internet-delivered) | AC/TAU/WL | Chronic HA, post-treatment | 72/59 | NA | NA | 0 (NA; 0.53) | 2 | 2/1.94 | 1.00 |
| Fisher E, 2014 | CBT/ Biofeedback/ REL/ HYP | WL/ EDU/ St. Care/ Self-monitoring | Chronic (excluding HA) | 310/362 | <0.01 | 0.73* | 71 (38-83; <0.001) | 11 | 4/3.65 | 0.76 |
| Fisher E, 2014 | CBT/ Biofeedback/ REL/ HYP | WL/ EDU/ St. Care/ Self-monitoring | Headache | 289/459 | <0.01 | -0.05 | 16 (0-52; 0.26) | 18 | 7/6.65 | 1.00 |
| Flanagan E, 2015 | CBT | Medical treatment | General vaginal, post-treatment | 56/87 | 0.84 | -0.09 | 28 (0-80; 0.25) | 3 | 1/0.51 | 0.43 |
| Flanagan E, 2015 | CBT | Medical treatment | Pain on intercourse, 6m | 39/62 | NA | NA | 39 (NA; 0.20) | 2 | 0/0.67 | NP |
| Flanagan E, 2015 | CBT | Other psychological treatment | Pain on intercourse, follow-up | 42/41 | NA | NA | 69 (NA; 0.07) | 2 | 1/2 | NP |
| Flanagan E, 2015 | CBT | Medical treatment | Pain on intercourse, post-treatment | 44/66 | NA | NA | 72 (NA; 0.06) | 2 | 1/0.16 | 0.15 |
| Flanagan E, 2015 | CBT | Other psychological treatment | Pain on intercourse, post-treatment | 67/81 | 0.85 | -0.75 | 13 (0-76; 0.32) | 3 | 0/0.16 | NP |
| Flanagan E, 2015 | CBT + behavioural | WL | Pain on intercourse, post-treatment | 58/66 | NA | NA | 95 (NA; <0.001) | 2 | 1/0.22 | 0.21 |
| Guzman J, 2002 | Less intensive (<30 h) once or twice weekly MBPSR | NR | Low back, 12m | 177/188 | 0.84 | 0.27 | 53 (0-85; 0.12) | 3 | 0/0.94 | NP |
| Guzman J, 2002 | Other types of MBPSR | NR | Low back, 12m | 205/237 | NA | NA | 0 (NA; 0.45) | 2 | 0/0.23 | NP |
| Guzman J, 2002 | Less intensive (<30 h) once or twice weekly MBPSR | NR | Low back, 24-30m | 155/165 | NA | NA | 0 (NA; 0.40) | 2 | 0/0.63 | NP |
| Guzman J, 2002 | Intensive (>100h) daily MBPSR with functional restoration | NR | Low back, 24m | 77/90 | NA | NA | 65 (NA; 0.09) | 2 | 1/0.72 | 1.00 |
| Guzman J, 2002 | Intensive (>100h) daily MBPSR with functional restoration | NR | Low back, 3-4m | 80/85 | NA | NA | 0 (NA; 0.37) | 2 | 2/1.94 | 1.00 |
| Guzman J, 2002 | Less intensive (<30 h) once or twice weekly MBPSR | NR | Low back, 3-6m | 206/214 | 0.80 | -0.22 | 63 (0-85; 0.04) | 4 | 0/1.59 | NP |
| Guzman J, 2002 | Intensive (>100h) daily MBPSR with functional restoration | NR | Low back, 60m | 71/83 | NA | NA | 58 (NA; 0.12) | 2 | 1/0.1 | 0.10 |
| Guzman J, 2002 | Less intensive (<30 h) once or twice weekly MBPSR | NR | Low back, at treatment completion | 65/77 | 0.92 | -0.42 | 69 (0-89; 0.04) | 3 | 1/0.21 | 0.19 |
| Henrich J, 2015 | Psychological therapies | Control | Irritable bowel syndrome | 1038/1207 | 0.01 | 0.03 | 26 (0-52; 0.09) | 32 | 9/2.22 | <0.001 |
| Henschke N, 2011 | Behavioural treatment | UC | Chronic low back, IT | 155/164 | NA | NA | 0 (NA; 0.57) | 2 | 0/1.35 | NP |
| Henschke N, 2011 | Behavioural treatment | Group exercise | Chronic low back, IT | 68/69 | NA | NA | 0 (NA; 0.53) | 2 | 0/0.12 | NP |
| Henschke N, 2011 | Behavioural treatment + physiotherapy | Physiotherapy | Chronic low back, IT | 16/29 | NA | NA | 0 (NA; 0.46) | 2 | 0/0.62 | NP |
| Henschke N, 2011 | CBT | Cognitive therapy | Chronic low back, IT | 19/25 | NA | NA | 90 (NA; <0.001) | 2 | 2/1.72 | 1.00 |
| Henschke N, 2011 | CBT | Operant therapy | Chronic low back, IT | 65/75 | 0.76 | -0.05 | 0 (0-73; 0.61) | 3 | 0/0.3 | NP |
| Henschke N, 2011 | CBT | Respondent therapy | Chronic low back, IT | 32/30 | NA | NA | 66 (NA; 0.09) | 2 | 1/0.11 | 0.10 |
| Henschke N, 2011 | Cognitive therapy | Operant therapy | Chronic low back, IT | 39/43 | NA | NA | 49 (NA; 0.16) | 2 | 0/0.11 | NP |
| Henschke N, 2011 | Behavioural treatment | Group exercise | Chronic low back, LT | 67/69 | NA | NA | 0 (NA; 0.66) | 2 | 0/0.13 | NP |
| Henschke N, 2011 | CBT | Cognitive therapy | Chronic low back, LT | 18/30 | NA | NA | 91 (NA; <0.001) | 2 | 1/0.91 | 1.00 |
| Henschke N, 2011 | CBT | Operant therapy | Chronic low back, LT | 67/73 | 0.77 | -0.21 | 0 (0-73; 0.88) | 3 | 0/1.05 | NP |
| Henschke N, 2011 | Behavioural treatment | UC | Chronic low back, ST | 163/167 | NA | NA | 20 (NA; 0.26) | 2 | 0/1.16 | NP |
| Henschke N, 2011 | Behavioural treatment | Group exercise | Chronic low back, ST | 73/73 | NA | NA | 0 (NA; 0.40) | 2 | 0/1.27 | NP |
| Henschke N, 2011 | Behavioural treatment + physiotherapy | Physiotherapy | Chronic low back, ST | 18/41 | NA | NA | 73 (NA; 0.05) | 2 | 0/1.8 | NP |
| Henschke N, 2011 | Behavioural treatment + physiotherapy | Inpatient rehabilitation | Chronic low back, ST | 214/191 | NA | NA | 0 (NA; 0.48) | 2 | 0/1.09 | NP |
| Henschke N, 2011 | CBT | WL | Chronic low back, ST | 110/129 | 0.34 | 0.02 | 43 (0-78; 0.14) | 5 | 2/4.12 | NP |
| Henschke N, 2011 | CBT | Cognitive therapy | Chronic low back, ST | 24/37 | NA | NA | 76 (NA; 0.04) | 2 | 0/0.65 | NP |
| Henschke N, 2011 | CBT | Operant therapy | Chronic low back, ST | 76/85 | 0.32 | -0.51 | 0 (0-73; 0.80) | 3 | 0/0.76 | NP |
| Henschke N, 2011 | CBT | Respondent therapy | Chronic low back, ST | 47/50 | 0.27 | 2.97 | 0 (0-73; 0.56) | 3 | 0/0.84 | NP |
| Henschke N, 2011 | Cognitive therapy | WL | Chronic low back, ST | 39/29 | NA | NA | 12 (NA; 0.29) | 2 | 0/1.69 | NP |
| Henschke N, 2011 | Cognitive therapy | Operant therapy | Chronic low back, ST | 44/49 | NA | NA | 71 (NA; 0.07) | 2 | 0/0.1 | NP |
| Henschke N, 2011 | Operant therapy | WL | Chronic low back, ST | 70/83 | 0.32 | -1.81 | 0 (0-73; 0.59) | 3 | 1/2.95 | NP |
| Henschke N, 2011 | Respondent therapy (EMG Biofeedback) | WL | Chronic low back, ST | 30/34 | 0.76 | -2.23 | 0 (0-73; 0.4) | 3 | 1/2.99 | NP |
| Henschke N, 2011 | Respondent therapy (progressive REL) | WL | Chronic low back, ST | 35/39 | 0.53 | 33.89 | 57 (0-86; 0.1) | 3 | 1/1.94 | NP |
| Johannsen M, 2013 | EDU/ RIMH/ SGT | WL/ St. Care/ NR | Breast cancer patients/survivors | 625/875 | 0.06 | -0.05 | 50 (0-71; 0.01) | 15 | 6/2.24 | 0.02 |
| Kamper SJ, 2014 | MBR | UC | Chronic low back, IT | 369/371 | 0.01 | 0.40 | 63 (0-83; 0.02) | 6 | 5/3.95 | 0.67 |
| Kamper SJ, 2014 | MBR | Physical treatment | Chronic low back, IT | 265/266 | 0.17 | 0.31 | 51 (0-75; 0.04) | 9 | 2/0.54 | 0.10 |
| Kamper SJ, 2014 | MBR | UC | Chronic low back, LT | 373/448 | 0.68 | -0.06 | 26 (0-68; 0.23) | 7 | 2/5.94 | NP |
| Kamper SJ, 2014 | MBR | Physical treatment | Chronic low back, LT | 437/435 | 0.26 | 0.54 | 92 (88-95; <0.001) | 9 | 2/3.08 | NP |
| Kamper SJ, 2014 | MBR | Surgery | Chronic low back, LT | 188/197 | NA | NA | 48 (NA; 0.16) | 2 | 1/0.75 | 1.00 |
| Kamper SJ, 2014 | MBR | UC | Chronic low back, ST | 446/433 | 0.29 | -0.08 | 72 (35-84; <0.001) | 9 | 5/3.94 | 0.52 |
| Kamper SJ, 2014 | MBR | Physical treatment | Chronic low back, ST | 851/810 | 0.62 | -0.12 | 80 (65-87; <0.001) | 12 | 3/4.53 | NP |
| Kamper SJ, 2014 | MBR | WL | Chronic low back, ST | 107/106 | 0.78 | -0.17 | 63 (0-88; 0.06) | 3 | 2/2.66 | NP |
| Kisely SR, 2015 | Psychological intervention | No psychological intervention | Chest (frequency), ≤3m | 140/154 | 0.29 | -0.23 | 94 (90-96; <0.001) | 7 | 4/0.45 | <0.001 |
| Kisely SR, 2015 | Psychological intervention | No psychological intervention | Chest (frequency), 3-12m | 80/84 | 0.30 | 0.70 | 75 (0-89; 0.01) | 4 | 1/0.6 | 0.48 |
| Kisely SR, 2015 | Psychological intervention | No psychological intervention | Chest (severity), ≤3m | 86/94 | 0.36 | -0.47 | 65 (0-86; 0.04) | 4 | 2/1.17 | 0.59 |
| Kisely SR, 2015 | Psychological intervention | No psychological intervention | Chest, ≤3m | 82/90 | 0.75 | 0.07 | 58 (0-86; 0.09) | 3 | 2/2.98 | NP |
| Kisely SR, 2015 | Psychological intervention | No psychological intervention | Chest, 3-12m | 61/50 | NA | NA | 0 (NA; 0.95) | 2 | 2/2 | 1.00 |
| Knittle K, 2010 | Self-regulation | WL/ St. Care/ No Intervention | Rheumatoid Arthritis | 638/678 | 0.53 | 0.10 | 0 (0-41; 0.94) | 22 | 1/3.73 | NP |
| Koranyi S, 2014 | Psychological intervention | Control | Acute pain after open heart surgery, IT | 210/203 | 0.55 | 0.46 | 35 (0-78; 0.2) | 4 | 0/1.68 | NP |
| Koranyi S, 2014 | Psychological intervention | TAU | Acute pain after open heart surgery, IT | 150/143 | 0.07 | 0.46 | 0 (0-73; 0.75) | 3 | 0/1.26 | NP |
| Koranyi S, 2014 | Psychological intervention | Control | Acute pain after open heart surgery, LT | 143/137 | 0.89 | -0.01 | 0 (0-73; 0.58) | 3 | 0/0.3 | NP |
| Kroon FP, 2014 | SMP | UC/WL/No treatment | Osteoarthritis, ST | 282/473 | 0.89 | -0.21 | 0 (0-61; 0.43) | 6 | 2/5.09 | NP |
| Kroon FP, 2014 | SMP | No SMP | Osteoarthritis, ST | 158/198 | 0.88 | -0.17 | 52 (0-81; 0.08) | 5 | 0/2.46 | NP |
| Kroon FP, 2014 | SMP | UC/WL/No treatment | Osteoarthritis, IT | 981/1290 | 0.12 | -0.35* | 0 (0-49; 0.77) | 13 | 2/10.85 | NP |
| Kroon FP, 2014 | SMP | No SMP | Osteoarthritis, IT | 52/66 | NA | NA | 0 (NA; 0.73) | 2 | 0/0.33 | NP |
| Kroon FP, 2014 | SMP | Control | Osteoarthritis, IT | 290/284 | 0.27 | -0.11 | 0 (0-73; 0.87) | 3 | 0/2.16 | NP |
| Kroon FP, 2014 | SMP | Information | Osteoarthritis | 378/373 | 0.14 | -0.19 | 0 (0-73; 0.69) | 3 | 0/0.97 | NP |
| Lakhan S, 2013 | MBT/MBCT | EDU/WL/Support | Fibromyalgia | 111/165 | 0.68 | 0.11 | 0 (0-68; 0.72) | 4 | 0/0.59 | NP |
| Lakhan S, 2013 | MBT/MBCT | EDU/WL/Support | Irritable bowel syndrome | 82/78 | NA | NA | 0 (NA; 0.76) | 2 | 2/2 | 1.00 |
| Lauche R, 2013 | MBSR | UC | Fibromyalgia Syndrome, LT | 99/91 | NA | NA | 0 (NA; 0.76) | 2 | 0/0.15 | NP |
| Lauche R, 2013 | MBSR | AC | Fibromyalgia Syndrome, LT | 89/85 | NA | NA | 0 (NA; 0.77) | 2 | 0/0.44 | NP |
| Lauche R, 2013 | MBSR | UC | Fibromyalgia Syndrome, ST | 104/98 | NA | NA | 0 (NA; 0.75) | 2 | 0/0.21 | NP |
| Lauche R, 2013 | MBSR | AC | Fibromyalgia Syndrome, ST | 89/85 | NA | NA | 0 (NA; 0.39) | 2 | 0/1.54 | NP |
| Macea DD, 2010 | Web-based CBT interventions | Control | Chronic pain | 1476/1482 | 0.11 | 0.10 | 45 (0-71; 0.05) | 11 | 3/6.99 | NP |
| Mustafa M, 2013 | Supportive/expressive group therapy | Usual treatment | Metastatic breast cancer | 104/175 | 0.90 | -0.83 | 0 (0-73; 0.57) | 3 | 1/2.94 | NP |
| Osborn RL, 2006 | EDU | Control | Cancer survivors | 122/128 | 0.56 | -0.33 | 38 (0-82; 0.2) | 3 | 1/0.25 | 0.23 |
| Peerdeman K, 2016 | Imagery | Control/ No treatment | Pain relief | 151/150 | 0.28 | -0.20 | 0 (0-68; 0.84) | 4 | 0/1.6 | NP |
| Peerdeman K, 2016 | Verbal suggestion | Control/ No treatment | Pain relief | 192/191 | 0.08 | 0.10 | 0 (0-68; 0.42) | 4 | 2/2.3 | NP |
| Peerdeman K, 2016 | Verbal suggestion/ Imagery | Control/ No treatment | Affective pain | 84/85 | 0.70 | -0.27 | 0 (0-73; 0.58) | 3 | 1/0.78 | 1.00 |
| Roldan-Barraza C, 2014 | Psychosocial Intervention/ Psychosocial Intervention + Usual Treatment | Usual Treatment | MTMD (self-reported), LT | 117/121 | 0.40 | -0.75 | 66 (0-88; 0.05) | 3 | 1/0.77 | 1.00 |
| Roldan-Barraza C, 2014 | Psychosocial Intervention/ Psychosocial Intervention + Usual Treatment | Tailored Usual Treatment | MTMD (self-reported), LT | 210/193 | 0.54 | 2.20 | 0 (0-73; 0.58) | 3 | 2/2.94 | NP |
| Roldan-Barraza C, 2014 | Psychosocial Intervention/ Psychosocial Intervention + Usual Treatment | Usual Treatment | MTMD (self-reported), ST | 210/208 | 0.24 | -0.39 | 37 (0-72; 0.15) | 7 | 0/0.83 | NP |
| Roldan-Barraza C, 2014 | Psychosocial Intervention/ Psychosocial Intervention + Usual Treatment | Tailored Usual Treatment | MTMD (self-reported), ST | 241/229 | 0.11 | -1.24 | 61 (0-85; 0.05) | 4 | 1/0.2 | 0.19 |
| Roldan-Barraza C, 2014 | Psychosocial Intervention/ Psychosocial Intervention + Usual Treatment | Usual Treatment | Muscle Pain, ST | 142/138 | 0.92 | 0.21 | 0 (0-68; 0.74) | 4 | 0/1.97 | NP |
| Sheinfeld Gorin S, 2012 | Psychological intervention | Control | Cancer pain severity | 1957/2313 | 0.67 | 0.28 | 60 (40-71; <0.01) | 38 | 17/9.63 | 0.01 |
| Theadom A, 2015 | Psychological therapies | UC | Fibromyalgia, 3m | 56/59 | NA | NA | 0 (NA; 0.82) | 2 | 0/1.32 | NP |
| Theadom A, 2015 | Psychological therapies | AtC | Fibromyalgia, 3m | 52/63 | NA | NA | 0 (NA; 0.70) | 2 | 0/0.58 | NP |
| Theadom A, 2015 | Psychological therapies | UC | Fibromyalgia, 6m | 180/191 | 0.15 | 0.34 | 20 (0-71; 0.29) | 5 | 2/4.12 | NP |
| Theadom A, 2015 | Mindfulness | UC | Fibromyalgia, post-intervention | 68/60 | NA | NA | 0 (NA; 0.87) | 2 | 0/0.17 | NP |
| Theadom A, 2015 | Psychological therapies | UC | Fibromyalgia, post-intervention | 215/238 | 0.90 | -0.28 | 0 (0-54; 0.60) | 9 | 2/3.1 | NP |
| Theadom A, 2015 | Psychological therapies | AtC | Fibromyalgia, post-intervention | 140/184 | 0.53 | 0.79 | 60 (0-83; 0.04) | 5 | 2/4.97 | NP |
| Theadom A, 2015 | REL | UC | Fibromyalgia, post-intervention | 34/33 | NA | NA | 86 (NA; 0.01) | 2 | 1/2 | NP |
| Uman LS, 2013 | Distraction | Control | Needle-related (children, adolescents), Behavioural measures | 75/77 | NA | NA | 61 (NA; 0.11) | 2 | 0/1.76 | NP |
| Uman LS, 2013 | Distraction | Control | Needle-related (children, adolescents), observer-reported | 200/247 | 0.73 | 0.59 | 94 (90-96; <0.001) | 5 | 2/5 | NP |
| Uman LS, 2013 | CBT-combined | Control | Needle-related (children, adolescents), self-reported | 126/124 | 0.78 | -0.07 | 86 (32-93; <0.001) | 3 | 2/1.62 | 1.00 |
| Uman LS, 2013 | Hypnosis | Control | Needle-related (children, adolescents), self-reported | 79/97 | 0.41 | 0.88 | 85 (61-92; <0.001) | 5 | 4/0.37 | <0.001 |
| Uman LS, 2013 | Parent coaching + child distraction | Standard Care | Needle-related (children, adolescents), self-reported | 328/284 | 0.82 | 0.13 | 24 (0-79; 0.27) | 3 | 0/0.71 | NP |
| Uman LS, 2013 | Preparation and information | Control | Needle-related (children, adolescents), self-reported | 78/76 | NA | NA | 88 (NA; <0.001) | 2 | 1/2 | NP |
| Uman LS, 2013 | Suggestion | Control | Needle-related (children, adolescents), self-reported | 129/89 | 0.49 | 0.30 | 0 (0-73; 0.38) | 3 | 0/0.88 | NP |
| Uman LS, 2013 | Virtual reality | Standard Care | Needle-related (children, adolescents), self-reported | 23/27 | NA | NA | 0 (NA; 0.78) | 2 | 0/0.57 | NP |
| Williams AC, 2012 | Behavioural | TAU | Chronic (excl. HA), post-treatment | 230/254 | 0.15 | 1.40 | 87 (67-92; <0.001) | 5 | 1/0.7 | 0.53 |
| Williams AC, 2012 | Cognitive behavioural | AC | Chronic (excl. HA), post-treatment | 639/696 | 0.57 | 0.08 | 25 (0-60; 0.18) | 14 | 3/1.08 | 0.09 |
| Williams AC, 2012 | Cognitive behavioural | TAU | Chronic (excl. HA), post-treatment | 562/586 | 0.02 | -1.27* | 45 (0-68; 0.03) | 16 | 4/15.61 | NP |
| Williams AC, 2012 | Behavioural | TAU | Chronic pain (excl. HA), follow-up | 88/94 | NA | NA | 0 (NA; 0.89) | 2 | 0/0.15 | NP |
| Williams AC, 2012 | Cognitive behavioural | AC | Chronic pain (excl. HA), follow-up | 667/667 | 0.46 | 0.14 | 33 (0-65; 0.13) | 12 | 0/2.84 | NP |
| Williams AC, 2012 | Cognitive behavioural | TAU | Chronic pain (excl. HA), follow-up | 325/310 | 0.16 | -0.56 | 10 (0-63; 0.35) | 7 | 0/1.82 | NP |

Abbreviations: AC: Active control; AtC: Attention control; ASMP: Arthritis Self-Management Program; Biofeedback: biofeedback; CBT: cognitive behavioural therapy; EDU: education; EMG Biofeedback: Electromyographic biofeedback; HA: headache; HYP: hypnotherapy; IM: imagination; IT: Intermediate term; LT: Long term; m: months; MBT: Mindfulness-based therapy; MBCT: Mindfulness-based cognitive therapy; MBPSR: multidisciplinary bio-psychosocial rehabilitation programs; MBR: Multidisciplinary biopsychological rehabilitation; MBSR: Mindfulness-based stress reduction; MTMD, Myofascial Temporomandibular Disorder; NA: Not applicable, because only two studies were available; NP: not pertinent, because the expected number of statistically significant studies is larger than the observed; NR: not reported; REL: relaxation; RIMH: relaxation, guided imagery, meditation or hypnosis; SGT: Supportive group therapy; SMP: Self-management education programmes; ST: Short term; St. Care: Standard care; TAU: Treated as usual; UC: Usual Care; WL, Waiting list.

^a^ P-value from the Egger’s regression asymmetry test.

^b^ Summary effect when standard error equals zero, extrapolated from the fitted Egger’s regression line. * Denotes a statistically significant estimate.

^c^ I^2^ metric of inconsistency (95% CI) and the P-value of the Q test.

^d^ Expected number of statistically significant studies using the point estimate of the largest study (smallest standard error) as the plausible effect size.

^e^ Observed/Expected number of statistically significant studies

^f^ P value of the excess statistical significance test. All statistical tests were two-sided.

^g^ Reported only for meta-analyses including at least 3 studies.

**Table S5:** Grading of the evidence for all the meta-analyses investigating the effectiveness of various psychological interventions for pain reduction.

| **Author, Year** | **Intervention Group** | **Control group** | **Type of pain** | **Total N** | **Largest Study ^a, b^** | **Summary random effects (95% CI) ^a, c^** | **Random P-value ^d^** | **95% Prediction interval** | **Egger’s  P-value^e^** | **I^2^ (%)** | **Studies** | **Excess significance ^e^** | |
| --- | --- | --- | --- | --- | --- | --- | --- | --- | --- | --- | --- | --- | --- |
|  |  |  |  |  |  |  |  |  |  |  |  | **O/E ^g^** | **P-value ^h^** |
| **Associations supported by strong evidence** | | | | | | | | | | | | | |
| None of the associations studied was supported by strong evidence | | | | | | | | | | | | | |
| **Associations supported by highly suggestive evidence** | | | | | | | | | | | | | |
| Peerdeman K, 2016 | Verbal suggestion | Control/ No treatment | Pain relief | 1061 | 0.24 (0.06, 0.41) | 0.75 (0.50, 1.00) | 4.1E-09 | -0.23, 1.73 | <0.001 | 78 | 18 | 11/7.51 | 0.15 |
| Sielski R, 2016 | Biofeedback/ EMG-Biofeedback | Control | Chronic back, post-treatment | 1059 | 0.33 (0.13, 0.52) | 0.61 (0.44, 0.77) | 7.9E-13 | -0.13, 1.34 | 0.43 | 78 | 22 | 15/19.03 | NP |
| **Associations supported by suggestive evidence** | | | | | | | | | | | | | |
| Eccleston C, 2014 | Psychological therapies (Internet-delivered) | AC/TAU/WL | Chronic (Non-HA), post-treatment | 1785 | -0.20 (-0.36, -0.05) | -0.37 (-0.59, -0.15) | 9.9E-04 | -1.12, 0.38 | 0.42 | 77 | 11 | 5/5.96 | NP |
| Bernardy K, 2013 | CBT | AC/AtC/EDU/TAU/Support | Fibromyalgia, end of treatment | 1150 | -0.62 (-0.89, -0.34) | -0.30 (-0.45, -0.15) | 7.4E-05 | -0.69, 0.09 | 0.37 | 30 | 18 | 4/17.17 | NP |
| Birnie K, 2014 | Distraction | NR | Needle-related (children, adolescents), self-reported | 2472 | 0.09 (-0.08, 0.27) | -0.44 (-0.67, -0.21) | 2.0E-04 | -1.53, 0.65 | 0.13 | 86 | 24 | 7/3.4 | 0.07 |
| Dixon K, 2007 | CBT/ Stress management/ HYP | NR | Arthritis | 2303 | -0.15 (-0.28, -0.02) | -0.20 (-0.30, -0.10) | 8.5E-05 | -0.37, -0.02 | 0.02 | 9 | 20 | 5/5.03 | NP |
| Du S, 2011 | ASMP/ Self-management | WL/ UC/ Conventional/No treatment | Chronic musculoskeletal, 4m | 2968 | -0.35 (-0.49, -0.20) | -0.23 (-0.36, -0.11) | 2.9E-04 | -0.59, 0.13 | 0.73 | 59 | 8 | 4/7.94 | NP |
| Du S, 2011 | ASMP/ Self-management | WL/ UC/ Conventional/No treatment | Chronic musculoskeletal, 6m | 1018 | -0.27 (-0.42, -0.12) | -0.29 (-0.42, -0.16) | 6.0E-06 | -1.11, 0.53 | 0.25 | 0 | 3 | 3/2.78 | 1.00 |
| Glombiewski JA, 2010 | EDU/ CBT/ REL | CBT/ TAU/ WL/ Attention placebo | Fibromyalgia | 1017 | 0.07 (-0.13, 0.26) | 0.41 (0.29, 0.54) | 5.2E-11 | -0.09, 0.92 | <0.001 | 69 | 21 | 14/3.15 | <0.001 |
| Henrich J, 2015 | Psychological therapies | Control | Irritable bowel syndrome | 2245 | 0.05 (-0.19, 0.28) | 0.40 (0.30, 0.51) | 3.3E-14 | 0.09, 0.72 | 0.01 | 26 | 32 | 9/2.22 | <0.001 |
| Johannsen M, 2013 | EDU/ RIMH/ SGT | WL/ St. Care/ NR | Breast cancer patients/survivors | 1770 | 0.09 (-0.14, 0.31) | 0.33 (0.19, 0.47) | 2.5E-06 | -0.08, 0.74 | 0.03 | 38 | 21 | 7/2.71 | 0.01 |
| Knittle K, 2010 | Self-regulation | WL/ St. Care/ No Intervention | Rheumatoid Arthritis | 1316 | 0.13 (-0.16, 0.41) | 0.18 (0.07, 0.29) | 8.9E-04 | 0.07, 0.30 | 0.53 | 0 | 22 | 1/3.73 | NP |
| Kroon FP, 2014 | SMP | UC/WL/No treatment | Osteoarthritis, IT | 2271 | -0.29 (-0.51, -0.07) | -0.17 (-0.26, -0.08) | 1.6E-04 | -0.27, -0.07 | 0.12 | 0 | 13 | 2/10.85 | NP |
| Macea DD, 2010 | Web-based CBT interventions | Control | Chronic pain | 2958 | 0.28 (0.13, 0.42) | 0.29 (0.15, 0.43) | 6.3E-05 | -0.07, 0.64 | 0.11 | 45 | 11 | 3/6.99 | NP |
| Sheinfeld Gorin S, 2012 | Psychological intervention | Control | Cancer pain severity | 4270 | 0.14 (-0.08, 0.36) | 0.34 (0.23, 0.46) | 7.2E-09 | -0.21, 0.89 | 0.67 | 60 | 38 | 17/9.63 | 0.01 |
| **Associations supported by weak evidence** | | | | | | | | | | | | | |
| Adachi T, 2013 | Hypnosis | St. Care | Chronic, post-intervention | 163 | 0.19 (-0.23, 0.60) | 0.60 (0.03, 1.17) | 0.039 | -1.65, 2.85 | 0.22 | 57 | 4 | 1/0.83 | 1 |
| Aqqarwal VR, 2011 | Any psychosocial intervention | Usual treatment | Muscle palpation, >3m | 143 | -1.11 (-1.63, -0.59) ^i^ | -1.09 (-1.56, -0.61) ^i^ | 7.0E-06 | -4.15, 1.98 | 0.61 | 0 | 3 | 1/2.05 | NP |
| Aqqarwal VR, 2011 | CBT | Usual treatment | Orofacial, >3m | 383 | -0.32 (-0.66, 0.01) | -0.25 (-0.46, -0.05) | 0.014 | -0.70, 0.19 | 0.91 | 0 | 4 | 1/3.11 | NP |
| Aqqarwal VR, 2011 | CBT+Biofeedback | Usual treatment | Orofacial, >3m | 196 | -0.82 (-1.23, -0.41) | -0.46 (-0.92, 0.00) | 0.049 | -5.22, 4.30 | 0.47 | 53 | 3 | 1/3 | NP |
| Aqqarwal VR, 2011 | HYP | REL | Orofacial, ≤3m | 81 | -1.90 (-3.37, -0.43) ^i^ | -1.84 (-3.26, -0.42) ^i^ | 0.011 | NA | NA | 0 | 2 | 1/1.26 | NP |
| Bernardy K, 2013 | CBT | AC/AtC/EDU/TAU/Support | Fibromyalgia (self-efficacy), end of treatment | 589 | -0.93 (-1.32, -0.54) | -0.39 (-0.73, -0.06) | 0.022 | -1.50, 0.71 | 0.88 | 74 | 9 | 5/8.99 | NP |
| Bernardy K, 2013 | CBT | AC/AtC/EDU/TAU/Support | Fibromyalgia (self-efficacy), LT | 494 | -1.01 (-1.40, -0.61) | -0.52 (-1.04, 0.00) | 0.049 | -2.32, 1.28 | 0.82 | 86 | 8 | 3/8 | NP |
| Bernardy K, 2013 | Operant therapy | AC/EDU/TAU | Fibromyalgia (self-efficacy), LT | 123 | -1.16 (-1.73, -0.59) | -1.69 (-2.76, -0.62) | 0.002 | NA | NA | 83 | 2 | 2/2 | 1.00 |
| Bernardy K, 2013 | CBT | AC/AtC/EDU/TAU/Support | Fibromyalgia, LT | 770 | -0.37 (-0.74, 0.00) | -0.28 (-0.43, -0.14) | 1.3E-04 | -0.47, -0.10 | 0.64 | 2 | 13 | 3/9.36 | NP |
| Bernardy K, 2013 | Operant therapy | AC/EDU/TAU | Fibromyalgia, LT | 123 | -0.76 (-1.31, -0.21) | -1.27 (-2.30, -0.24) | 0.015 | NA | NA | 83 | 2 | 2/2 | 1.00 |
| Bernardy K, 2011 | HYP | CBT/ TAU/ WL/ Attention placebo | Fibromyalgia | 178 | -1.52 (-2.17, -0.87) | -1.17 (-2.21, -0.13) | 0.028 | -4.83, 2.49 | 0.84 | 88 | 6 | 3/5.99 | NP |
| Damen L, 2006 | Biofeedback | Control | HA Post-treatment | 71 | 1.41 (-0.11, 2.93) | 1.16 (0.25, 2.08) | 0.012 | -4.75, 7.08 | 0.04 | 0 | 3 | 0/2.74 | NP |
| Damen L, 2006 | REL + Biofeedback + CBT | WL | HA Post-treatment | 53 | 0.51 (-0.15, 1.16) | 0.58 (0.02, 1.13) | 0.042 | NA | NA | 0 | 2 | 0/1.4 | NP |
| Damen L, 2006 | REL + CBT | Attention placebo | HA Post-treatment | 69 | 0.33 (-0.14, 0.79) | 0.39 (0.01, 0.77) | 0.045 | NA | NA | 0 | 2 | 0/1.21 | NP |
| Du S, 2011 | ASMP/ Self-management | WL/ UC/ Conventional/No treatment | Chronic musculoskeletal, 12m | 1570 | -0.05 (-0.19, 0.08) | -0.13 (-0.24, -0.03) | 0.008 | -0.30, 0.03 | 0.17 | 0 | 5 | 1/0.71 | 0.53 |
| Eccleston C, 2014 | Psychological therapies | Control | Chronic and recurrent HA (children, adolescents), follow-up | 251 | 0.12 (0.02, 0.23) | 0.49 (0.08, 0.90) | 0.019 | -0.76, 1.75 | <0.001 | 60 | 5 | 2/1.4 | 0.62 |
| Eccleston C, 2014 | Psychological therapies | Control | Chronic and recurrent HA (children, adolescents), post-treatment | 714 | 0.32 (0.13, 0.52) | 0.44 (0.28, 0.60) | 1.2E-07 | 0.08, 0.80 | <0.001 | 25 | 15 | 7/7.46 | NP |
| Eccleston C, 2014 | Psychological therapies | Control | Chronic and recurrent non-HA (children, adolescents), post-treatment | 852 | 0.21 (-0.10, 0.51) | -0.57 (-0.86, -0.27) | 2.0E-04 | -1.63, 0.50 | <0.001 | 75 | 13 | 5/4.58 | 0.78 |
| Eccleston C, 2014 | Psychological therapies (Internet-delivered) | AC/TAU/WL | Chronic HA, post-treatment | 131 | 0.99 (0.35, 1.64) | 1.10 (0.54, 1.65) | 1.0E-04 | NA | NA | 0 | 2 | 2/1.94 | 1.00 |
| Fisher E, 2014 | CBT/ Biofeedback/ REL/ HYP | WL/ EDU/ St. Care/ Self-monitoring | Chronic (excluding HA) | 672 | 0.21 (-0.10, 0.51) | -0.60 (-0.91, -0.29) | 1.7E-04 | -1.63, 0.44 | <0.001 | 71 | 11 | 4/3.65 | 0.76 |
| Fisher E, 2014 | CBT/ Biofeedback/ REL/ HYP | WL/ EDU/ St. Care/ Self-monitoring | Headache | 748 | 0.25 (-0.03, 0.54) | 0.50 (0.34, 0.66) | 3.9E-10 | 0.18, 0.83 | <0.001 | 16 | 18 | 7/6.65 | 1.00 |
| Guzman J, 2002 | Intensive (>100h) daily MBPSR with functional restoration | NR | Low back, 3-4m | 165 | -0.45 (-0.86, -0.04) | -0.57 (-0.88, -0.26) | 3.5E-04 | NA | NA | 0 | 2 | 2/1.94 | 1.00 |
| Henschke N, 2011 | CBT | WL | Chronic low back, ST | 239 | -0.54 (-0.93, -0.15) | -0.6 (-0.97, -0.23) | 0.002 | -1.66, 0.46 | 0.34 | 43 | 5 | 2/4.12 | NP |
| Henschke N, 2011 | Operant therapy | WL | Chronic low back, ST | 153 | -0.63 (-1.12, -0.13) | -0.43 (-0.75, -0.11) | 0.009 | -2.52, 1.66 | 0.32 | 0 | 3 | 1/2.95 | NP |
| Henschke N, 2011 | Respondent therapy (EMG Biofeedback) | WL | Chronic low back, ST | 64 | -1.19 (-2.01, -0.37) | -0.80 (-1.32, -0.28) | 0.002 | -4.17, 2.56 | 0.76 | 0 | 3 | 1/2.99 | NP |
| Henschke N, 2011 | Respondent therapy (progressive REL) | WL | Chronic low back, ST | 74 | -10.20 (-23.95, 3.55) ^i^ | -19.77 (-34.34, -5.20) ^i^ | 0.008 | -175.53, 135.98 | 0.53 | 57 | 3 | 1/1.94 | NP |
| Kamper SJ, 2014 | MBR | UC | Chronic low back, IT | 740 | -0.24 (-0.50, 0.03) | -0.60 (-0.85, -0.34) | 5.1E-06 | -1.37, 0.18 | 0.01 | 63 | 6 | 5/3.95 | 0.67 |
| Kamper SJ, 2014 | MBR | Physical treatment | Chronic low back, IT | 531 | -0.04 (-0.40, 0.32) | -0.28 (-0.54, -0.01) | 0.039 | -1.01, 0.45 | 0.17 | 51 | 9 | 2/0.54 | 0.10 |
| Kamper SJ, 2014 | MBR | UC | Chronic low back, LT | 821 | -0.32 (-0.60, -0.04) | -0.21 (-0.37, -0.04) | 0.013 | -0.57, 0.15 | 0.68 | 26 | 7 | 2/5.94 | NP |
| Kamper SJ, 2014 | MBR | UC | Chronic low back, ST | 879 | -0.20 (-0.46, 0.05) | -0.55 (-0.83, -0.27) | 1.0E-04 | -1.44, 0.33 | 0.29 | 72 | 9 | 5/3.94 | 0.52 |
| Kamper SJ, 2014 | MBR | WL | Chronic low back, ST | 213 | -0.45 (-0.84, -0.06) | -0.73 (-1.22, -0.24) | 0.003 | -6.10, 4.64 | 0.78 | 63 | 3 | 2/2.66 | NP |
| Kamper SJ, 2014 | MBR | Physical treatment | Chronic low back, ST | 1661 | -0.15 (-0.36, 0.05) | -0.3 (-0.54, -0.06) | 0.015 | -1.15, 0.55 | 0.62 | 80 | 12 | 3/4.53 | NP |
| Kisely SR, 2015 | Psychological intervention | No psychological intervention | Chest (frequency), ≤3m | 294 | -0.09 (-0.57, 0.39) ^i^ | -2.26 (-4.41, -0.11) ^i^ | 0.039 | -8.95, 4.42 | 0.29 | 94 | 7 | 4/0.45 | <0.001 |
| Kisely SR, 2015 | Psychological intervention | No psychological intervention | Chest, ≤3m | 172 | -0.21 (-0.34, -0.06) | -0.20 (-0.35, -0.05) | 0.008 | -1.78, 1.38 | 0.75 | 58 | 3 | 2/2.98 | NP |
| Kisely SR, 2015 | Psychological intervention | No psychological intervention | Chest, 3-12m | 111 | -0.29 (-0.49, -0.09) | -0.30 (-0.44, -0.15) | 6.1E-05 | NA | NA | 0 | 2 | 2/2 | 1.00 |
| Kroon FP, 2014 | SMP | UC/WL/No treatment | Osteoarthritis, ST | 755 | -0.32 (-0.61, -0.03) | -0.26 (-0.41, -0.11) | 8.2E-04 | -0.47, -0.04 | 0.89 | 0 | 6 | 2/5.09 | NP |
| Kroon FP, 2014 | SMP | Control | Osteoarthritis, IT | 574 | -0.22 (-0.45, 0.01) | -0.26 (-0.43, -0.09) | 0.003 | -1.38, 0.86 | 0.27 | 0 | 3 | 0/2.16 | NP |
| Lakhan S, 2013 | MBT/MBCT | EDU/WL/Support | Irritable bowel syndrome | 160 | -0.64 (-1.08, -0.2) | -0.59 (-0.91, -0.27) | 2.6E-04 | NA | NA | 0 | 2 | 2/2 | 1.00 |
| Mustafa M, 2013 | Supportive/expressive group therapy | Usual treatment | Metastatic breast cancer | 279 | -0.75 (-1.36, -0.14) ^i^ | -0.58 (-0.99, -0.17) ^i^ | 0.005 | -3.21, 2.05 | 0.90 | 0 | 3 | 1/2.94 | NP |
| Peerdeman K, 2016 | Verbal suggestion/ Imagery | Control/ No treatment | Affective pain | 313 | 0.48 (0.10, 0.87) | 0.45 (0.20, 0.70) | 3.5E-04 | -0.13, 1.04 | 0.35 | 34 | 7 | 4/5.78 | NP |
| Peerdeman K, 2016 | Verbal suggestion | Control/ No treatment | Expected pain | 144 | 0.65 (0.24, 1.06) | 0.66 (0.43, 0.90) | 3.3E-08 | 0.28, 1.04 | 0.66 | 0 | 5 | 3/4.68 | NP |
| Peerdeman K, 2016 | Conditioning | Control/ No treatment | Pain relief | 142 | 0.62 (0.30, 0.94) | 0.65 (0.18, 1.11) | 0.006 | -4.25, 5.55 | 0.92 | 55 | 3 | 2/2.83 | NP |
| Peerdeman K, 2016 | Imagery | Control/ No treatment | Pain relief | 383 | 0.20 (-0.17, 0.56) | 0.28 (0.02, 0.53) | 0.036 | -0.40, 0.95 | 0.23 | 42 | 6 | 1/2.27 | NP |
| Roldan-Barraza C, 2014 | Psychosocial Intervention/ Psychosocial Intervention + Usual Treatment | Tailored Usual Treatment | MTMD (self-reported), LT | 403 | 0.80 (0.14, 1.46) | 0.66 (0.23, 1.09) | 0.003 | -2.13, 3.45 | 0.54 | 0 | 3 | 2/2.94 | NP |
| Sielski R, 2016 | Biofeedback/ EMG-Biofeedback | Control | Chronic back, follow-up | 471 | 0.31 (0.08, 0.54) | 0.62 (0.40, 0.84) | 2.3E-08 | -0.09, 1.34 | 0.08 | 67 | 11 | 9/8.36 | 1.00 |
| Sprenger L, 2011 | Psychoeducation, IM, REL, Biofeedback, CBT | No treatment/ Paediatric standard care | Recurrent abdominal in children | 449 | 1.22 (1.21, 1.23) | 0.73 (0.33, 1.13) | 3.2E-04 | -0.63, 2.10 | 0.04 | 94 | 9 | 4/8.96 | NP |
| Theadom A, 2015 | Psychological therapies | UC | Fibromyalgia, 6m | 371 | -0.38 (-0.75, -0.02) | -0.52 (-0.76, -0.29) | 1.5E-05 | -1.06, 0.02 | 0.15 | 20 | 5 | 2/4.12 | NP |
| Theadom A, 2015 | Psychological therapies | UC | Fibromyalgia, post-intervention | 453 | -0.23 (-0.6, 0.15) | -0.33 (-0.52, -0.15) | 4.8E-04 | -0.56, -0.11 | 0.90 | 0 | 9 | 2/3.1 | NP |
| Uman LS, 2013 | Hypnosis | Control | Needle-related (children, adolescents), self-reported | 176 | 0.09 (-0.57, 0.74) | -1.4 (-2.32, -0.47) | 0.003 | -4.81, 2.01 | 0.41 | 85 | 5 | 4/0.37 | <0.001 |
| Vellemain S, 2010 | Computerized CBT | WL/ EDU | Pain in children and adolescents | 150 | -0.55 (-1.13, 0.03) | -0.41 (-0.74, -0.07) | 0.019 | -1.15, 0.34 | 0.53 | 0 | 4 | 0/3.29 | NP |
| Williams AC, 2012 | Cognitive behavioural | TAU | Chronic (excl. HA), post-treatment | 1148 | -0.53 (-0.87, -0.19) | -0.21 (-0.37, -0.05) | 0.010 | -0.72, 0.29 | 0.02 | 45 | 16 | 4/15.61 | NP |

Abbreviations: AC: Active control; AtC: Attention control; ASMP: Arthritis Self-Management Program; Biofeedback: biofeedback; CBT: cognitive behavioural therapy; EDU: education; EMG Biofeedback: Electromyographic biofeedback; HA: headache; HYP: hypnotherapy; IM: imagination; IT: Intermediate term; LT: Long term; m: months; MBT: Mindfulness-based therapy; MBCT: Mindfulness-based cognitive therapy; MBPSR: multidisciplinary bio-psychosocial rehabilitation programs; MBR: Multidisciplinary biopsychological rehabilitation; MTMD, Myofascial Temporomandibular Disorder; NA: Not applicable, because only two studies were available; NP: not pertinent, because the expected number of statistically significant studies is larger than the observed; NR: not reported; REL: relaxation; RIMH: relaxation, guided imagery, meditation or hypnosis; SGT: Supportive group therapy; SMP: Self-management education programmes; ST: Short term; St. Care: Standard care; TAU: Treated as usual; UC: Usual Care; WL, Waiting list.

^a^ All summary point estimates on this table were indicative of pain reduction comparing the intervention to the control group. However, the original meta-analyses reported both positive and negative effects as observed on this table because they used different outcome metrics (e.g., pain reduction or difference in pain levels).

^b^ On these comparisons MD is reported, instead of SMD.

^c^ Summary effect and 95% confidence interval of largest study (smallest SE) in each meta-analysis.

^d^ Random effects refer to summary effect (95% CI) using the random-effects model.

^e^ P value of summary random effects estimate.

^f^ P-value from the Egger’s regression asymmetry test.

^g^ Expected number of statistically significant studies using the point estimate of the largest study (smallest standard error) as the plausible effect size.

^h^ Observed/Expected number of statistically significant studies

^i^ P value of the excess statistical significance test. All statistical tests were two-sided.

**Table S6:** AMSTAR quality assessment of the 38 included meta-analysis papers

| **Author, Year** | **AMSTAR items ^a, c^** | | | | | | | | | | | **AMSTAR points** | **Study quality ^b^** |
| --- | --- | --- | --- | --- | --- | --- | --- | --- | --- | --- | --- | --- | --- |
|  | **1** | **2** | **3** | **4** | **5** | **6** | **7** | **8** | **9** | **10** | **11** |  |  |
| Adachi T, 2013 | CAN | CAN | 1 | 0 | 0 | 1 | 0 | 0 | 1 | 0 | 0 | 3 | Low |
| Aqqarwal VR, 2011 | CAN | 1 | 1 | 1 | 1 | 1 | 1 | 1 | 1 | 0 | 0 | 8 | High |
| Bawa F, 2015 | CAN | 0 | 1 | 1 | 0 | 1 | 1 | 1 | 1 | 1 | 0 | 7 | Moderate |
| Bernardy K, 2011 | 1 | 1 | 1 | 1 | 1 | 1 | 1 | 1 | 1 | 1 | 0 | 10 | High |
| Bernardy K, 2013 | 1 | 1 | 1 | 1 | 1 | 1 | 1 | 1 | 1 | 1 | 0 | 10 | High |
| Birnie K, 2014 | 1 | CAN | 1 | CAN | 0 | 1 | 1 | 1 | 1 | 0 | 0 | 6 | Moderate |
| Champaneria, 2012 | 1 | 1 | CAN | CAN | 1 | 1 | 1 | 1 | 1 | 0 | 0 | 7 | Moderate |
| Damen L, 2006 | CAN | 1 | 1 | 0 | 0 | 1 | 1 | 1 | 0 | 0 | 0 | 5 | Moderate |
| Dixon K, 2007 | CAN | 1 | 1 | 0 | 1 | 1 | 1 | 0 | 1 | 0 | 0 | 6 | Moderate |
| Du S, 2011 | CAN | 1 | 1 | 0 | 0 | 1 | 1 | 1 | 1 | 0 | 0 | 6 | Moderate |
| Eccleston C, 2014a | 1 | 1 | 1 | 0 | 1 | 1 | 1 | 1 | 1 | 0 | 0 | 8 | High |
| Eccleston C, 2014b | 1 | 1 | 1 | 0 | 1 | 1 | 1 | 1 | 1 | 0 | 0 | 8 | High |
| Fisher E, 2014 | CAN | CAN | 1 | CAN | 0 | 1 | 1 | 1 | 1 | 0 | 0 | 5 | Moderate |
| Flanagan E, 2015 | CAN | 1 | 1 | CAN | 1 | 1 | 1 | 1 | 1 | 0 | 0 | 7 | Moderate |
| Glombiewski JA, 2010 | CAN | 1 | CAN | 0 | 0 | 1 | 1 | 1 | CAN | 1 | 0 | 5 | Moderate |
| Guzman J, 2002 | 1 | 1 | 1 | CAN | 1 | 1 | 1 | 1 | 1 | 0 | 0 | 8 | High |
| Henrich J, 2015 | CAN | 1 | CAN | CAN | 1 | 1 | 1 | 0 | 1 | 0 | 0 | 5 | Moderate |
| Henschke N, 2011 | 1 | 1 | 1 | CAN | 1 | 1 | 1 | 1 | 1 | 0 | 1 | 9 | High |
| Johannsen M, 2013 | CAN | CAN | 1 | 0 | 0 | 1 | 1 | 1 | 1 | 1 | 0 | 6 | Moderate |
| Kamper SJ, 2014 | 1 | 1 | 1 | 0 | 1 | 1 | 1 | 1 | 1 | 1 | 1 | 10 | High |
| Kisely SR, 2015 | 1 | 1 | 1 | 1 | 1 | 1 | 1 | 1 | 1 | 0 | 0 | 9 | High |
| Knittle K, 2010 | 0 | CAN | 1 | 0 | 0 | 1 | 1 | 0 | 1 | 1 | 0 | 5 | Moderate |
| Koranyi S, 2014 | 1 | 1 | 1 | 1 | 1 | 1 | 1 | 1 | 1 | 1 | 0 | 10 | High |
| Kroon FP, 2014 | 1 | 1 | 1 | 1 | 1 | 1 | 1 | 1 | 1 | 1 | 1 | 11 | High |
| Lakhan S, 2013 | CAN | CAN | CAN | 0 | 0 | 1 | 0 | 0 | 0 | 1 | 0 | 2 | Low |
| Lauche R, 2013 | CAN | 1 | 1 | 0 | 1 | 1 | 1 | 1 | 1 | 1 | 1 | 9 | High |
| Macea DD, 2010 | CAN | CAN | CAN | 0 | 0 | 1 | 0 | 0 | 1 | 1 | 0 | 3 | Low |
| Mustafa M, 2013 | 1 | 1 | 1 | CAN | 1 | 1 | 1 | 0 | 1 | 1 | 0 | 8 | High |
| Osborn RL, 2006 | CAN | CAN | CAN | 0 | 0 | 1 | 0 | 0 | CAN | 1 | 0 | 2 | Low |
| Peerdeman K, 2016 | 1 | 1 | 1 | 0 | 0 | 1 | 1 | 0 | 1 | 1 | 0 | 7 | Moderate |
| Roldan-Barraza C, 2014 | CAN | 1 | CAN | 0 | 1 | 1 | 1 | 0 | 1 | 0 | 0 | 5 | Moderate |
| Sheinfeld Gorin S, 2012 | CAN | 1 | 1 | 0 | 0 | 1 | 1 | 1 | 1 | 1 | 0 | 7 | Moderate |
| Sielski R, 2016 | CAN | 1 | CAN | 0 | 0 | 1 | 1 | 1 | 1 | 1 | 0 | 6 | Moderate |
| Sprenger L, 2011 | CAN | CAN | CAN | CAN | 1 | 1 | 1 | 0 | 0 | 0 | 0 | 3 | Low |
| Theadom A, 2015 | 1 | 1 | 1 | 1 | 1 | 1 | 1 | 1 | 1 | 1 | 0 | 10 | High |
| Uman LS, 2013 | 1 | 1 | 1 | 0 | 1 | 1 | 1 | 1 | 1 | 0 | 0 | 8 | High |
| Vellemain S, 2010 | CAN | CAN | 1 | 0 | 0 | 1 | 0 | 0 | 1 | 0 | 0 | 3 | Low |
| Williams AC, 2012 | 1 | CAN | 1 | 0 | 1 | 1 | 1 | 1 | 1 | 0 | 1 | 8 | High |

^a^ 1, Yes; 2, No; CAN, Cannot answer

^b^ Low: 0 to 3 AMSTAR points; Moderate: 4 to 7 AMSTAR points; High: 8 to 11 AMSTAR points

^c^ AMSTAR items:

**1. Was an 'a priori' design provided?** The research question and inclusion criteria should be established before the conduct of the review. *Note: Need to refer to a protocol, ethics approval, or pre-determined/a priori published research objectives to score a “yes.”*

**2. Was there duplicate study selection and data extraction?** There should be at least two independent data extractors and a consensus procedure for disagreements should be in place. *Note: 2 people do study selection, 2 people do data extraction, consensus process or one person checks the other’s work.*

**3. Was a comprehensive literature search performed?** At least two electronic sources should be searched. The report must include years and databases used (e.g., Central, EMBASE, and MEDLINE). Key words and/or MESH terms must be stated and where feasible the search strategy should be provided. All searches should be supplemented by consulting current contents, reviews, textbooks, specialized registers, or experts in the particular field of study, and by reviewing the references in the studies found. *Note: If at least 2 sources + one supplementary strategy used, select “yes” (Cochrane register/Central counts as 2 sources; a grey literature search counts as supplementary).*

**4. Was the status of publication (i.e. grey literature) used as an inclusion criterion?** The authors should state that they searched for reports regardless of their publication type. The authors should state whether or not they excluded any reports (from the systematic review), based on their publication status, language etc. *Note: If review indicates that there was a search for “grey literature” or “unpublished literature,” indicate “yes.” SIGLE database, dissertations, conference proceedings, and trial registries are all considered grey for this purpose. If searching a source that contains both grey and non-grey, must specify that they were searching for grey/unpublished lit.*

**5. Was a list of studies (included and excluded) provided?** A list of included and excluded studies should be provided. *Note: Acceptable if the excluded studies are referenced. If there is an electronic link to the list but the link is dead, select “no.”*

**6. Were the characteristics of the included studies provided?** In an aggregated form such as a table, data from the original studies should be provided on the participants, interventions and outcomes. The ranges of characteristics in all the studies analyzed e.g., age, race, sex, relevant socioeconomic data, disease status, duration, severity, or other diseases should be reported. *Note: Acceptable if not in table format as long as they are described as above.*

**7. Was the scientific quality of the included studies assessed and documented?** 'A priori' methods of assessment should be provided (e.g., for effectiveness studies if the author(s) chose to include only randomized, double-blind, placebo controlled studies, or allocation concealment as inclusion criteria); for other types of studies alternative items will be relevant. *Note: Can include use of a quality scoring tool or checklist, e.g., Jadad scale, risk of bias, sensitivity analysis, etc., or a description of quality items, with some kind of result for EACH study (“low” or “high” is fine, as long as it is clear which studies scored “low” and which scored “high”; a summary score/range for all studies is not acceptable).*

**8. Was the scientific quality of the included studies used appropriately in formulating conclusions?** The results of the methodological rigor and scientific quality should be considered in the analysis and the conclusions of the review, and explicitly stated in formulating recommendations. *Note: Might say something such as “the results should be interpreted with caution due to poor quality of included studies.” Cannot score “yes” for this question if scored “no” for question 7.*

**9. Were the methods used to combine the findings of studies appropriate?** For the pooled results, a test should be done to ensure the studies were combinable, to assess their homogeneity (i.e., Chi-squared test for homogeneity, I2). If heterogeneity exists a random effects model should be used and/or the clinical appropriateness of combining should be taken into consideration (i.e., is it sensible to combine?). *Note: Indicate “yes” if they mention or describe heterogeneity, i.e., if they explain that they cannot pool because of heterogeneity/variability between interventions.*

**10. Was the likelihood of publication bias assessed?** An assessment of publication bias should include a combination of graphical aids (e.g., funnel plot, other available tests) and/or statistical tests (e.g., Egger regression test, Hedges-Olken). *Note: If no test values or funnel plot included, score “no”. Score “yes” if mentions that publication bias could not be assessed because there were fewer than 10 included studies.*

**11. Was the conflict of interest included?** Potential sources of support should be clearly acknowledged in both the systematic review and the included studies. *Note: To get a “yes,” must indicate source of funding or support for the systematic review AND for each of the included studies.*

**Table S7:** Summary of the quality assessment scores performed in the 35 original meta-analysis papers.

| **Categorical Risk of Bias/ methodological quality assessment tools** | | | | | | | |
| --- | --- | --- | --- | --- | --- | --- | --- |
| **First author** | **Tool** | **Subscale** | **Scoring Range ^a^** | **Low/Met/Yes (%)** | **Unclear (%)** | | **High/Unmet/No (%)** |
| Aqqarwal VR, 2011 | Cochrane Handbook guidelines [1] | Random sequence generation | Low-Unclear-High | 36.36 | 63.64 | | 0 |
|  |  | Allocation concealment | Low-Unclear-High | 9.09 | 81.82 | | 9.09 |
|  |  | Blinding of participants/caregivers | Low-Unclear-High | 45.45 | 36.36 | | 18.18 |
|  |  | Incomplete outcome data | Low-Unclear-High | 54.55 | 18.18 | | 27.27 |
|  |  | Selective reporting | Low-Unclear-High | 81.82 | 18.18 | | 0 |
|  |  | Other bias | Low-Unclear-High | 9.09 | 63.64 | | 27.27 |
| Bernardy K, 2013 | Cochrane Handbook guidelines [1] | Adequate sequence generation | Low-Unclear-High | 56.52 | 43.48 | | 0 |
|  |  | Allocation concealment | Low-Unclear-High | 30.43 | 69.57 | | 0 |
|  |  | Incomplete outcome data | Low-Unclear-High | 43.48 | 8.7 | | 47.83 |
|  |  | Free of selective reporting | Low-Unclear-High | 60.87 | 0 | | 39.13 |
|  |  | Blinding of outcome assessor | Low-Unclear-High | 43.48 | 56.52 | | 0 |
| Birnie K, 2014 | Cochrane Handbook guidelines [1] | Random sequence generation | Low-Unclear-High | 50 | 50 | | 0 |
|  |  | Allocation concealment | Low-Unclear-High | 8.33 | 70.83 | | 20.83 |
|  |  | Incomplete outcome data | Low-Unclear-High | 79.17 | 12.5 | | 8.33 |
|  |  | Free of selective reporting | Low-Unclear-High | 4.17 | 54.17 | | 41.67 |
|  |  | Free of other bias | Low-Unclear-High | 4.17 | 45.83 | | 50 |
|  |  | Blinding of participants and personnel | Low-Unclear-High | 0 | 4.17 | | 95.83 |
|  |  | Blinding of outcome assessment | Low-Unclear-High | 0 | 4.17 | | 95.83 |
| Du S, 2011 | Cochrane Back Review Group guidelines [2] | Randomization | Met-Unclear-Unmet | 58.33 | 41.67 | | 0 |
|  |  | Allocation concealment | Met-Unclear-Unmet | 25 | 0 | | 75 |
|  |  | Blinding | Met-Unclear-Unmet | 33.33 | 0 | | 66.67 |
|  |  | Dropout/attrition | Met-Unclear-Unmet | 100 | 0 | | 0 |
|  |  | Intention to treat analysis | Met-Unclear-Unmet | 75 | 0 | | 25 |
|  |  | Baseline comparability | Met-Unclear-Unmet | 100 | 0 | | 0 |
| Eccleston C, 2014a | Cochrane Handbook guidelines [1] | Random sequence generation | Low-Unclear-High | 46.15 | 46.15 | | 7.69 |
|  |  | Allocation concealment | Low-Unclear-High | 30.77 | 53.85 | | 15.38 |
|  |  | Blinding (performance bias and detection bias) | Low-Unclear-High | 84.62 | 15.38 | | 0 |
|  |  | Incomplete outcome data | Low-Unclear-High | 46.15 | 46.15 | | 7.69 |
|  |  | Selective reporting | Low-Unclear-High | 76.92 | 0 | | 23.08 |
|  |  | Other bias | Low-Unclear-High | 100 | 0 | | 0 |
| Eccleston C, 2014b | Cochrane Handbook guidelines [1] | Random sequence generation | Low-Unclear-High | 35.71 | 64.29 | | 0 |
|  |  | Allocation concealment | Low-Unclear-High | 32.14 | 57.14 | | 10.71 |
|  |  | Blinding of outcome assessment | Low-Unclear-High | 21.43 | 78.57 | | 0 |
|  |  | Incomplete outcome data | Low-Unclear-High | 21.43 | 64.29 | | 14.29 |
|  |  | Selective reporting | Low-Unclear-High | 60.71 | 21.43 | | 17.86 |
| Fisher E, 2014 | Cochrane Handbook guidelines [1] | Random sequence generation | Low-Unclear-High | 44.44 | 55.56 | | 0 |
|  |  | Allocation concealment | Low-Unclear-High | 40.74 | 48.15 | | 11.11 |
|  |  | Detection bias | Low-Unclear-High | 77.78 | 22.22 | | 0 |
|  |  | Incomplete outcome data | Low-Unclear-High | 33.33 | 51.85 | | 14.81 |
|  |  | Selective reporting | Low-Unclear-High | 70.37 | 0 | | 29.63 |
| Flanagan E, 2015 | NICE Methodological checklist | Selection bias | Low-Unclear-High | 80 | 20 | | 0 |
|  |  | Performance bias | Low-Unclear-High | 0 | 80 | | 20 |
|  |  | Attrition bias | Low-Unclear-High | 100 | 0 | | 0 |
|  |  | Detection bias | Low-Unclear-High | 20 | 60 | | 20 |
| Henrich J, 2015 | Cochrane Handbook guidelines [1] | Sequence generation | Yes-Unclear-No | 46.88 | 46.88 | | 6.25 |
|  |  | Allocation concealment | Yes-Unclear-No | 28.13 | 62.5 | | 9.38 |
|  |  | Blinding of outcome assessor | Yes-Unclear-No | 34.38 | 46.88 | | 18.75 |
|  |  | Incomplete outcome data | Yes-Unclear-No | 53.13 | 37.5 | | 9.38 |
|  |  | Selective outcome reporting | Yes-Unclear-No | 71.88 | 28.13 | | 0 |
|  |  | Other potential threats to validity | Yes-Unclear-No | 53.13 | 34.38 | | 12.5 |
| Mustafa M, 2013 | Cochrane Handbook guidelines [1] | Random sequence generation | Low-Unclear-High | 66.67 | 33.33 | | 0 |
|  |  | Allocation concealment | Low-Unclear-High | 33.33 | 66.67 | | 0 |
|  |  | Blinding of participants and personnel | Low-Unclear-High | 100 | 0 | | 0 |
|  |  | Blinding of outcome assessment | Low-Unclear-High | 66.67 | 33.33 | | 0 |
|  |  | Incomplete outcome data | Low-Unclear-High | 66.67 | 33.33 | | 0 |
|  |  | Selective reporting | Low-Unclear-High | 100 | 0 | | 0 |
|  |  | Other bias | Low-Unclear-High | 0 | 100 | | 0 |
| Kamper SJ, 2014 | Cochrane Back Review Group guidelines [2] | Random sequence generation | Low-Unclear-High | 67.74 | 32.26 | | 0 |
|  |  | Allocation concealment | Low-Unclear-High | 45.16 | 51.61 | | 3.23 |
|  |  | Blinding of participants | Low-Unclear-High | 0 | 0 | | 100 |
|  |  | Blinding of clinicians | Low-Unclear-High | 0 | 0 | | 100 |
|  |  | Blinding of outcome assessment | Low-Unclear-High | 0 | 0 | | 100 |
|  |  | Incomplete outcome data | Low-Unclear-High | 70.97 | 3.23 | | 25.81 |
|  |  | Intention to treat analysis | Low-Unclear-High | 38.71 | 51.61 | | 9.68 |
|  |  | Selective reporting | Low-Unclear-High | 3.23 | 93.55 | | 3.23 |
|  |  | Comparability of groups at baseline | Low-Unclear-High | 83.87 | 12.9 | | 3.23 |
|  |  | Compliance | Low-Unclear-High | 19.35 | 70.97 | | 9.68 |
|  |  | Co-interventions | Low-Unclear-High | 12.9 | 83.87 | | 3.23 |
|  |  | Timing of assessment | Low-Unclear-High | 100 | 0 | | 0 |
| Kisely SR, 2015 | Cochrane Handbook guidelines [1] | Random sequence generation | Low-Unclear-High | 41.67 | 58.33 | | 0 |
|  |  | Allocation concealment | Low-Unclear-High | 50 | 50 | | 0 |
|  |  | Blinding of participants and personnel | Low-Unclear-High | 0 | 0 | | 100 |
|  |  | Blinding of outcome assessment | Low-Unclear-High | 16.67 | 41.67 | | 41.67 |
|  |  | Incomplete outcome data | Low-Unclear-High | 41.67 | 8.33 | | 50 |
|  |  | Selective reporting | Low-Unclear-High | 91.67 | 8.33 | | 0 |
| Koranyi S, 2014 | Cochrane Handbook guidelines [1] | Random sequence generation | Low-Unclear-High | 60 | 20 | | 20 |
|  |  | Allocation concealment | Low-Unclear-High | 40 | 40 | | 20 |
|  |  | Blinding of medical personnel | Low-Unclear-High | 60 | 40 | | 0 |
|  |  | Blinding of outcome assessment: Observer-reported outcomes | Low-Unclear-High | 100 ^b^ | 0 | | 0 |
|  |  | Blinding of outcome assessment: Participant-reported outcomes | Low-Unclear-High | 0 | 100 | | 0 |
|  |  | Incomplete outcome data | Low-Unclear-High | 60 | 20 | | 20 |
|  |  | Selective reporting | Low-Unclear-High | 40 | 60 | | 0 |
| Kroon FP, 2014 | Cochrane Handbook guidelines [1] | Random sequence generation | Low-Unclear-High | 50 | 36.36 | | 13.67 |
|  |  | Allocation concealment | Low-Unclear-High | 36.36 | 63.64 | | 0 |
|  |  | Blinding of participants and personnel | Low-Unclear-High | 0 | 0 | | 100 |
|  |  | Blinding of outcome assessment | Low-Unclear-High | 0 | 0 | | 100 |
|  |  | Incomplete outcome data | Low-Unclear-High | 40.91 | 0 | | 59.09 |
|  |  | Selective reporting | Low-Unclear-High | 77.27 | 22.73 | | 0 |
|  |  | Other bias | Low-Unclear-High | 59.09 | 0 | | 40.91 |
| Lauche R, 2013 | Cochrane Musculoskeletal Group guidelines [3] | Random sequence generation | Low-Unclear-High | 60 | 0 | | 40 |
|  |  | Allocation concealment | Low-Unclear-High | 60 | 0 | | 40 |
|  |  | Blinding of participants and personnel | Low-Unclear-High | 20 | 40 | | 40 |
|  |  | Blinding of outcome assessment | Low-Unclear-High | 40 | 40 | | 20 |
|  |  | Incomplete outcome data | Low-Unclear-High | 40 | 20 | | 40 |
|  |  | Selective reporting | Low-Unclear-High | 80 | 0 | | 20 |
|  |  | Other bias | Low-Unclear-High | 60 | 0 | | 40 |
| Peerdeman K, 2016 | Cochrane Handbook guidelines [1] | Random sequence generation | Low-Unclear-High | 29.63 | 51.85 | | 18.52 |
|  |  | Allocation concealment | Low-Unclear-High | 11.11 | 70.37 | | 18.52 |
|  |  | Incomplete outcome data | Low-Unclear-High | 40.74 | 51.85 | | 7.41 |
|  |  | Selective reporting | Low-Unclear-High | 70.37 | 22.22 | | 7.41 |
|  |  | Other bias | Low-Unclear-High | 33.33 | 62.96 | | 3.7 |
| Roldán-Barraza C, 2014 | Cochrane Handbook guidelines [1] | Random sequence generation | Low-Unclear-High | 83.33 | 16.67 | | 0 |
|  |  | Allocation concealment | Low-Unclear-High | 58.33 | 41.67 | | 0 |
|  |  | Blinding of participants and personnel | Low-Unclear-High | 100 | 0 | | 0 |
|  |  | Blinding of outcome assessment | Low-Unclear-High | 75 | 25 | | 0 |
|  |  | Incomplete outcome data | Low-Unclear-High | 83.33 | 16.67 | | 0 |
|  |  | Selective reporting | Low-Unclear-High | 75 | 16.67 | | 8.33 |
|  |  | Other bias | Low-Unclear-High | 100 | 0 | | 0 |
| Theadom A, 2015 | Cochrane Handbook guidelines [1] | Random sequence generation | Low-Unclear-High | 89.47 | 10.53 | | 0 |
|  |  | Allocation concealment | Low-Unclear-High | 84.21 | 15.79 | | 0 |
|  |  | Blinding of outcome assessment | Low-Unclear-High | 73.68 | 15.79 | | 10.53 |
|  |  | Incomplete outcome data | Low-Unclear-High | 89.47 | 5.26 | | 5.26 |
|  |  | Selective reporting | Low-Unclear-High | 84.21 | 5.26 | | 10.53 |
| Uman LS, 2013 | Cochrane Handbook guidelines [1] | Random sequence generation | Low-Unclear-High | 42.42 | 57.58 | | 0 |
|  |  | Allocation concealment | Low-Unclear-High | 6.06 | 72.73 | | 21.21 |
|  |  | Incomplete outcome data | Low-Unclear-High | 78.79 | 12.12 | | 9.09 |
|  |  | Free of selective reporting | Low-Unclear-High | 3.03 | 60.61 | | 36.36 |
|  |  | Free of other bias | Low-Unclear-High | 15.15 | 33.33 | | 51.52 |
|  |  | Blinding of participants and personnel | Low-Unclear-High | 0 | 6.06 | | 93.94 |
|  |  | Blinding of outcome assessment | Low-Unclear-High | 3.03 | 6.06 | | 90.91 |
| Williams AC, 2012 ^c^ | Cochrane Handbook guidelines [1] | Random sequence generation | Low-Unclear-High | 43.75 | 25 | | 31.25 |
|  |  | Allocation concealment | Low-Unclear-High | 34.38 | 3.13 | | 62.5 |
|  |  | Incomplete outcome data | Low-Unclear-High | 28.13 | 50 | | 21.88 |
|  |  | Selective reporting | Low-Unclear-High | 84.38 | 0 | | 15.63 |
|  |  | Blinding of outcome assessment | Low-Unclear-High | 31.25 | 6.25 | | 62.5 |
|  |  |  |  | **Yes** | | **No** | |
| Sheinfeld G S, 2012 ^d^ | Physiotherapy Evidence Database (PEDro) coding scheme developed using a Delphi expert consensus technique [4] | The groups were similar at baseline regarding the most important prognostic indicators | Criterion Present (Yes-No) | 11 | | 27 | |
|  |  | Measures of at least one key outcome were obtained from more than 85% of the  subjects initially allocated to groups | Criterion Present (Yes-No) | 16 | | 22 | |
|  |  | All subjects for whom outcome measures were available received the treatment  or control condition as allocated, or where this was not the case, data for at least one key outcome were analyzed by intention to treat | Criterion Present (Yes-No) | 11 | | 27 | |
|  |  | Point measures and measures of variability for at least one key outcome | Criterion Present (Yes-No) | 10 | | 28 | |
|  |  | Adequate treatment fidelity protocol, including manualized treatment | Criterion Present (Yes-No) | 25 | | 13 | |
|  |  | Adequate treatment fidelity protocol, including monitoring of treatment implementation | Criterion Present (Yes-No) | 31 | | 7 | |
|  |  | Loss to follow-up information is provided | Criterion Present (Yes-No) | 11 | | 27 | |
| **Continuous Risk of Bias/ methodological quality assessment tools** | | | | | | | |
| **First author** | **Tool** | **Subscale** | **Scoring Range ^a^** | **Median (Min - Max)** | | | |
| Adachi T, 2013 | Yates quality scale [5] | Treatment Quality | 0-9 | 4 (2, 7) ^e^ | | | |
|  |  | Methods quality | 0-26 | 11 (6, 15) ^e^ | | | |
|  |  | Overall | 0-35 | 15 (11, 21) ^e^ | | | |
| Bawa F, 2015 | Yates quality scale [5] | Treatment Quality | 0-9 | 7 (2, 8) | | | |
|  |  | Methods quality | 0-26 | 16 (8, 23) | | | |
|  |  | Overall | 0-35 | 23 (10, 31) | | | |
| Bernardy K, 2011 | Yates quality scale [5] | Treatment quality | 0-9 | 6 (0 - 9) | | | |
|  |  | Methods quality | 0-26 | 16.5 (10 - 20) | | | |
| Champaneria , 2012 | Seven points scoring scheme | NA | 0-7 | 6.5 (6 - 7) ^f^ | | | |
| Damen L, 2006 | Modified Delphi list [4] | NA | 0-10 | 4 (2 - 5) | | | |
| Dixon K, 2007 | Internal study validity | NA | 0-3 | 2 (1 - 3) | | | |
|  | External study validity | NA | 0-5 | 4 (2 - 5) | | | |
| Glombiewski JA, 2010 | Adapted Jadad score for pharmacological trials following PRISMA recommendations | NA | 0-20 | 13 (5 - 16) | | | |
| Guzman J, 2002 | Cochrane Back Review Group guidelines [6] | NA | 0-10 | 4 (3 - 6) | | | |
| Henschke N, 2011 | Cochrane Back Review Group guidelines [2] | NA | 0-12 | 5 (3 - 11) | | | |
| Johannsen M, 2013 | Jadad score [7] | NA | 0-5 | 2 (0 - 3) | | | |
|  | Modified Jadad score | NA | 0-12 | 5 (1 - 7) | | | |
| Knittle K, 2010 | 29-item version of the CCDAN scale [8] | NA | 0-87 | 33 (21 - 42) | | | |
| Lakhan S, 2013 | NA | NA | NA | No quality assessment | | | |
| Macea DD, 2010 | NA | NA | NA | All studies except one were deemed to be of adequate quality | | | |
| Osborn RL, 2006 | Modified Jadad score | NA | 0-5 | Only studies with score ≥4 were included | | | |
| Sprenqer L, 2011 | Internal and external validity evaluation | NA | 0-12 | 8 (5 - 11) | | | |
| Sielski R, 2016 | Adapted Jadad score for pharmacological trials following PRISMA recommendations | NA | 0-20 | 11 (2, 17) | | | |
| Vellemain S, 2010 | NA | NA | NA | No quality assessment | | | |
| Williams AC, 2012 ^c^ | Yates quality scale [5] | NA | 0-35 | 23 (10 - 32) | | | |

Abbreviations: NA, Not Applicable.

^a^ Description of Scale Ranges:

*Tools using Low-Unclear-High scoring:* Low indicates that on the corresponding subscale studies are deemed as low risk of bias. Unclear indicates that on the corresponding subscale studies are deemed as unclear risk of bias. High indicates that on the corresponding subscale studies are deemed as high risk of bias.

*Tools using Met-Unclear-Unmet scoring:* Met indicates that on the corresponding subscale studies meet the criteria for low risk of bias. Unclear indicates that on the corresponding subscale it is unclear if studies meet the criteria for low risk of bias. Unmet indicates that on the corresponding subscale studies do not meet the criteria for low risk of bias.

*Tools using Yes-Unclear-No:* Yes indicates that the studies meet the corresponding subscale. Unclear indicates that it is unclear if the studies meet the corresponding subscale. No indicates that indicates that the studies do not meet the corresponding subscale.

*Tools using a continuous score:* On all quality assessment/ risk of bias tools using a continuous scoring higher values indicate higher study quality/ lower risk of bias.

^b^ Only 1 study.

^c^ Same study.

^d^ Study presented results only of PEDro items with sufficient variability (20% to 80% of studies).

^e^ Data are mean (minimum, maximum).

^f^ Only 2 studies.

**References:**

1. Green S: **Cochrane handbook for systematic reviews of interventions version 5.1. 0 [updated March 2011]**. *The Cochrane Collaboration* 2011.

2. Furlan AD, Pennick V, Bombardier C, van Tulder M, Editorial Board CBRG: **2009 updated method guidelines for systematic reviews in the Cochrane Back Review Group**. *Spine (Phila Pa 1976)* 2009, **34**(18):1929-1941.

3. Maxwell L, Santesso N, Tugwell PS, Wells GA, Judd M, Buchbinder R: **Method guidelines for Cochrane Musculoskeletal Group systematic reviews**. *J Rheumatol* 2006, **33**(11):2304-2311.

4. Verhagen AP, de Vet HC, de Bie RA, Kessels AG, Boers M, Bouter LM, Knipschild PG: **The Delphi list: a criteria list for quality assessment of randomized clinical trials for conducting systematic reviews developed by Delphi consensus**. *J Clin Epidemiol* 1998, **51**(12):1235-1241.

5. Yates SL, Morley S, Eccleston C, de CWAC: **A scale for rating the quality of psychological trials for pain**. *Pain* 2005, **117**(3):314-325.

6. van Tulder MW, Assendelft WJ, Koes BW, Bouter LM: **Method guidelines for systematic reviews in the Cochrane Collaboration Back Review Group for Spinal Disorders**. *Spine (Phila Pa 1976)* 1997, **22**(20):2323-2330.

7. Jadad AR, Moore RA, Carroll D, Jenkinson C, Reynolds DJ, Gavaghan DJ, McQuay HJ: **Assessing the quality of reports of randomized clinical trials: is blinding necessary?** *Control Clin Trials* 1996, **17**(1):1-12.

8. Moher D, Cook DJ, Jadad AR, Tugwell P, Moher M, Jones A, Pham B, Klassen TP: **Assessing the quality of reports of randomised trials: implications for the conduct of meta-analyses**. *Health Technol Assess* 1999, **3**(12):i-iv, 1-98.
